# Adolescent Exposure to a THC‐Rich Cannabis Extract Produces Genotype‐Dependent Effects on Cognition and Glial Morphology in Serine Racemase Mutant Mice

**DOI:** 10.1111/jnc.70559

**Published:** 2026-09-25

**Authors:** Igor F. Rangel, Melissa Chaves, Iohanna Pagnoncelli, Isabelle Medeiros, Felipe Pinheiro, Lorena Fortuna, Luiza Castello‐Branco, Ernesto Diaz Roch, Virgínia Martins Carvalho, Flavia Regina Souza Lima, Rogério Panizzutti, Luciana Romão

**Affiliations:** ^1^ Instituto de Ciências Biomédicas Universidade Federal do Rio de Janeiro Rio de Janeiro Brazil; ^2^ Faculdade de Farmácia Universidade Federal do Rio de Janeiro Rio de Janeiro Brazil; ^3^ Instituto de Psiquiatria Universidade Federal do Rio de Janeiro Rio de Janeiro Brazil

**Keywords:** astrocytes, cannabinoids, D‐serine, microglia, neuroinflammation, schizophrenia, serine racemase

## Abstract

Adolescent cannabis exposure has been associated with an increased risk of schizophrenia; however, how genetic vulnerability shapes the long‐term consequences of adolescent cannabinoid exposure remains poorly understood. Δ^9^‐Tetrahydrocannabinol (THC), the principal psychoactive constituent of cannabis and the predominant cannabinoid in the THC‐rich cannabis extract used here, primarily activates cannabinoid type 1 receptors (CB1). In parallel, reduced availability of D‐serine, an endogenous co‐agonist of N‐methyl‐D‐aspartate receptors (NMDARs), has been implicated in the pathophysiology of schizophrenia. Here, we investigated whether adolescent exposure to a THC‐rich cannabis extract modulated long‐term behavioral, neurochemical, and glial outcomes in serine racemase mutant (SrrY269*) mice, a genetic model characterized by reduced D‐serine levels and schizophrenia‐relevant phenotypes. Mice received escalating oral doses of the THC‐rich cannabis extract during adolescence and were evaluated in adulthood using behavioral assays, amino acid quantification, and glial morphometric analyses. Adolescent exposure to the THC‐rich cannabis extract prevented deficits in prepulse inhibition and spatial object recognition memory in adult SrrY269* mice, while transiently impairing sensorimotor gating in wild‐type animals. Irrespective of genotype, mice exposed to the THC‐rich cannabis extract spent more time in the center of the open field in adulthood. In behaviorally tested SrrY269* mice, reduced hippocampal microglial branching was attenuated following adolescent treatment. In an independent behavior‐naïve cohort, SrrY269* mice exhibited marked astrocytic morphological alterations, several of which were also attenuated by adolescent exposure to the THC‐rich cannabis extract. In contrast, the same treatment induced a distinct astrocytic phenotype in wild‐type mice. Adolescent exposure to the THC‐rich cannabis extract did not alter hippocampal or prefrontal cortical levels of D‐serine or other amino acids involved in NMDAR signaling in adulthood. These findings indicate that adolescent exposure to a THC‐rich cannabis extract produces genotype‐dependent long‐term effects, preventing specific behavioral deficits and attenuating independently assessed microglial and astrocytic alterations in SrrY269* mice, while producing adverse behavioral and astrocytic outcomes in wild‐type animals. Genetic background may therefore be an important determinant of the long‐term neurodevelopmental consequences of adolescent exposure to THC‐rich cannabis extracts.

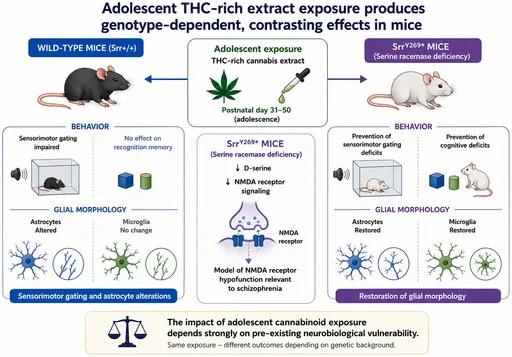

## Graphical abstract

Adolescent exposure to a THC‐rich cannabis extract produced genotype‐dependent, contrasting effects in mice. In wild‐type animals, it altered behavior and brain glial cell structure, whereas in mice with a genetic deficiency in serine racemase, a model of impaired NMDA receptor signaling relevant to schizophrenia, it prevented cognitive and sensorimotor deficits and restored normal astrocyte and microglial morphology. These findings show that the impact of adolescent cannabinoid exposure depends strongly on pre‐existing neurobiological vulnerability.

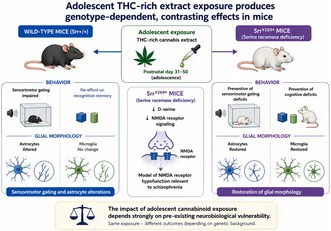

**Abbreviations**
BSA: bovine serum albumin
cat. no.: catalog number
CB1: cannabinoid receptor 1
CBD: cannabidiol
CBN: cannabinol
*Cnr1*
: cannabinoid receptor type 1 gene
Ctrl: control
DAPI: 4′,6‐diamidino‐2‐phenylindole
ECS: endocannabinoid system
GFAP: glial fibrillary acidic protein
HIP: hippocampus
HPLC: high‐performance liquid chromatography
Iba‐1: ionized calcium‐binding adapter molecule 1
LSD: least significant difference
MAM: methylazoxymethanol acetate
NMDAR: N‐methyl‐D‐aspartate receptor
ORT: object recognition task
PBS: phosphate‐buffered saline
PFC: prefrontal cortex
Poly I:C: polyriboinosinic‐polyribocytidylic acid
PPI: prepulse inhibition
RRID: Research Resource Identifier
SR: serine racemase
Srr: serine racemase gene
Srr+/+: wild‐type mice
SrrY269*
: serine racemase Y269* mutant mice
THC: Δ9‐tetrahydrocannabinol

## 1 Introduction

Schizophrenia is a severe psychiatric disorder affecting approximately 1% of the global population (Saha et al. 2005). It is characterized by positive symptoms, including delusions and hallucinations, negative symptoms such as apathy and social withdrawal, and cognitive impairment, one of the strongest determinants of long‐term functional outcome (Marder and Cannon 2019). Current evidence indicates that the disorder arises from complex interactions between genetic susceptibility and environmental factors acting during critical periods of neurodevelopment.

Among the genes associated with susceptibility to schizophrenia, the serine racemase gene (*SRR*) has received particular attention because of its central role in D‐serine metabolism and N‐methyl‐D‐aspartate receptor (NMDAR) function (Wolosker et al. 1999). Genetic association studies have identified variants within the SRR locus associated with schizophrenia susceptibility, while converging clinical and experimental evidence implicates impaired D‐serine signaling in the pathophysiology of schizophrenia (Morita et al. 2007). Although pathogenic SRR mutations have not been identified in affected individuals, the available genetic and functional evidence supports the involvement of SRR‐related pathways in schizophrenia susceptibility.

D‐Serine is an endogenous co‐agonist at the glycine‐binding site of N‐methyl‐D‐aspartate receptors (NMDARs). Impaired NMDAR function is considered an important neurochemical mechanism underlying schizophrenia (Gonzalez‐Burgos and Lewis 2012; Moghaddam and Javitt 2012). Reduced D‐serine concentrations have been reported in the plasma and cerebrospinal fluid of individuals with schizophrenia (Hashimoto et al. 2005; Bendikov et al. 2007; Calcia et al. 2012). Clinical studies have also shown that D‐serine supplementation may improve cognitive performance and negative symptoms (Heresco‐Levy et al. 2005; Kantrowitz et al. 2010, 2016). Panizzutti et al. (2019) further demonstrated that increases in serum D‐serine levels were associated with cognitive improvement. Collectively, these findings implicate disrupted D‐serine/NMDAR signaling in the pathophysiology of schizophrenia, particularly in its cognitive manifestations.

Serine racemase mutant mice (SrrY269*) exhibit an approximately 90% reduction in D‐serine levels in the hippocampus and prefrontal cortex, two brain regions critically involved in schizophrenia. Consistent with this neurochemical phenotype, these animals display schizophrenia‐relevant behavioral alterations, including deficits in prepulse inhibition (PPI), social interaction, and cognition (Labrie, Fukumura, et al. 2009), together with structural abnormalities that parallel findings reported in patients, such as reduced hippocampal volume, decreased dendritic spine density (Balu et al. 2013), and ventricular enlargement (Puhl et al. 2015).

While widely used neurodevelopmental models based on environmental insults, such as prenatal exposure to methylazoxymethanol acetate (MAM) or maternal immune activation with Poly I:C, primarily recapitulate early developmental disruptions (Juckel et al. 2011; Di Bartolomeo et al. 2023), the SrrY269* mouse provides a genetically defined model directly linked to NMDAR hypofunction through reduced D‐serine availability (Balu et al. 2013; Labrie, Fukumura, et al. 2009; Labrie, Clapcote, and Roder 2009; Wolosker et al. 1999). This genetic specificity offers an opportunity to examine the contribution of reduced D‐serine signaling to adult phenotypes associated with NMDAR hypofunction. Importantly, this reductionist approach complements environmentally induced models by enabling the investigation of the long‐term consequences of adolescent cannabinoid exposure in the context of a well‐defined molecular alteration affecting D‐serine/NMDAR signaling.

Cannabis use during adolescence has been consistently identified as an environmental risk factor for schizophrenia. Epidemiological studies report a dose‐dependent association between adolescent cannabis use and the subsequent development of psychosis, particularly in individuals with an underlying genetic vulnerability (Zammit et al. 2002; Di Forti et al. 2015; McCutcheon et al. 2019). Δ^9^‐Tetrahydrocannabinol (THC), the principal psychoactive constituent of cannabis and the predominant cannabinoid in THC‐rich cannabis extracts, has been implicated in adverse neurodevelopmental outcomes following adolescent exposure, a critical period of synaptic maturation and circuit refinement (Rodrigues et al. 2024). Experimental studies using purified THC have shown that adolescent or adult THC exposure induces deficits in prepulse inhibition (PPI) (Tournier and Ginovart 2014; Renard et al. 2017), impairs social cognition (Renard et al. 2017), and accelerates dendritic pruning (Miller et al. 2019).

Accumulating evidence indicates that the long‐term consequences of adolescent cannabinoid exposure depend on the underlying neurodevelopmental context. In preclinical models relevant to schizophrenia, divergent outcomes have been reported. For example, adolescent THC exposure improved cognitive performance in adulthood in the prenatal methylazoxymethanol acetate (MAM) model, whereas it failed to rescue PPI deficits in a double‐hit model combining NMDAR antagonism with social isolation (Di Bartolomeo et al. 2023; Garcia‐Mompo et al. 2020). Likewise, previous studies have demonstrated interactions between adolescent cannabinoid exposure and schizophrenia‐relevant genetic or neurodevelopmental risk factors (Raver et al. 2013; Renard et al. 2017; Di Bartolomeo et al. 2023). Together, these findings indicate that the impact of adolescent cannabinoid exposure is strongly influenced by the biological context in which it occurs rather than representing a uniform consequence of cannabis exposure.

Despite these advances, important questions remain unresolved. Most previous studies have relied on environmental models or genetic models affecting pathways distinct from D‐serine metabolism, and the molecular mechanisms linking adolescent cannabinoid exposure to long‐term behavioral, neurochemical, and cellular alterations remain incompletely understood. To our knowledge, no previous study has investigated the long‐term consequences of adolescent exposure to a THC‐rich cannabis extract in the genetically defined SrrY269* model of D‐serine/NMDAR hypofunction. In particular, it remains unknown whether adolescent exposure to a THC‐rich extract modifies glutamatergic pathways associated with schizophrenia susceptibility, including the serine racemase/D‐serine system.

Beyond neuronal dysfunction, neuroimmune dysregulation has emerged as an important component of schizophrenia pathophysiology, with increasing evidence implicating glial cells, particularly microglia and astrocytes (Hartmann et al. 2024; Koskuvi et al. 2024; Laricchiuta et al. 2024). Postmortem studies have reported increased microglial density accompanied by reduced branching complexity in the prefrontal cortex, temporal cortex, and hippocampus of individuals with schizophrenia (Gober et al. 2022; Wierzba‐Bobrowicz et al. 2005), although other studies have found no significant differences (Hercher et al. 2014). Similarly, maternal immune activation models, including prenatal exposure to Poly I:C, induce persistent reductions in microglial ramification, supporting the contribution of altered microglial morphology to schizophrenia‐related phenotypes (Juckel et al. 2011).

Astrocytes have also emerged as active participants in schizophrenia pathophysiology rather than passive supporting cells. Postmortem studies reveal region‐specific alterations in astrocyte density and marker expression, whereas in vivo imaging studies demonstrate increased astrocytic reactivity in the anterior cingulate cortex and hippocampus that correlates with symptom severity (Kim et al. 2024; Bernstein et al. 2025). During neurodevelopment, disturbances in astrocyte differentiation and maturation affect glutamate and dopamine homeostasis, synaptogenesis, and redox balance, suggesting that genetic and environmental risk factors converge on glial dysfunction early in life (de Oliveira Figueiredo et al. 2022; Vellucci et al. 2025). In addition, astrocytes provide L‐serine that can support neuronal D‐serine synthesis, linking astrocytic metabolism to D‐serine availability, NMDAR signaling, and cognitive processes affected in schizophrenia (Wolosker 2011; de Oliveira Figueiredo et al. 2022).

Based on the interplay between cannabinoid signaling, D‐serine metabolism, and NMDAR function, we hypothesized that adolescent exposure to a THC‐rich cannabis extract would produce different long‐term outcomes in wild‐type and SrrY269* mice. Specifically, we predicted that the behavioral, neurochemical, and glial effects of adolescent exposure would depend on the genetically determined reduction in D‐serine availability and NMDAR function.

To test this hypothesis, we investigated the long‐term effects of adolescent exposure to a THC‐rich cannabis extract in the genetically defined SrrY269* model of D‐serine/NMDAR hypofunction. We examined adult behavioral and neurochemical outcomes together with microglial morphology in animals that completed the behavioral battery. Astrocytic morphology was evaluated separately in an independent behavior‐naïve cohort. Adolescent exposure to the THC‐rich cannabis extract prevented specific behavioral deficits in SrrY269* mice and was associated with genotype‐dependent alterations in microglial and astrocytic morphology. In contrast, the treatment produced adverse behavioral and astrocytic outcomes in wild‐type animals. These findings indicate that genetic background shapes the long‐term consequences of adolescent exposure to a THC‐rich cannabis extract.

## 2 Experimental Procedures

### 2.1 Animals

The animal procedures were approved by the ethics committee of the Health Sciences Center of the Federal University of Rio de Janeiro (CEUA, protocol no. 032/20). Male C57BL/6J mice (Central Animal Facility of the Health Sciences Center at the Federal University of Rio de Janeiro; RRID: IMSR_JAX:000664) and male mice carrying the serine racemase mutation (SrrY269*/SrrY269*) between 1 and 6 months old were used in the experiments. Animals were housed in cages with 2–5 mice per cage, with food and water *ad libitum*, under controlled temperature (22°C ± 1°C) under a 12‐h light/dark cycle (lights on from 07:00 to 19:00). All experiments were performed during the light phase.

The SrrY269* mice were obtained from the RIKEN BioResource Center (Japan; cat. no. RBRC‐GD000099). These animals were backcrossed to the C57BL/6J background for at least eight generations to minimize the contribution of background genetic variation unrelated to the SrrY269* mutation. SrrY269* mice exhibit approximately 90% lower D‐serine levels than Srr+/+ mice (Labrie, Fukumura, et al. 2009). Wild‐type littermates (Srr+/+) were generated from heterozygous (Srr+/−) breedings and were used as controls throughout the study.

Only male mice were included in this study to reduce biological variability and enable an initial characterization of the interaction between adolescent exposure to a THC‐rich cannabis extract and serine racemase deficiency. Potential sex‐dependent effects were beyond the scope of the present study and should be addressed in future investigations.

Animals were assigned to four experimental groups: Srr+/+ Ctrl, Srr+/+ THC‐rich, SrrY269* Ctrl, and SrrY269* THC‐rich. Sample sizes varied across experimental endpoints because of the longitudinal design and occasional animal loss during the study. The exact number of animals used in each experiment and each group is provided in the corresponding figure legends and Supplementary Statistical Tables: Appendix S1. A total of 99 male mice were used across all experimental procedures.

After completion of the behavioral battery, animals from the behaviorally tested cohort were allocated to two separate subsets: one for microglial morphometric analysis and the other for amino acid quantification by high‐performance liquid chromatography (HPLC). Thus, microglial morphology and amino acid levels were assessed in different animals belonging to the same behaviorally tested cohort. Astrocyte morphology was assessed in an independent behavior‐naïve cohort. Accordingly, the potential influence of prior behavioral testing on the microglial and neurochemical findings, as well as the use of different cohorts for the microglial and astrocytic analyses, was considered when interpreting these outcomes.

No formal random‐sequence generation procedure was used for treatment allocation. Animals were identified using a hydrogen peroxide (H_2_O_2_)‐based paw‐marking system and assigned to the vehicle or THC‐rich cannabis extract groups in a balanced manner within each cage and genotype. For example, in cages containing four animals of the same genotype, two animals were assigned to the vehicle group and two to the THC‐rich cannabis extract group. This procedure was used to maintain a balanced distribution of treatments across cages and genotypes.

### 2.2 THC‐Rich Cannabis Extract Treatment

The cannabis extract used in this study was rich in THC and contained 22.1 mg/mL THC, 1.0 mg/mL cannabidiol (CBD), and 0.9 mg/mL cannabinol (CBN). The THC‐rich cannabis extract was prepared using the Soxhlet extraction technique described by Carvalho et al. (2023). Briefly, dried cannabis flowers (Cinderela variety) were pulverized and heated at 120°C for 1 h. The plant material was then extracted using a Soxhlet apparatus with USP‐grade ethanol (Sharlau, Spain) at 80°C for 6 h. The solvent was removed by rotary evaporation under reduced pressure, and the resulting resin was diluted in absolute ethanol to obtain the THC, CBD, and CBN concentrations described above.

For treatment, the resulting ethanolic solution of the THC‐rich cannabis extract was formulated with Tween 20 (Sigma‐Aldrich, cat. no. P1379) and sterile saline at a ratio of 1:1:18 (ethanolic THC‐rich cannabis extract solution:Tween 20:saline). The vehicle was prepared using absolute ethanol, Tween 20, and sterile saline at the same ratio (1:1:18; ethanol:Tween 20:saline). Control animals received a volume of vehicle equivalent to that administered to the corresponding THC‐rich cannabis extract‐treated animals. Reported doses refer to the THC content of the extract rather than to the total extract mass. Although dose calculations were based on THC concentration, animals were also exposed to the CBD and CBN present in the extract. The corresponding estimated CBD and CBN exposure at each THC dose level is presented in Table S1.

The THC‐rich extract was administered orally at escalating doses between postnatal day (P) 31 and P50, corresponding to the murine adolescent period. The dosing schedule was as follows: 2.5 mg/kg (P31‐P33), 3.0 mg/kg (P34‐P36), 5.0 mg/kg (P37‐P39), and 10.0 mg/kg (P40‐P50). Dose selection was based on previous preclinical studies of adolescent cannabinoid exposure (Zamberletti and Rubino 2022; Torrens et al. 2022). Because pharmacokinetic equivalence between repeated oral administration of the THC‐rich extract in mice and human cannabis consumption cannot be assumed, no direct comparison to human patterns of cannabis use can be inferred.

Animals were allocated to vehicle or THC‐rich extract treatment in a balanced but nonrandom manner within each cage and genotype. The treatment period was selected to model murine adolescence, based on established neurodevelopmental equivalence (Andersen 2003; Di Bartolomeo et al. 2023).

### 2.3 Prepulse Inhibition (PPI)

Prepulse inhibition was assessed using a startle reflex measurement system (Panlab, Barcelona, Spain; catalog number not available). Mice were placed individually in an animal holder, and each session began with a 10‐min habituation period under continuous 65 dB white noise. This was followed by five startle pulses consisting of 120 dB white‐noise bursts lasting 40 ms.

The test session included pseudorandom presentations of pulse‐alone and prepulse‐pulse trials. Prepulses consisted of 20‐ms auditory stimuli at 75 or 85 dB, with prepulse‐to‐pulse onset intervals of 60 or 120 ms. The startle response was recorded for 200 ms following startle‐pulse onset. The intertrial interval varied between 12 and 30 s. Each trial type was presented 10 times, resulting in a total of 50 trials per session.

Prepulse inhibition was calculated using the formula:

$$\begin{array}{c} \%\mathrm{PPI}=100-\left[\left(\text{startle response during prepulse}‐\text{pulse trials}/\text{startle response during pulse}‐\text{alone trials}\right)\times 100\right]. \end{array}$$

PPI testing at P55, P90, and P120 was performed in the same cohort of animals. Animals with baseline startle responses lower than 1 were excluded from PPI analyses because insufficient acoustic startle reactivity prevents reliable PPI quantification. Exclusion details and exact group sizes for each time point are provided in the Supplementary Statistical Tables: Appendix S1.

### 2.4 Object Recognition Task

Spatial and nonspatial object recognition tasks were performed as previously described (Labrie, Fukumura, et al. 2009). Mice were allowed to explore a square arena (29.7 × 29.3 cm) containing four distinct objects during three 5‐min training sessions, separated by 5‐min inter‐session intervals.

Five minutes after the final training session, two objects were displaced diagonally, and mice were allowed to explore the arena for 5 min. Spatial memory performance was assessed by comparing the exploration time of displaced versus non‐displaced objects.

Twenty‐four hours after the spatial object recognition test, one familiar object was replaced by a novel object, and mice were allowed to explore the arena for 5 min. Nonspatial memory was evaluated using a discrimination index, calculated as the ratio of time spent exploring the novel object to the total object exploration time.

### 2.5 Open Field Test

Locomotor activity and exploratory behavior were evaluated using the open field test. Mice were placed in a 29.7 × 29.3 cm arena and allowed to freely explore for 5 min, while behavior was recorded by an overhead camera (Crawley and Goodwin 1980). Total distance traveled, time spent in the center, number of entries into the center, and crossings were quantified using Mouseglob tracking software.

All open field measures were obtained from the same behavioral session. In a small number of recordings, technical limitations related to tracking quality, including image contrast or illumination, prevented reliable extraction of specific parameters. These values were therefore treated as missing only for the affected endpoint. Missing data were based exclusively on technical quality‐control criteria and were not related to genotype, treatment, or behavioral outcome. Exact group sizes for each open field endpoint are provided in the Supplementary Statistical Tables: Appendix S1.

### 2.6 Morris Water Maze

Spatial learning and memory were assessed using the Morris water maze, as previously described (Labrie, Fukumura, et al. 2009). The maze consisted of a cylindrical pool (108.5 cm diameter) filled with opaque water maintained at 26°C ± 1°C. The pool was divided into four quadrants, and visual cues were positioned on surrounding walls.

On Day 1, mice were trained to locate a visible platform within 90 s. From Days 2 to 6, mice were trained to locate a submerged platform, with four trials per day, starting from different release points. Escape latency was averaged across trials each day. After 2 days of rest, memory retention was assessed in a probe trial without the platform, during which the time spent in the target quadrant was recorded for 60 s.

### 2.7 Immunohistochemistry

Mice were deeply anesthetized with isoflurane delivered by inhalation (3%–5% for induction and 1%–2% for maintenance) until loss of reflexes, prior to transcardial perfusion with saline for 5 min, followed by 4% paraformaldehyde for 15 min. Brains were post‐fixed in 4% paraformaldehyde for 48 h and stored in phosphate‐buffered saline (PBS) until sectioning. Coronal sections (50 μm) were obtained using a vibratome (Leica Biosystems, RRID: SCR_016495).

Sections were blocked in PBS containing 5% bovine serum albumin (BSA; Sigma‐Aldrich, cat. no. A9647) and 0.5% Triton X‐100 (Millipore, cat. no. 108603) for 2 h at room temperature and incubated overnight at 4°C with primary antibodies: rabbit anti‐Iba‐1 (1:300; FUJIFILM Wako, RRID: AB_3716536) and mouse anti‐GFAP (1:500; Thermo Fisher Scientific, RRID: AB_10598206). After washing, sections were incubated with Alexa Fluor 488 (Invitrogen; RRID: AB_2534093) and Alexa Fluor 555 (Thermo Fisher Scientific; RRID: AB_2535844) secondary antibodies (1:400) for 2 h, followed by DAPI (Thermo Fisher Scientific; RRID: AB_2629482) staining. Sections were mounted using Fluoromount (SouthernBiotech; RRID: AB_2628057) and stored at 4°C until imaging.

### 2.8 Microglial Morphometric Analysis

Microglial morphology was analyzed as previously described (Leser et al. 2025). Confocal Z‐stack images of the hippocampal CA1 region were acquired using a Leica SPE confocal microscope (RRID: SCR_002140) equipped with a 63× objective. Images were converted into maximum‐intensity projections and processed using ImageJ (RRID: SCR_003070). Skeletonization and morphometric analyses were performed using the AnalyzeSkeleton and FracLac plugins.

Image acquisition parameters, including laser power, detector gain, offset, pinhole aperture, and z‐step size, were kept constant across experimental groups within each immunostaining batch. Background subtraction was applied uniformly, and the same threshold values were used for all images within each batch to preserve Iba‐1‐positive processes while minimizing nonspecific background fluorescence.

Microglial cells were systematically evaluated within predefined sampling fields. All cells meeting the eligibility criteria within these fields were included in the analysis, without visual selection among eligible cells. Only microglial cells with a clearly identifiable soma and a complete arbor that could be reliably separated from neighboring Iba‐1‐positive cells were considered eligible. Cells were excluded only when overlapping processes or incomplete visualization of the arbor prevented reliable skeletonization. Punctate fluorescence in the surrounding field was not used as a criterion for cell or image exclusion. The same preprocessing and cell‐selection criteria were applied to all images, regardless of genotype or treatment.

Three to four animals per group were analyzed. Within the predefined sampling fields, all eligible microglial cells were quantified, resulting in 3–4 analyzed cells per animal. Measurements from individual cells were averaged within each animal, and the resulting animal means were used as the statistical unit for group‐level analyses. Thus, each biological replicate corresponded to one animal. Cell selection, image processing, and morphometric analyses were performed by investigators blinded to genotype and treatment.

### 2.9 Astrocyte Morphometric Analysis

Morphometric analyses of GFAP‐positive astrocytes were performed as previously described (Vitorino et al. 2022). Astrocytes from the CA1 region of the hippocampus were analyzed for cell body area, cell body perimeter, number of primary and secondary processes, primary process length, and primary process width using NeuronJ (RRID: SCR_002074) in ImageJ.

Astrocyte morphometric analyses were performed in an independent cohort that did not undergo behavioral testing before tissue collection. Between 6 and 7 animals per group were analyzed, with five astrocytes quantified per animal. Measurements obtained from individual cells were averaged within each animal, and animal means were used as the statistical unit for all group‐level analyses. Accordingly, each biological replicate corresponded to one animal. GFAP fluorescence intensity and GFAP‐positive area were also quantified using one value per animal.

Image acquisition parameters, including laser power, detector gain, offset, pinhole, and *z*‐step size, were kept constant across all experimental groups within each immunostaining batch to ensure direct quantitative comparisons among experimental groups. Background subtraction and thresholding were applied uniformly across all images. Only astrocytes with clearly identifiable somata and primary processes that were sufficiently isolated from neighboring GFAP‐positive structures were included. Cells with overlapping processes or incomplete arbors were excluded before morphometric analysis. Cell selection, image processing, and morphometric analyses were performed by investigators blinded to genotype and treatment.

### 2.10 High‐Performance Liquid Chromatography

Following behavioral testing, mice allocated to neurochemical analyses were deeply anesthetized with a ketamine/xylazine mixture (intraperitoneal injection; 100 mg/kg ketamine and 10 mg/kg xylazine) until loss of reflexes. Death was confirmed by cervical dislocation prior to tissue collection. The prefrontal cortex and hippocampus were then rapidly dissected and stored at −80°C. Amino acids were extracted using 5% trichloroacetic acid (Sigma‐Aldrich, cat. no. T6399) and quantified by HPLC as previously described (Hashimoto et al. 1992). Amino acid concentrations were normalized to total protein levels.

Because HPLC analyses were performed in animals that completed behavioral testing, these neurochemical measurements should be interpreted as adult amino acid levels measured after behavioral experience.

### 2.11 Data and Statistical Analysis

Sample sizes were determined based on previous studies (Guercio et al. 2014; Guercio et al. 2020). Statistical analyses were performed using GraphPad Prism 8.0 (RRID: SCR_002798).

Data were analyzed using two‐way analysis of variance (ANOVA), followed by Tukey's or Fisher's least significant difference (LSD) post hoc test, as appropriate. For most behavioral, neurochemical, and morphological analyses, the two ANOVA factors were genotype and treatment. For PPI analyses, the two‐way ANOVA factors were interstimulus interval (prepulse) and group (genotype and treatment combined into a single four‐level factor). For the Morris water maze acquisition phase, the two‐way ANOVA factors were training day and group. Fisher's LSD was applied only after a significant ANOVA and when a limited number of a priori comparisons were planned. This approach is consistent with previous studies employing prepulse inhibition (PPI) paradigms, in which Fisher's LSD has been used following significant ANOVA effects to evaluate predefined group comparisons (Sotoyama et al. 2011; Lipina et al. 2011; Saletti et al. 2017).

Data are presented as mean ± SD or mean ± SEM, as indicated in the corresponding figure legends. Statistical significance was set at *p* < 0.05. Data distribution was assessed using the Shapiro–Wilk test before statistical analysis. Parametric analyses were retained because ANOVA is generally robust to moderate departures from normality, particularly in the absence of extreme outliers. No formal outlier detection test was performed, and no data points were excluded based on outlier identification.

To avoid pseudoreplication, all glial morphometric analyses were reanalyzed using the animal as the experimental unit. Exact *F* values, degrees of freedom, exact *p* values, group sizes, statistical units, and exclusion details are provided in the Supplementary Statistical Tables: Appendix S1.

No formal inclusion or exclusion criteria were established beyond general health status and successful completion of the experimental procedures. No animals or data points were excluded based on behavioral or molecular outcomes, except for PPI analyses, in which animals with a baseline acoustic startle response lower than 1 were excluded because their startle reactivity was insufficient for reliable PPI quantification.

A small number of animals died during the course of the study, resulting in minor variations in group size across experiments. These animals were not replaced because of the longitudinal design, which included adolescent treatment (P31–P50) followed by behavioral and molecular assessments in adulthood. Because animal loss resulted in unbalanced longitudinal datasets, repeated‐measures analyses were not performed. Instead, PPI data obtained at P55, P90, and P120 were analyzed independently using two‐way ANOVA.

### 2.12 Blinding Procedures

All experiments were conducted under blinded conditions. One experimenter was responsible for animal treatment and group allocation, while a second experimenter, blinded to treatment and genotype, performed all behavioral testing and quantitative image analyses. Data generated from these analyses were subsequently returned to the first experimenter for statistical analysis. Thus, behavioral assessments and quantitative analyses were conducted without knowledge of experimental group assignments.

## 3 Results

### 3.1 Adolescent Exposure to a THC‐Rich Cannabis Extract Prevents Prepulse Inhibition Deficits in Adult *SrrY269* * Mice While Inducing Long‐Term PPI Impairment in Srr+/+ Mice

**FIGURE 1:**
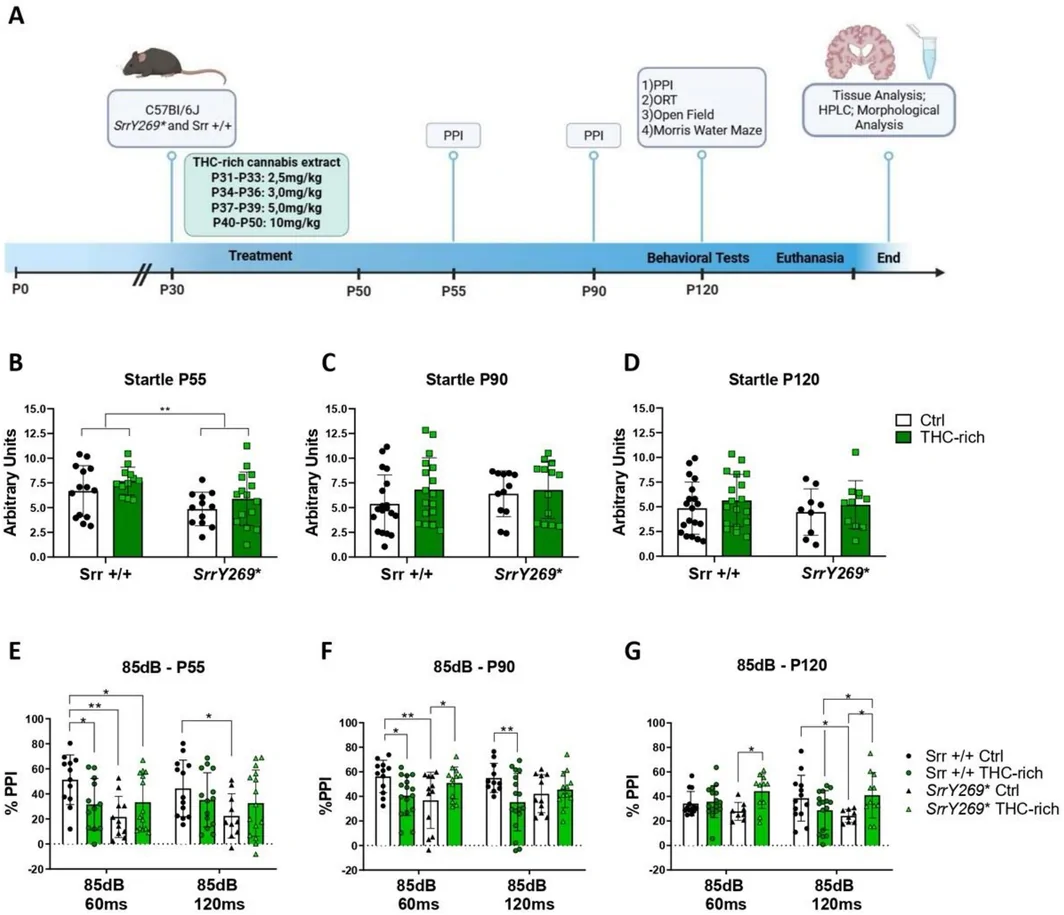
Effects of adolescent exposure to a THC‐rich cannabis extract on acoustic startle response and prepulse inhibition in Srr+/+ and SrrY269* mice. Experimental design showing adolescent exposure to a THC‐rich extract and subsequent behavioral and tissue analyses. Srr+/+ and SrrY269* mice were allocated in a balanced manner within each cage to receive vehicle (Srr+/+ Ctrl and SrrY269* Ctrl) or a THC‐rich extract (Srr+/+ THC‐rich and SrrY269* THC‐rich) during adolescence, from postnatal day (P) 31 to P50, using an escalating‐dose regimen. Prepulse inhibition (PPI) was assessed at P55, P90, and P120. In adulthood, mice also underwent the object recognition task (ORT), open field test, and Morris water maze. After completion of behavioral testing, animals from the behaviorally tested cohort were allocated to separate subsets for either amino acid quantification by HPLC or microglial morphometric analysis. Astrocyte morphometric analyses were performed in an independent behavior‐naïve cohort, as detailed in the Experimental Procedures (A). Acoustic startle response was assessed at P55 (B), P90 (C), and P120 (D). SrrY269* mice exhibited a reduced startle response at P55, whereas no significant differences were detected at P90 or P120. Prepulse inhibition was measured using an 85 dB prepulse with 60 ms and 120 ms interstimulus intervals at P55 (E), P90 (F), and P120 (G). At P55, both serine racemase deficiency and adolescent THC‐rich extract exposure reduced PPI, whereas no effect of THC‐rich extract was observed in SrrY269* mice at this time point. In adulthood, adolescent THC‐rich extract exposure prevented PPI deficits in SrrY269* mice at P90 and P120, restoring sensorimotor gating to levels comparable to Srr+/+ Ctrl mice, while inducing persistent PPI impairment in Srr+/+ mice at P90. The same cohort of animals was assessed longitudinally across all time points; group sizes varied due to animal loss during the experimental protocol. Data were analyzed using two‐way ANOVA followed by Fisher's LSD post hoc test after significant main effects or interactions. Data normality was assessed using the Shapiro–Wilk test. All statistical tests were two‐tailed, with significance set at *p* < 0.05. Data are presented as mean ± SEM. Exact *F* values, degrees of freedom, exact *p* values, group sizes, and exclusion details are provided in the Supplementary Statistical Tables: Appendix S1. Group sizes ranged from 8 to 19 animals per group depending on the analysis, reflecting occasional animal loss during the longitudinal protocol and technical exclusion from PPI analyses (baseline startle response < 1); see Supplementary Statistical Tables: Appendix S1 for the exact sample size used in each analysis. *p < 0.05, **p < 0.01. Ctrl, vehicle‐treated control group; HPLC, high‐performance liquid chromatography; ORT, object recognition task; PPI, prepulse inhibition; THC‐rich, treatment with the THC‐rich cannabis extract.

To investigate the long‐term effects of adolescent exposure to a THC‐rich cannabis extract, Srr+/+ and SrrY269* mice received either a THC‐rich cannabis extract or vehicle between P31 and P50. Behavioral assessments were performed at P55, P90, and P120. After completion of the behavioral battery, animals from this cohort were allocated to separate subsets for either amino acid quantification by HPLC or microglial morphometric analysis. Astrocyte morphology was assessed in an independent behavior‐naïve cohort (Figure 1A).

Consistent with previous reports, SrrY269* mice exhibit impaired prepulse inhibition (PPI), a widely used measure of sensorimotor gating (Labrie, Fukumura, et al. 2009; Guercio et al. 2014). We therefore investigated whether adolescent exposure to a THC‐rich extract modified this phenotype by assessing PPI throughout adulthood.

Five days after the final treatment (P55), mice were first evaluated for the acoustic startle response. A significant main effect of genotype was observed, with SrrY269* mice exhibiting lower startle amplitudes than Srr+/+ mice regardless of treatment (Figure 1B). No significant main effects of genotype or treatment, nor genotype × treatment interaction, were detected at P90 or P120 (Figure 1C,D), indicating that baseline acoustic reactivity normalized with age.

By contrast, prepulse inhibition followed a markedly different pattern. At P55, a significant main effect of experimental group was observed, whereas neither the main effect of interstimulus interval nor the interstimulus interval × group interaction reached statistical significance. Vehicle‐treated SrrY269* mice exhibited lower PPI than vehicle‐treated Srr+/+ control mice, consistent with impaired sensorimotor gating in early adulthood (Figure 1E). Post hoc analyses further indicated that adolescent exposure to the THC‐rich extract reduced PPI in Srr+/+ mice, while no significant treatment effect was detected in SrrY269* mice at this age.

A clear divergence between genotypes emerged by P90. Srr+/+ mice previously exposed to the THC‐rich extract continued to exhibit reduced PPI compared with Srr+/+ Ctrl mice, demonstrating persistent impairment of sensorimotor gating (Figure 1F). In contrast, SrrY269* mice exposed to the THC‐rich cannabis extract during adolescence exhibited significantly higher PPI than vehicle‐treated SrrY269* mice at the 60‐ms prepulse‐to‐pulse onset interval (Figure 1F).

The improvement in PPI observed in SrrY269* mice following adolescent THC‐rich extract exposure was maintained at P120, restoring sensorimotor gating to levels comparable to those of Srr+/+ Ctrl mice. In contrast, no significant reduction in PPI was observed in Srr+/+ mice at this time point (Figure 1G).

To determine whether these effects depended on prepulse intensity, additional analyses were performed using a 75 dB prepulse at P55, P90, and P120. No significant main effects of genotype or treatment, nor genotype × treatment interactions, were detected at any time point, indicating that the long‐term effects of adolescent exposure to the THC‐rich extract were selective to the 85 dB prepulse condition (Figure S1).

Overall, adolescent exposure to the THC‐rich extract produced strikingly different long‐term effects depending on genotype. Whereas treatment induced sensorimotor gating deficits in Srr+/+ mice that remained evident at P90 but were no longer statistically significant at P120, it prevented PPI impairment in SrrY269* mice at P90, with the improvement maintained at P120.

### 3.2 Adolescent Exposure to a THC‐Rich Cannabis Extract Prevents Spatial Memory Deficits in Adult *SrrY269* * Mice

Previous studies have shown that SrrY269* mice exhibit deficits in spatial object recognition, whereas nonspatial object recognition remains preserved (Labrie, Fukumura, et al. 2009). We therefore investigated whether adolescent exposure to a THC‐rich extract influences these cognitive alterations in adulthood using spatial and non‐spatial object recognition tasks together with the Morris water maze.

**FIGURE 2:**
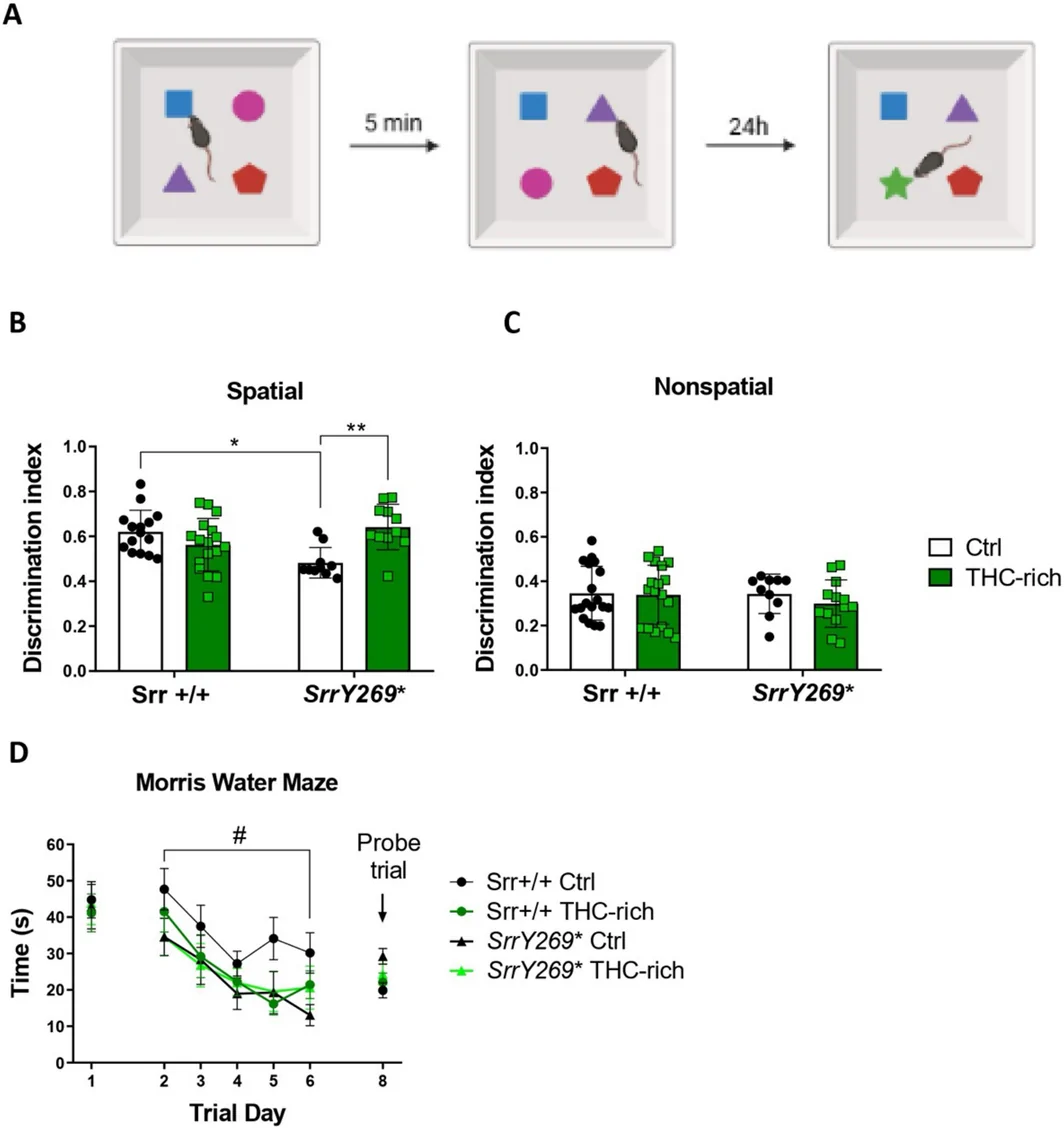
Effects of adolescent exposure to a THC‐rich cannabis extract on spatial and nonspatial cognitive performance in Srr+/+ and SrrY269* mice. Schematic representation of the object recognition task (ORT). Mice were subjected to the spatial ORT immediately after the final training session, followed by the nonspatial ORT 24 h later (A). Vehicle‐treated SrrY269* mice exhibited a reduced discrimination index in the spatial ORT compared with Srr+/+ Ctrl mice, whereas adolescent THC‐rich extract exposure prevented spatial memory deficits in adult SrrY269* mice. No effect of THC‐rich extract was observed in Srr+/+ mice (B). No significant differences were detected between genotypes or treatment groups in the non‐spatial ORT, indicating preserved non‐spatial memory (C). All groups exhibited progressive learning in the Morris water maze, as evidenced by the reduction in escape latency across training days (main effect of training day, #). No significant effects of genotype, treatment, or genotype × treatment interaction were detected during the visible platform or acquisition phases. In the probe trial, no significant effects of genotype or genotype × treatment interaction were observed, whereas a significant main effect of treatment was detected by two‐way ANOVA. However, Tukey's multiple comparisons test did not identify significant differences between any pair of groups (D). Data were analyzed using two‐way ANOVA followed by Tukey's post hoc test. Data are presented as mean ± SEM. *n* = 8–18 animals per group (see Supplementary Statistical Tables: Appendix S1 for the exact sample size used in each analysis). Exact *F* values, degrees of freedom, exact *p* values, and group sizes are provided in the Supplementary Statistical Tables: Appendix S1. *p < 0.05, **p < 0.01, #*p* < 0.001 (main effect of training day). Ctrl, vehicle‐treated control group; ORT, object recognition task; THC‐rich, treatment with the THC‐rich cannabis extract.

Mice first underwent three training sessions during which they explored four distinct objects for 5 min, separated by 5 min inter‐session intervals. Spatial object recognition was assessed immediately after the final training session by relocating two familiar objects (Figure 2A).

Vehicle‐treated SrrY269* mice exhibited a lower discrimination index than Srr+/+ Ctrl mice in the spatial object recognition task, consistent with the phenotype previously described for this model (Figure 2B; Labrie, Fukumura, et al. 2009). Adolescent exposure to the THC‐rich extract selectively improved performance in SrrY269* mice, with discrimination index values no longer differing from those of Srr+/+ Ctrl animals. In contrast, no differences were detected between Srr+/+ THC‐rich and Srr+/+ Ctrl mice, indicating that adolescent exposure to the THC‐rich extract did not affect spatial object recognition in wild‐type animals (Figure 2B).

Twenty‐four hours later, mice were evaluated in the non‐spatial object recognition task, in which one familiar object was replaced with a novel object. No significant main effects of genotype or treatment, nor genotype × treatment interaction, were detected (Figure 2C).

To determine whether these effects extended to other forms of hippocampus‐dependent memory, spatial learning and memory were further evaluated using the Morris water maze. All groups showed progressive reductions in escape latency across training days, reflected by a significant main effect of training day, indicating successful task acquisition (Figure 2D). No significant main effects of genotype or treatment, nor genotype × treatment interaction, were observed during training. During the probe trial, no significant main effect of genotype or genotype × treatment interaction was detected. Although a significant main effect of treatment was observed, Tukey's multiple comparisons test did not reveal significant differences between any pair of groups.

Overall, adolescent exposure to the THC‐rich extract selectively improved spatial object recognition in SrrY269* mice without affecting nonspatial object recognition. In the Morris water maze, no genotype‐dependent effects were observed, and although a significant main effect of treatment was detected during the probe trial, no significant pairwise differences between groups were identified by Tukey's multiple comparisons test. These findings indicate that the beneficial cognitive effects of adolescent THC‐rich extract exposure were restricted to a specific form of hippocampus‐dependent recognition memory rather than reflecting a generalized improvement in learning or memory.

### 3.3 Adolescent Exposure to a THC‐Rich Cannabis Extract Increases Time Spent in the Center in the Open Field Without Altering Locomotor Activity

**FIGURE 3:**
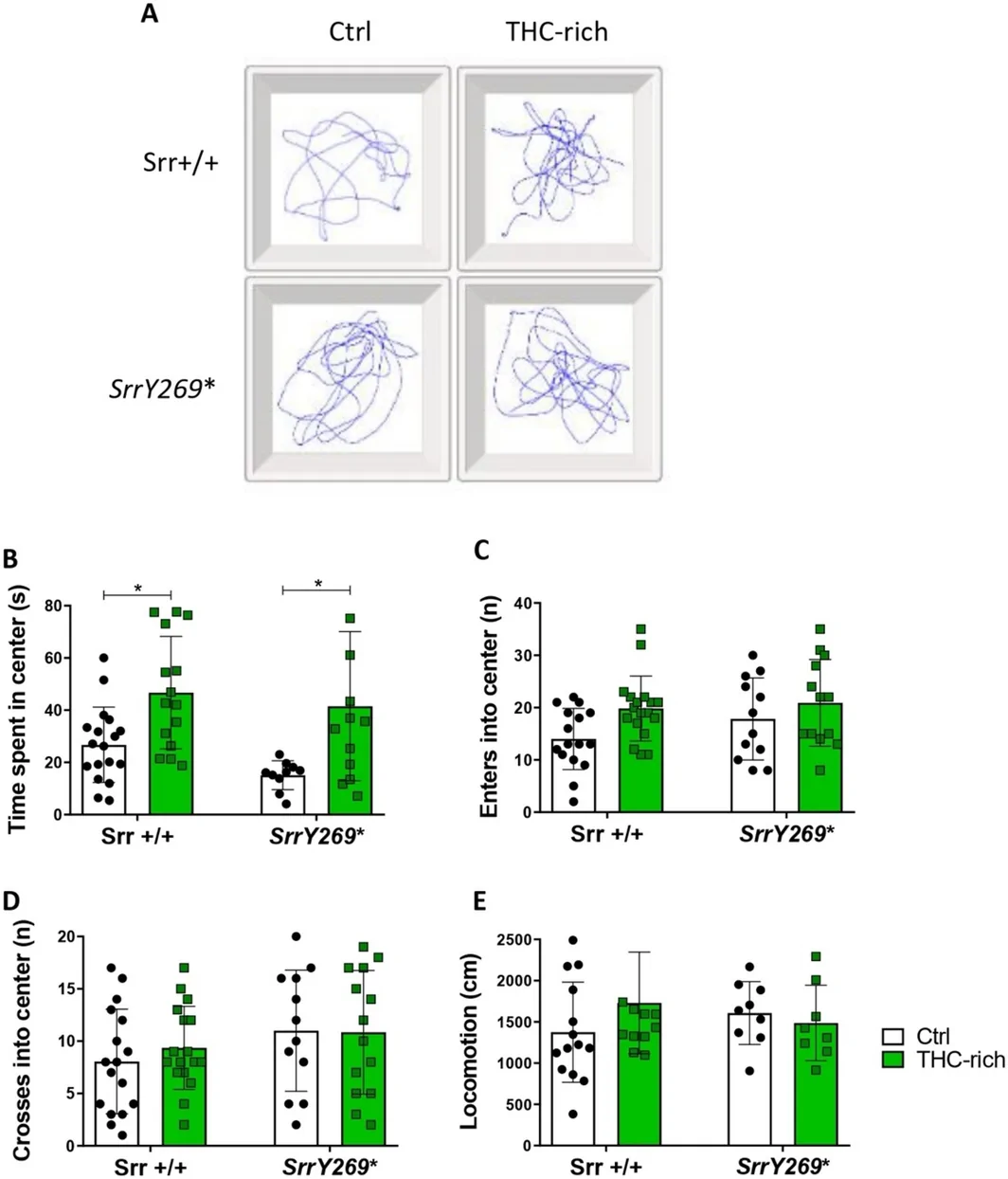
Effects of adolescent exposure to a THC‐rich cannabis extract on open field performance in adult Srr+/+ and SrrY269* mice. Representative tracking plots illustrating open field performance (A). Adolescent THC‐rich extract exposure increased time spent in the center of the open field in both Srr+/+ and SrrY269* mice (B). A significant main effect of treatment was observed for this parameter, whereas no significant main effect of genotype or genotype × treatment interaction was detected. No significant effects of genotype or treatment, and no genotype × treatment interaction, were detected for the number of entries into the center (C), the number of center crossings (D), or total distance traveled (E). Data were analyzed using two‐way ANOVA followed by Tukey's post hoc test. Data are presented as mean ± SEM. Group sizes ranged from 8 to 18 animals depending on the analysis (see Supplementary Statistical Table: Appendix S1 for exact group sizes). Exact *F* values, degrees of freedom, exact *p* values, and group sizes are provided in the Supplementary Statistical Tables: Appendix S1. *p < 0.05. Ctrl, vehicle‐treated control group; THC‐rich, treatment with the THC‐rich cannabis extract.

Open field behavior was evaluated to determine whether adolescent exposure to a THC‐rich extract produced long‐term behavioral alterations (Figure 3A).

Adolescent exposure to the THC‐rich extract significantly increased the time spent in the center of the arena in both Srr+/+ and SrrY269* mice compared with their respective Ctrl groups (Figure 3B). This effect was reflected by a significant main effect of treatment, whereas no significant main effect of genotype or genotype × treatment interaction was detected. Thus, the long‐term increase in center occupancy was independent of genotype.

In contrast, no significant main effects of genotype or treatment, nor genotype × treatment interaction, were observed for the number of entries into the center (Figure 3C), the number of center crossings (Figure 3D), or the total distance traveled (Figure 3E). These findings indicate that adolescent exposure to the THC‐rich extract did not alter baseline locomotor activity, suggesting that the increase in time spent in the center was not secondary to changes in general locomotion.

Overall, adolescent exposure to the THC‐rich extract increased the time spent in the center of the open field without affecting locomotor activity, indicating a selective effect on open field behavior.

### 3.4 Hippocampal Levels of Amino Acids Involved in NMDAR Signaling Are Altered by Serine Racemase Deficiency but Not by Adolescent THC‐Rich Extract Exposure

**FIGURE 4:**
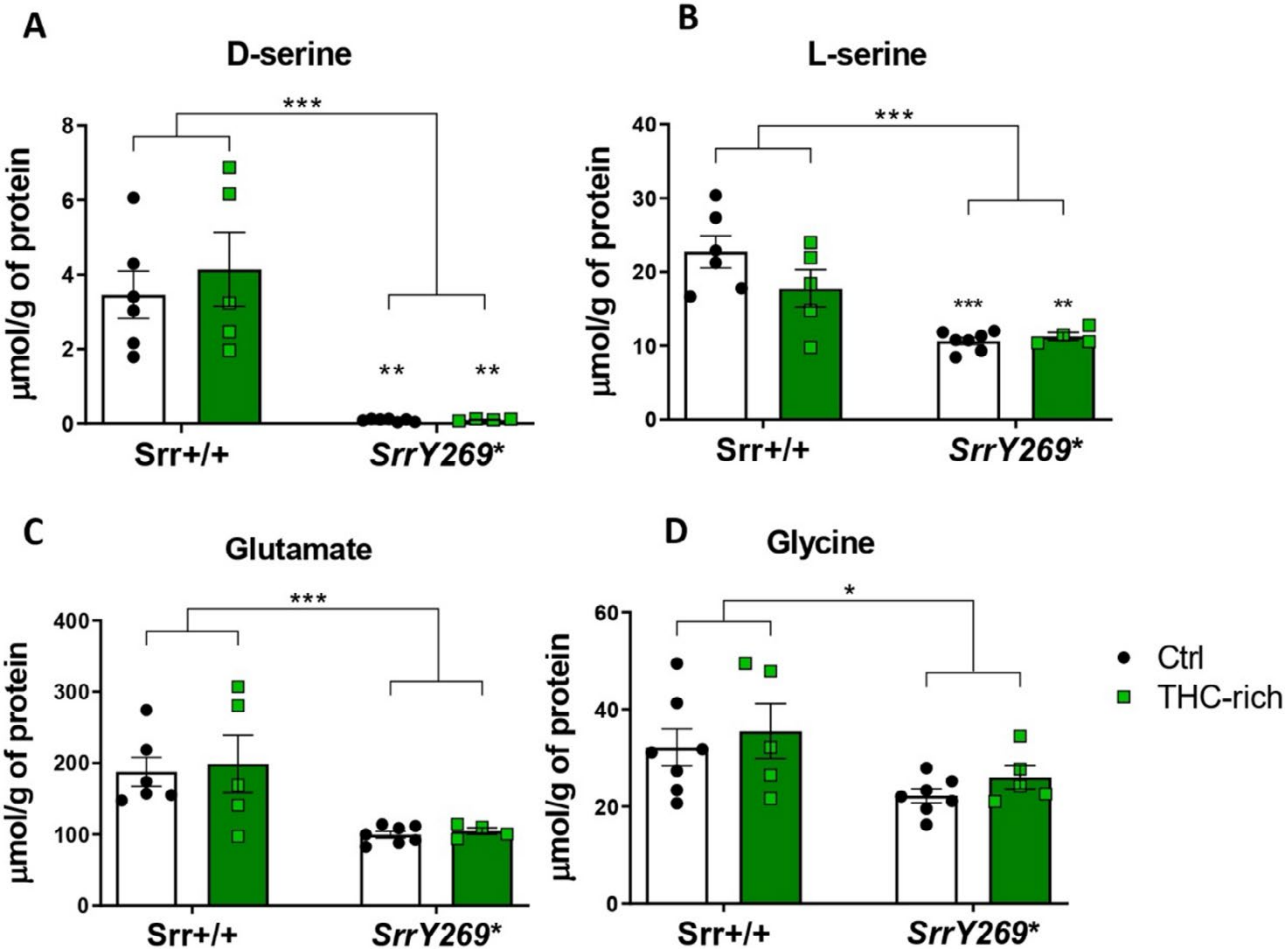
Effects of serine racemase deficiency and adolescent exposure to a THC‐rich cannabis extract on hippocampal amino acid levels in adult mice. Hippocampal amino acid concentrations were quantified by high‐performance liquid chromatography (HPLC). A significant main effect of genotype was observed for D‐serine (A), L‐serine (B), glutamate (C), and glycine (D), with lower concentrations in SrrY269* mice than in Srr+/+ mice. No significant main effect of treatment, and no genotype × treatment interaction, was detected for any amino acid analyzed. Data were analyzed using two‐way ANOVA followed by Tukey's post hoc test. Data are presented as mean ± SEM. Group sizes ranged from 4 to 7 animals depending on the analysis (see Supplementary Statistical Tables: Appendix S1 for exact group sizes). Exact *F* values, degrees of freedom, exact *p* values, and group sizes are provided in the Supplementary Statistical Tables: Appendix S1. *p < 0.05, **p < 0.01. Ctrl, vehicle‐treated control group; THC‐rich, treatment with the THC‐rich cannabis extract.

To determine whether adolescent exposure to a THC‐rich extract was associated with persistent neurochemical alterations, we quantified hippocampal levels of D‐serine, L‐serine, glutamate, and glycine in adult mice (Figure 4).

A significant main effect of genotype was observed for hippocampal D‐serine levels, with SrrY269* mice exhibiting markedly lower concentrations than Srr+/+ mice (Figure 4A). Neither treatment nor the genotype × treatment interaction significantly affected D‐serine levels.

A similar pattern was observed for hippocampal L‐serine, glutamate, and glycine. All three amino acids were present at lower concentrations in SrrY269* mice than in Srr+/+ mice, reflected by significant main effects of genotype (Figure 4B–D). No significant effects of treatment or genotype × treatment interaction were detected for any of these amino acids. Thus, adolescent exposure to the THC‐rich extract did not modify the hippocampal neurochemical alterations associated with serine racemase deficiency.

We next quantified amino acid levels in the prefrontal cortex (PFC) (Figure S2). In contrast to the hippocampus, a significant main effect of genotype was observed only for D‐serine, with lower concentrations in SrrY269* mice than in Srr+/+ mice (Figure S2A). No significant effects of treatment or genotype × treatment interaction were detected for D‐serine, L‐serine, glutamate, or glycine (Figure S2A–D).

Overall, serine racemase deficiency was associated with reduced hippocampal concentrations of D‐serine, L‐serine, glutamate, and glycine, whereas only D‐serine remained significantly reduced in the prefrontal cortex. Adolescent exposure to the THC‐rich extract did not alter amino acid levels in either brain region.

### 3.5 Adolescent Exposure to a THC‐Rich Cannabis Extract Restores Hippocampal Microglial Branching in Adult SrrY269* Mice

**FIGURE 5:**
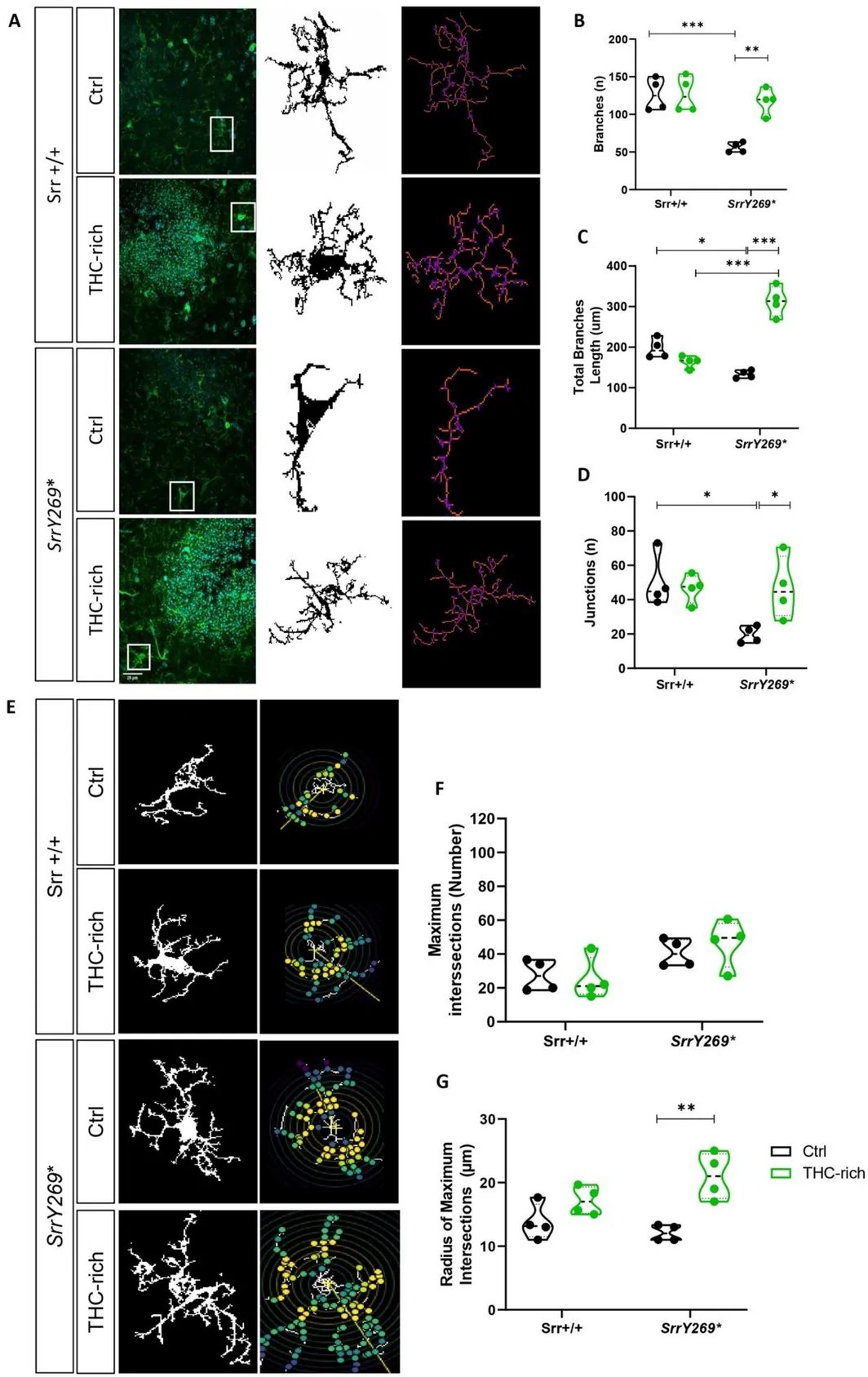
Effects of adolescent exposure to a THC‐rich cannabis extract on hippocampal microglial morphology in adult Srr+/+ and SrrY269* mice. Representative confocal images of hippocampal microglia immunolabeled with Iba‐1, together with the corresponding binary skeletons and reconstructed arborizations used for morphometric analysis (A). Quantitative analysis revealed significant genotype × treatment interactions for the number of branches (B), total branch length (C), and number of junctions (D). Vehicle‐treated SrrY269* mice exhibited reduced microglial complexity compared with Srr+/+ Ctrl mice. Adolescent THC‐rich extract exposure increased branch number, total branch length, and junction number in SrrY269* mice; branch number and junction number no longer differed from Srr+/+ Ctrl values, whereas total branch length was greater than in Srr+/+ Ctrl mice. No treatment effects were detected in Srr+/+ mice. Representative Sholl reconstructions of hippocampal microglia from each experimental group (E). Sholl analysis revealed a significant main effect of genotype for the maximum number of intersections (F) and a significant main effect of treatment for the radius of maximum intersections (G). Microglial morphology was assessed in a subset of animals that had previously completed the behavioral testing protocol. This subset was distinct from that used for amino acid quantification by HPLC. No significant differences were detected among groups in the maximum number of intersections. Post hoc analyses showed that SrrY269* THC‐rich mice exhibited an increased radius of maximum intersections compared with SrrY269* Ctrl mice. Four animals per group were analyzed, with 3–4 microglial cells quantified per animal in the CA1 region of the hippocampus. Measurements obtained from individual microglial cells were averaged for each animal, and animal means were used as the statistical unit for all analyses. Accordingly, each data point represents one animal. Data were analyzed using two‐way ANOVA followed by Tukey's post hoc test. For bar graphs, data are presented as mean ± SEM. For violin plots, data are shown as the distribution of individual animal means, with the central line representing the median. *p < 0.05, **p < 0.01, ***p < 0.001. Scale bar: 25 μm. Exact *F* values, degrees of freedom, exact *p* values, and group sizes are provided in the Supplementary Statistical Tables: Appendix S1. Ctrl, vehicle‐treated control group; Iba‐1, ionized calcium‐binding adapter molecule 1; THC‐rich, treatment with the THC‐rich cannabis extract.

We next examined hippocampal microglial morphology in a separate subset of animals from the behaviorally tested cohort (Figure 5). Significant genotype × treatment interactions were observed for the number of branches, total branch length, and number of junctions (Figure 5B–D). Vehicle‐treated SrrY269* mice exhibited fewer branches, shorter total branch length, and fewer junctions than Srr+/+ Ctrl mice, consistent with reduced microglial complexity. Adolescent exposure to the THC‐rich extract increased all three parameters in SrrY269* mice. Branch number and junction number no longer differed from those of Srr+/+ Ctrl animals, whereas total branch length was greater than in Srr+/+ Ctrl animals. No significant treatment effects were detected in Srr+/+ mice.

To further characterize these structural changes, we performed Sholl analysis in the hippocampus (Figure 5E–G). A significant main effect of genotype was observed for the maximum number of intersections (Figure 5F), although post hoc comparisons did not identify significant differences between individual groups. In contrast, a significant main effect of treatment was detected for the radius of maximum intersections (Figure 5G). Post hoc analyses showed that SrrY269* THC‐rich mice exhibited a greater radius of maximum intersections than SrrY269* Ctrl mice, indicating an increased spatial extent of microglial arborization following adolescent exposure to the THC‐rich extract.

We next evaluated microglial morphology in the prefrontal cortex (PFC) (Figure S3). In contrast to the hippocampus, significant genotype × treatment interactions were again observed for branch number, total branch length, and junction number (Figure S3B–D), but the direction of the effect differed. Adolescent exposure to the THC‐rich extract reduced all three parameters in SrrY269* mice. Sholl analysis revealed significant main effects of genotype and treatment for the maximum number of intersections (Figure S3F), whereas the radius of maximum intersections was also significantly affected by treatment (Figure S3G). Post hoc analyses showed that SrrY269* THC‐rich mice exhibited a greater radius of maximum intersections than SrrY269* Ctrl mice.

Overall, in a separate subset of the behaviorally tested cohort, adolescent exposure to the THC‐rich extract increased hippocampal microglial branch number, total branch length, and junction number in SrrY269* mice but reduced these parameters in the prefrontal cortex, indicating region‐dependent long‐term effects on microglial morphology.

### 3.6 Astrocyte Morphological Alterations in *SrrY269* * Mice Are Differentially Modulated by Adolescent Exposure to a THC‐Rich Cannabis Extract

**FIGURE 6:**
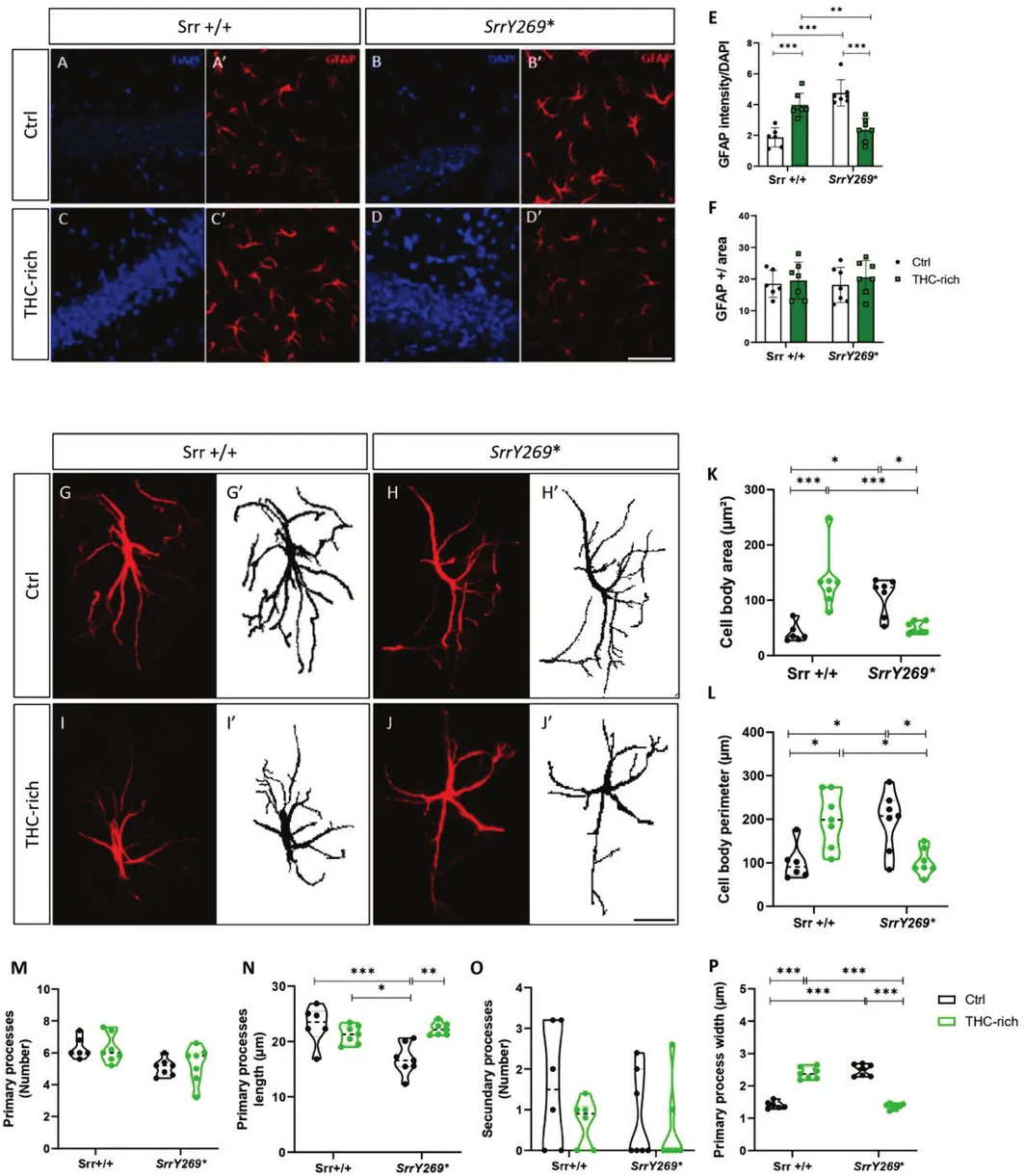
Effects of adolescent exposure to a THC‐rich cannabis extract on hippocampal astrocyte morphology in adult Srr+/+ and SrrY269* mice. Representative immunofluorescence images of hippocampal astrocytes immunolabeled for GFAP (red) with DAPI nuclear counterstaining (blue) are shown for Srr+/+ and SrrY269* mice treated with vehicle or THC‐rich extract during adolescence (A–D). A significant genotype × treatment interaction, together with a significant main effect of genotype, was observed for GFAP fluorescence intensity (E). SrrY269* Ctrl mice exhibited higher GFAP fluorescence intensity than Srr+/+ Ctrl mice. Adolescent THC‐rich extract exposure reduced GFAP fluorescence intensity in SrrY269* mice to levels no longer significantly different from Srr+/+ Ctrl mice. In contrast, Srr+/+ THC‐rich mice displayed higher GFAP fluorescence intensity than Srr+/+ Ctrl mice. No significant main effects of genotype or treatment, and no genotype × treatment interaction, were detected for GFAP‐positive area (F). Representative GFAP‐immunolabeled astrocytes (G–J) and the corresponding binary images used for morphometric analyses (G–'J') are shown. Significant genotype × treatment interactions were observed for soma area (K) and soma perimeter (L). SrrY269* Ctrl mice exhibited larger astrocytic somata than Srr+/+ Ctrl mice, whereas adolescent THC‐rich extract exposure largely restored both parameters in SrrY269* mice while increasing them in Srr+/+ mice. A significant main effect of genotype was observed for the number of primary processes (M), although post hoc comparisons did not identify significant differences between individual groups. Primary process length exhibited both a significant genotype × treatment interaction and a significant main effect of genotype (N). SrrY269* Ctrl mice displayed shorter primary processes than Srr+/+ Ctrl mice, whereas adolescent THC‐rich extract exposure largely restored primary process length in SrrY269* mice. No significant main effects or interactions were detected for the number of secondary processes (O). Primary process width showed a significant genotype × treatment interaction (P). Astrocyte morphology was assessed in an independent behavior‐naïve cohort that was distinct from the cohorts used for behavioral testing, microglial morphometry, and amino acid quantification. Adolescent THC‐rich extract exposure reduced primary process width in SrrY269* mice while increasing it in Srr+/+ *mice*. Between 6 and 7 animals per group were analyzed in the CA1 region of the hippocampus. Five astrocytes were quantified per animal. Measurements obtained from individual cells were averaged for each animal, and animal means were used as the statistical unit for all analyses. Accordingly, each data point represents one animal. Data were analyzed using two‐way ANOVA followed by Tukey's multiple comparisons test. Bar graphs are presented as mean ± SEM, whereas violin plots show the distribution of individual animal means, with the central line representing the median. *p < 0.05; **p < 0.01; ***p < 0.001. Scale bar: 50 μm. Exact *F* values, degrees of freedom, exact *p* values, and group sizes are provided in the Supplementary Statistical Tables: Appendix S1. Ctrl, vehicle‐treated control group; GFAP, glial fibrillary acidic protein; THC‐rich, treatment with the THC‐rich cannabis extract.

Postmortem studies have reported alterations in astrocyte density, morphology, and GFAP immunoreactivity in schizophrenia (Kolomeets et al. 2007). Using an independent behavior‐naïve cohort, we investigated whether astrocyte morphology was altered in SrrY269* mice and whether adolescent exposure to a THC‐rich cannabis extract modified these alterations in adulthood (Figure 6).

Representative GFAP‐immunolabeled astrocytes are shown in Figure 6A–D. GFAP fluorescence intensity exhibited both a significant genotype × treatment interaction and a significant main effect of genotype (Figure 6E). Vehicle‐treated SrrY269* mice displayed higher GFAP fluorescence intensity than Srr+/+ Ctrl mice. Adolescent exposure to the THC‐rich extract largely restored GFAP fluorescence intensity in SrrY269* mice, with values no longer differing from those of Srr+/+ Ctrl animals. In contrast, Srr+/+ THC‐rich mice exhibited higher GFAP fluorescence intensity than Srr+/+ Ctrl mice. No significant main effects of genotype or treatment, nor genotype × treatment interaction, were detected for GFAP‐positive area (Figure 6F), indicating that the observed differences reflected changes in GFAP immunoreactivity rather than astrocyte coverage.

We next performed a detailed morphometric analysis of GFAP‐positive astrocytes (Figure 6G–P). Significant genotype × treatment interactions were observed for both soma area (Figure 6K) and soma perimeter (Figure 6L). Vehicle‐treated SrrY269* mice exhibited larger astrocytic somata than Srr+/+ Ctrl mice. Adolescent exposure to the THC‐rich extract increased soma area and perimeter in Srr+/+ mice but reduced both parameters in SrrY269* mice, yielding values that were no longer significantly different from those of Srr+/+ Ctrl animals.

A similar pattern was observed for astrocytic processes. Although a significant main effect of genotype was detected for the number of primary processes (Figure 6M), post hoc comparisons did not identify significant differences between individual groups. Primary process length exhibited both a significant genotype × treatment interaction and a significant main effect of genotype (Figure 6N). Vehicle‐treated SrrY269* mice displayed shorter primary processes than Srr+/+ Ctrl mice, whereas adolescent exposure to the THC‐rich extract largely restored primary process length. No significant main effects or interactions were detected for the number of secondary processes (Figure 6O). In contrast, primary process width exhibited a significant genotype × treatment interaction (Figure 6P). Vehicle‐treated SrrY269* mice displayed thicker primary processes than Srr+/+ Ctrl mice. Adolescent exposure to the THC‐rich extract reduced primary process width in SrrY269* mice while increasing it in Srr+/+ mice.

Overall, in an independent behavior‐naïve cohort, SrrY269* mice exhibited marked alterations in hippocampal astrocyte morphology, characterized by increased GFAP immunoreactivity and remodeling of the soma and primary processes. Adolescent exposure to the THC‐rich cannabis extract attenuated several of these alterations in SrrY269* mice while promoting a distinct pattern of astrocytic remodeling in Srr+/+ mice, indicating genotype‐dependent astrocytic responses to treatment.

## 4 Discussion

One of the most compelling findings of this study is that adolescent exposure to a THC‐rich cannabis extract produced opposite outcomes depending on the underlying genetic background. Whereas adolescent THC‐rich cannabis extract exposure impaired sensorimotor gating and induced distinct glial alterations in Srr+/+ mice, it prevented PPI deficits, restored spatial recognition memory to levels comparable to those of Srr+/+ Ctrl mice, and attenuated hippocampal microglial and astrocytic alterations in SrrY269* mice. These contrasting responses suggest that the biological consequences of adolescent cannabinoid exposure are shaped by the functional state of glutamatergic signaling during neurodevelopment.

Prepulse inhibition (PPI) best illustrates this genotype‐dependent response. As a translational measure of sensorimotor gating, PPI is consistently disrupted in schizophrenia and reliably reproduced in SrrY269* mice (Labrie, Fukumura, et al. 2009; Guercio et al. 2020). The literature examining the effects of adolescent cannabinoid exposure on PPI remains inconsistent. In healthy rodents, repeated exposure to THC or synthetic cannabinoid agonists during adolescence generally impairs PPI in adulthood (Schneider and Koch 2003; Abela et al. 2019). In contrast, neurodevelopmental models relevant to schizophrenia have shown attenuation or even prevention of PPI deficits following adolescent cannabinoid exposure (Garcia‐Mompo et al. 2020; Lamanna‐Rama et al. 2024).

Our findings are more closely aligned with this latter scenario. Adolescent THC‐rich cannabis extract exposure impaired PPI in Srr+/+ mice but prevented the deficit observed in SrrY269* animals, and this improvement remained evident in adulthood. The opposite responses observed in wild‐type and mutant mice reinforce the view that the neurodevelopmental consequences of cannabinoid exposure cannot be interpreted independently of the underlying disease‐related phenotype. Unlike models based on prenatal environmental insults, such as methylazoxymethanol acetate (MAM) or Poly I:C exposure (Jones et al. 2011; Winship et al. 2019), SrrY269* mice allow cannabinoid responses to be examined in the context of a defined genetic alteration affecting D‐serine metabolism and NMDAR function. This distinction may be particularly relevant because it suggests that cannabinoid signaling interacts differently with neural circuits already compromised by glutamatergic dysfunction than with those developing under physiological conditions.

Several interconnected brain regions contribute to PPI, including the brainstem, hippocampus, amygdala, nucleus accumbens, and prefrontal cortex (Kohl et al. 2013), all of which exhibit abundant CB1 expression (Lorenzetti et al. 2016). Through activation of these receptors, chronic THC exposure can disrupt endocannabinoid signaling and alter neuronal activity and functional connectivity across these networks (Di Marzo et al. 2015; Rudroff et al. 2022). Exactly how these adaptations develop remains unknown. The present findings are nevertheless compatible with the idea that adolescent THC‐rich cannabis extract exposure induces enduring changes within these circuits. The direction of these adaptations appears to depend on the pre‐existing state of glutamatergic signaling, offering one possible framework for understanding why the same treatment produced opposite behavioral outcomes in Srr+/+ and SrrY269* mice.

The cognitive effects of adolescent THC‐rich cannabis extract exposure were notably selective. Improvement was confined to the spatial object recognition task, in which SrrY269* mice performed at levels comparable to those of Srr+/+ Ctrl animals, whereas performance in the nonspatial object recognition task and Morris water maze remained unchanged. This pattern argues against a generalized enhancement of hippocampal function and instead points to a selective improvement in cognitive processes that appear particularly vulnerable to NMDAR hypofunction.

The Morris water maze findings do not diminish the relevance of the improvement observed in the spatial object recognition task. Although both paradigms depend on hippocampal function, they place distinct demands on memory processing. Spatial object recognition relies on detecting changes in the spatial arrangement of familiar objects over a relatively short time scale, whereas the Morris water maze requires repeated training, procedural learning, and the gradual formation of a stable spatial representation. Evidence also indicates that the contribution of the hippocampus to object recognition memory depends on the specific features and demands of the task (Broadbent et al. 2010; Barker and Warburton 2011). These findings suggest that adolescent THC‐rich cannabis extract exposure selectively improved forms of hippocampus‐dependent recognition memory that are particularly vulnerable to reduced D‐serine availability rather than broadly enhancing cognitive performance.

Open field behavior followed a different pattern. Adolescent THC‐rich cannabis extract exposure increased the time spent in the center of the arena without altering locomotor activity, and this effect was observed in both genotypes. Because center occupancy reflects the interaction between exploratory behavior, emotional state, and environmental context, it should not be interpreted as a direct measure of anxiety. The absence of changes in total distance traveled, center entries, and center crossings further indicates that increased center occupancy was not secondary to altered locomotion. The functional significance of this finding therefore remains uncertain. Additional behavioral paradigms specifically designed to evaluate anxiety‐related responses will be necessary to clarify its biological relevance.

The neurochemical findings add another important dimension to the interpretation of these behavioral outcomes. As expected, SrrY269* mice exhibited markedly reduced D‐serine concentrations in both the hippocampus and prefrontal cortex, confirming the neurochemical phenotype previously described for this model (Labrie, Fukumura, et al. 2009). Hippocampal levels of L‐serine, glutamate, and glycine were also reduced, whereas only D‐serine remained significantly decreased in the prefrontal cortex. None of these alterations was modified by adolescent THC‐rich cannabis extract exposure. Nevertheless, behavioral recovery was still observed in SrrY269* mice.

This divergence between neurochemical and behavioral outcomes provides an important clue for interpreting the effects of treatment. If behavioral improvement depended primarily on restoring D‐serine availability, corresponding changes in tissue amino acid concentrations might also have been expected. Instead, behavioral improvement occurred while the neurochemical phenotype remained essentially unchanged. These observations suggest that the effects of adolescent THC‐rich cannabis extract exposure may involve enduring cellular or circuit‐level adaptations that are not captured by measurements of total tissue amino acid content. Microglial remodeling, assessed in a separate subset of the behaviorally tested cohort, provides a biologically plausible context for this dissociation. The complementary astrocytic alterations identified in an independent behavior‐naïve cohort further support the possibility that changes in glial morphology and glia–neuron interactions contribute to the genotype‐dependent effects observed in SrrY269* mice.

The hippocampal microglial phenotype illustrates one such adaptation. Under basal conditions, SrrY269* mice displayed a less ramified microglial morphology, characterized by fewer branches, shorter total branch length, and fewer junctions. Adolescent THC‐rich cannabis extract exposure increased all three parameters, bringing branch number and junction number to levels comparable to those of Srr+/+ Ctrl mice while producing a more pronounced increase in total branch length. Reduced microglial complexity has been reported in postmortem studies of schizophrenia and in experimental models of NMDAR hypofunction (Hercher et al. 2014; Gober et al. 2022). Our findings extend these observations by showing that this phenotype remains responsive to adolescent cannabinoid exposure even after treatment has ceased.

The response was not uniform across brain regions. Whereas hippocampal microglial branching recovered in SrrY269* mice, the prefrontal cortex exhibited a distinct pattern of structural remodeling following adolescent THC‐rich cannabis extract exposure. Regional heterogeneity is now recognized as a defining feature of microglial biology (Masuda et al. 2020). The present findings are consistent with the idea that local cellular and circuit‐specific environments shape glial responses to cannabinoid signaling.

Astrocytes exhibited a complementary pattern of morphological alterations. Vehicle‐treated SrrY269* mice showed increased GFAP immunoreactivity, enlarged somata, shorter primary processes, and increased primary process width. Several of these alterations were attenuated by adolescent THC‐rich cannabis extract exposure. In contrast, the same treatment produced a distinct astrocytic phenotype in Srr+/+ mice, including increased GFAP immunoreactivity, soma enlargement, and increased primary process width. These opposite responses further support the influence of genotype on the long‐term effects of adolescent cannabinoid exposure. Because astrocytic morphology was assessed in an independent behavior‐naïve cohort, these findings should be considered complementary to the microglial and behavioral outcomes rather than paired responses measured in the same animals.

This interpretation is biologically plausible considering the diverse roles of astrocytes in glutamatergic neurotransmission. In addition to supplying L‐serine for neuronal D‐serine synthesis, astrocytes regulate extracellular glutamate concentrations, provide metabolic support to neurons, and participate actively in synaptic plasticity (Wolosker et al. 1999; Haydon and Carmignoto 2006; Balu 2016; Bodner et al. 2020). Altered astrocyte morphology and GFAP immunoreactivity have also been reported in postmortem studies of schizophrenia (Kolomeets et al. 2007; Catts et al. 2014), reinforcing the translational relevance of the phenotype observed in SrrY269* mice.

Although microglial and astrocytic morphology was assessed in different cohorts, both analyses revealed structural remodeling associated with adolescent THC‐rich cannabis extract exposure in SrrY269* mice. These complementary findings represent a consistent biological response observed in this genotype. Microglial remodeling occurred despite the absence of detectable normalization of tissue amino acid concentrations in a separate subset of the same behaviorally tested cohort. Although the present data do not establish whether these findings are mechanistically linked, this dissociation suggests that glial remodeling may contribute to the biological response to adolescent cannabinoid exposure under conditions of NMDAR hypofunction. Future studies assessing microglial and astrocytic morphology, glial function, behavior, and local extracellular amino acid dynamics within an integrated experimental design will be necessary to clarify the relationships among these outcomes.

Some limitations of this study should be considered. The THC‐rich cannabis extract was administered orally, and alternative routes of exposure may produce distinct neurobiological and behavioral outcomes. Accordingly, direct translation of repeated oral administration in mice to specific patterns of human cannabis use should be approached with caution because of species differences in pharmacokinetics, metabolism, cannabinoid bioavailability, and routes of administration.

The cannabinoid composition of the extract also deserves consideration. Although THC was the predominant constituent, the extract contained measurable amounts of cannabidiol (CBD) and cannabinol (CBN), particularly at the highest dose. Consequently, the effects observed in SrrY269* mice cannot be attributed exclusively to THC. This point is especially relevant given the reported antipsychotic and neuroprotective properties of CBD in preclinical and clinical studies (Iseger and Bossong 2015; McGuire et al. 2018). Human cannabis exposure typically involves complex phytocannabinoid mixtures rather than isolated THC, making the use of a botanical extract translationally relevant. Nevertheless, the individual contributions of THC, CBD, and CBN were not experimentally dissociated and require further investigation.

The interpretation of the astrocyte data also warrants caution. Although GFAP immunolabeling is widely used to assess astrocytic reactivity and structural remodeling, it labels only a subset of astrocytic processes and does not fully capture the complexity of the entire astrocytic arbor (Sofroniew and Vinters 2010; Khakh and Sofroniew 2015). Accordingly, the present findings should be interpreted as reflecting remodeling of GFAP‐positive astrocytic structures rather than the complete astrocytic domain. Additionally, the biological origin and significance of the punctate fluorescence observed in the surrounding field could not be determined within the present experimental design. Therefore, this signal was not interpreted as either nonspecific background or a treatment‐associated biological phenotype.

An additional limitation concerns the experimental cohorts used for the neurochemical and glial analyses. After completion of the behavioral battery, animals were allocated to separate subsets for either microglial morphometric analysis or amino acid quantification by HPLC, whereas astrocyte morphology was evaluated in an independent behavior‐naïve cohort. Consequently, the microglial and neurochemical findings may reflect the combined influence of genotype, adolescent THC‐rich cannabis extract exposure, and subsequent behavioral experience, rather than the effects of adolescent exposure alone. Although all animals within the behaviorally tested cohort underwent the same procedures, genotype‐ or treatment‐dependent interactions with behavioral testing cannot be excluded. Moreover, because microglial and astrocytic morphology were assessed in different cohorts, these findings should be interpreted as complementary rather than as coordinated or paired responses within the same animals.

Two additional aspects should also be acknowledged. Only male mice were included in this study, and future work will be necessary to determine whether similar effects occur in females. Moreover, although the present findings reveal an association between adolescent cannabinoid exposure, glial remodeling, and behavioral outcomes, they do not establish the underlying cellular or molecular mechanisms. Future studies integrating functional analyses of glial activity with selective manipulation of cannabinoid signaling will be important to clarify the contributions of CB1 activation, glia–neuron interactions, developmental timing, and individual phytocannabinoids to the phenotype described here.

Taken together, the present findings show that the long‐term consequences of adolescent exposure to a THC‐rich cannabis extract depend strongly on the underlying neurobiological context. Whereas the same treatment impaired sensorimotor gating and induced distinct glial alterations in Srr+/+ mice, it prevented behavioral deficits and attenuated hippocampal glial alterations in SrrY269* mice. Rather than supporting a uniform effect of adolescent cannabinoid exposure, these findings highlight the importance of genetic vulnerability in shaping its neurodevelopmental consequences and suggest that glial remodeling may contribute to the long‐term biological response to cannabinoid exposure under conditions of NMDAR hypofunction.

## Author Contributions

**Igor F. Rangel:** conceptualization, methodology, data curation, investigation, validation, formal analysis, supervision, visualization, writing – original draft. **Melissa Chaves:** conceptualization, methodology, data curation, investigation, validation, formal analysis, supervision, writing – original draft, visualization. **Iohanna Pagnoncelli:** validation, formal analysis. **Isabelle Medeiros:** validation, formal analysis, visualization. **Felipe Pinheiro:** investigation. **Lorena Fortuna:** investigation. **Luiza Castello‐Branco:** visualization. **Ernesto Diaz Roch:** visualization. **Virgínia Martins Carvalho:** writing – review and editing, funding acquisition. **Flavia Regina Souza Lima:** funding acquisition, writing – review and editing. **Rogério Panizzutti:** conceptualization, methodology, data curation, validation, supervision, funding acquisition, resources, writing – review and editing. **Luciana Romão:** conceptualization, methodology, data curation, validation, supervision, funding acquisition, project administration, resources, writing – original draft, writing – review and editing.

## Funding

This study was supported by the Coordenação de Aperfeiçoamento de Pessoal de Nível Superior (CAPES), Conselho Nacional de Desenvolvimento Científico e Tecnológico (CNPq), Fundação Carlos Chagas Filho de Amparo à Pesquisa do Estado do Rio de Janeiro (FAPERJ), Instituto Nacional de Ciência e Tecnologia da Glia (INCT da Glia – iGLIA), and the Ministério da Saúde/CNPq (process no. 444682/2023‐6).

## Conflicts of Interest

The authors declare no conflicts of interest.

## Supporting information


**Figure S1:** Effects of adolescent exposure to a THC‐rich cannabis extract on prepulse inhibition using a 75 dB prepulse in Srr+/+ and SrrY269* mice.
**Figure S2:** Effects of serine racemase deficiency and adolescent exposure to a THC‐rich cannabis extract on prefrontal cortical amino acid levels in adult Srr+/+ and SrrY269* mice.
**Figure S3:** Effects of adolescent exposure to a THC‐rich cannabis extract on microglial morphology in the prefrontal cortex of adult Srr+/+ and SrrY269* mice.
**Table S1:** Estimated cannabinoid exposure during administration of the THC‐rich cannabis extract.

## Data Availability

The data that support the findings of this study are available from the corresponding author upon reasonable request.
