# Supplementary material for: Adolescent Exposure to a THC‐Rich Cannabis Extract Produces Genotype‐Dependent Effects on Cognition and Glial Morphology in Serine Racemase Mutant Mice
Source: J Neurochem. 2026 Sep 25;170(9):e70559. doi: 10.1111/jnc.70559 (PMC13613077; doi:10.1111/jnc.70559)
Supplement: Supplementary file 1 [file JNC-170-e70559-s001.pdf]

Adolescent exposure to a THC-rich cannabis extract produces genotype-dependent effects on cognition and glial morphology in serine racemase mutant mice

Igor F. Rangel[1], Melissa Chaves[1], Iohanna Pagnoncelli[1], Isabelle Medeiros[1], Felipe Pinheiro[1], Lorena Fortuna[1], Luiza Castello-Branco[1], Ernesto Diaz Roch[3], Virgínia Martins Carvalho[3], Flavia Regina Souza Lima[1], Rogério Panizzutti[2], Luciana Romão[1]

[1] Instituto de Ciências Biomédicas, Universidade Federal do Rio de Janeiro, Rio de Janeiro, RJ, Brasil

[2] Instituto de Psiquiatria, Universidade Federal do Rio de Janeiro, Rio de Janeiro, RJ, Brasil

[3] Faculdade de Farmácia, Universidade Federal do Rio de Janeiro, Rio de Janeiro, RJ, Brasil

Correspondence: Luciana Romão and Rogério Panizzutti, Instituto de Ciências Biomédicas and Instituto de Psiquiatria, Universidade Federal do Rio de Janeiro, 21941-902, Rio de Janeiro, RJ, Brasil.

Email: luromao@gmail.com; rpanizzutti@gmail.com

Supplementary Figures

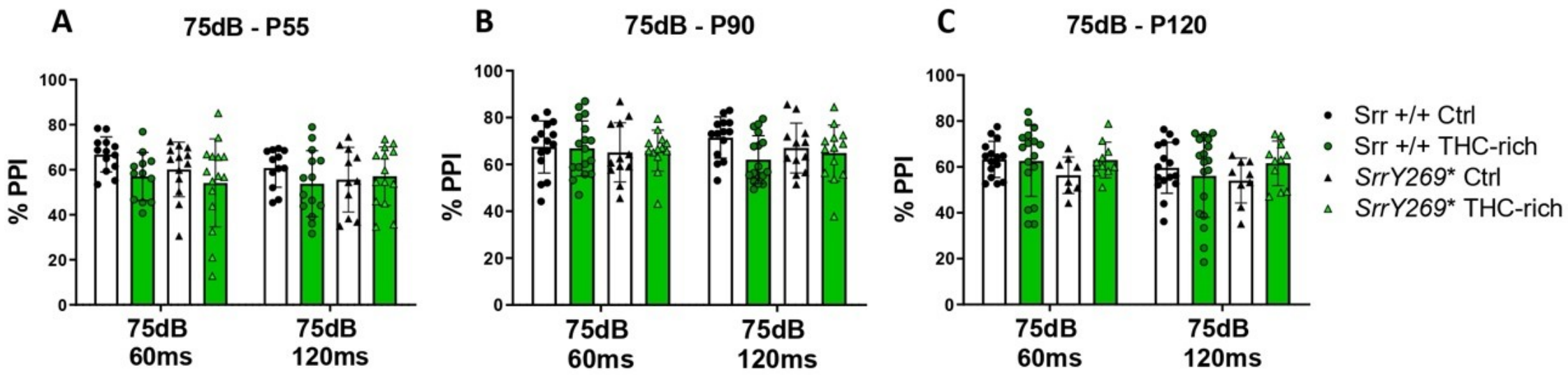

Supplementary Figure 1. Effects of adolescent exposure to a THC-rich cannabis extract on prepulse inhibition using a 75 dB prepulse in Srr+/+ and *SrrY269 mice.** Prepulse inhibition (PPI) was assessed using a 75 dB prepulse with randomized interstimulus intervals of 60 ms and 120 ms at P55 (A), P90 (B), and P120 (C). No significant main effects of interstimulus interval or group (genotype and treatment combined), and no interstimulus interval × group interactions, were detected at any time point. Data were analyzed using two-way ANOVA followed by Fisher's LSD post hoc test when appropriate. Data are presented as mean ± SEM. Group sizes ranged from 8-18 animals depending on the analysis (see Supplementary Statistical Tables for exact group sizes). Exact *F* values, degrees of freedom, exact *P* values, group sizes, and exclusion details are provided in the Supplementary Statistical Tables. PPI, prepulse inhibition; THC-rich, treatment with the THC-rich cannabis extract.

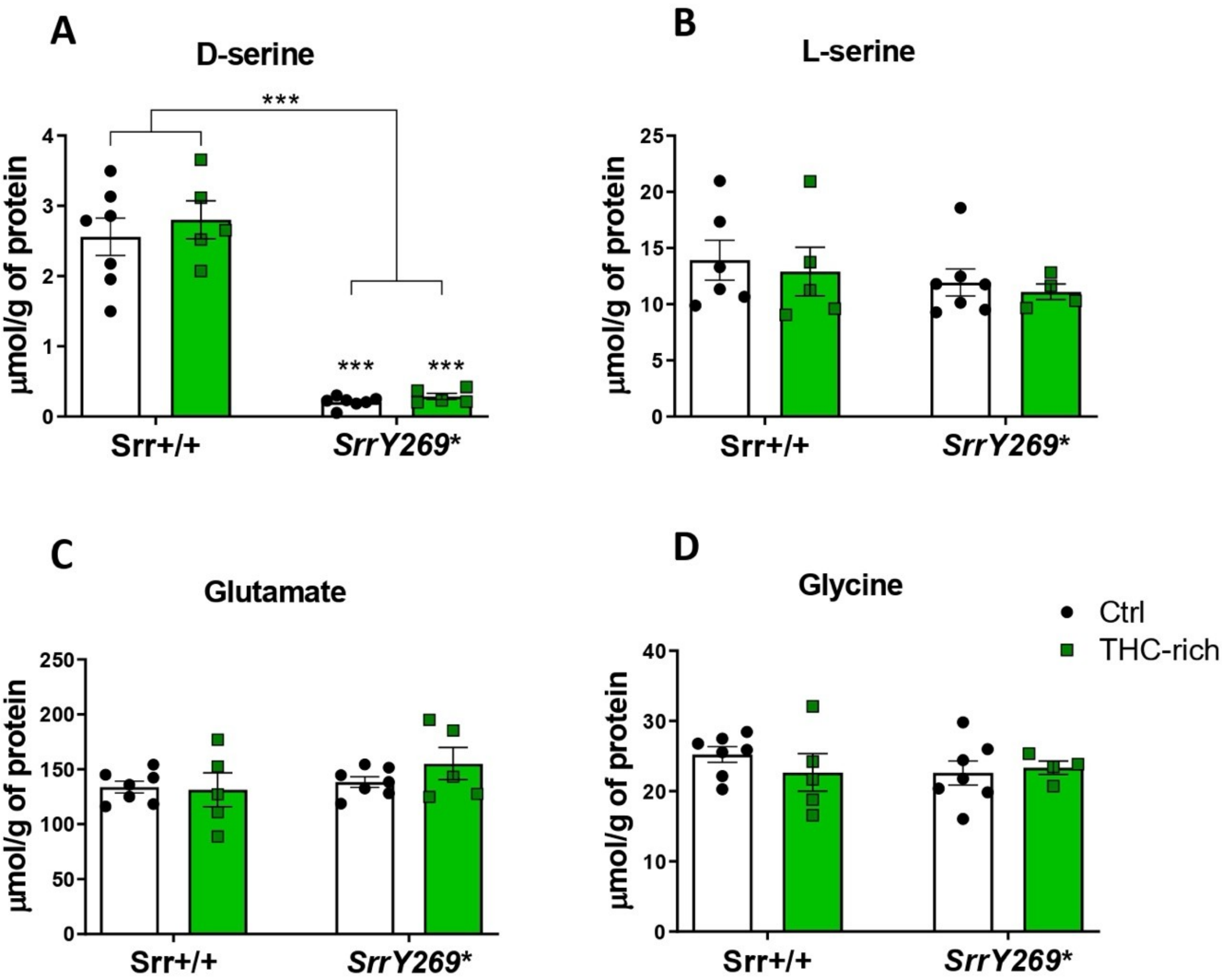

Supplementary Figure 2. Effects of serine racemase deficiency and adolescent exposure to a THC-rich cannabis extract on prefrontal cortical amino acid levels in adult Srr+/+ and *SrrY269 mice.** Amino acid concentrations in the prefrontal cortex (PFC) were quantified by high-performance liquid chromatography (HPLC). A significant main effect of genotype was observed only for D-serine (A), with lower concentrations in *SrrY269** mice than in Srr+/+ mice. No significant main effects of treatment, and no genotype × treatment interactions, were detected for D-serine, L-serine (B), glutamate (C), or glycine (D). Data were analyzed using two-way ANOVA followed by Tukey's multiple comparisons test. Data are presented as mean ± SEM. Group sizes ranged from 4-7 animals depending on the analysis (see Supplementary Statistical Tables for exact group sizes). ***$P < 0.001$. Exact *F* values, degrees of freedom, exact *P* values, and group sizes are provided in the Supplementary Statistical Tables. PFC, prefrontal cortex; HPLC, high-performance liquid chromatography; THC-rich, treatment with the THC-rich cannabis extract.

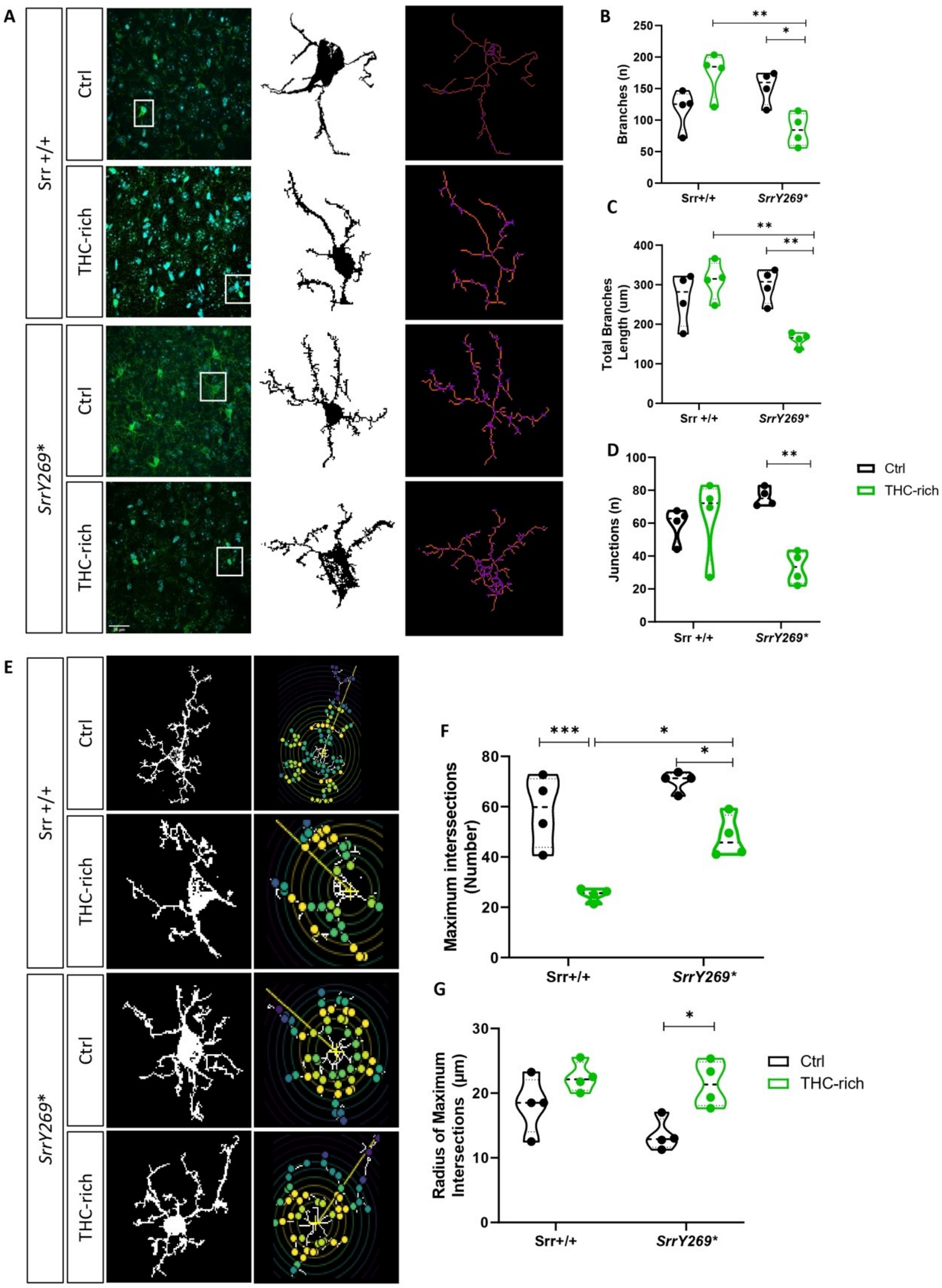

Supplementary Figure 3. Effects of adolescent exposure to a THC-rich cannabis extract on microglial morphology in the prefrontal cortex of adult Srr+/+ and *SrrY269 mice.** Representative confocal images of Iba-1-immunolabeled microglia in the prefrontal cortex (PFC), together with the corresponding binary skeletons and reconstructed arborizations used for

morphometric analysis (A). Significant genotype × treatment interactions were observed for the number of branches (B), total branch length (C), and number of junctions (D). Post hoc analyses showed that *SrrY269** THC-rich mice exhibited reduced microglial branching compared with *SrrY269** Ctrl mice, as evidenced by decreased branch number, total branch length, and junction number. No significant differences were detected between Srr+/+ Ctrl and Srr+/+ THC-rich mice. Representative Sholl reconstructions of prefrontal cortical microglia are shown in (E). A significant main effect of genotype and a significant main effect of treatment were observed for the maximum number of intersections (F), whereas no genotype × treatment interaction was detected. A significant main effect of treatment was observed for the radius of maximum intersections (G), whereas neither a significant main effect of genotype nor a genotype × treatment interaction was detected. Post hoc analyses showed that *SrrY269** THC-rich mice exhibited a greater radius of maximum intersections than *SrrY269** Ctrl mice. Four animals per group were analyzed in the PFC, with 3-4 microglial cells quantified per animal. Measurements obtained from individual cells were averaged for each animal, and animal means were used as the statistical unit for all analyses. Accordingly, each data point represents one animal. Data were analyzed using two-way ANOVA followed by Tukey's multiple comparisons test. Bar graphs are presented as mean ± SEM, whereas violin plots show the distribution of individual animal means, with the central line representing the median. Scale bar: 25 µm. **P < 0.01; ***P < 0.001. Exact *F* values, degrees of freedom, exact *P* values, and group sizes are provided in the Supplementary Statistical Tables. Iba-1, ionized calcium-binding adapter molecule 1; PFC, prefrontal cortex; Ctrl, control; THC-rich, treatment with the THC-rich cannabis extract.

THC dose (mg/kg)	Corresponding CBD exposure (mg/kg)	Corresponding CBN exposure (mg/kg)
2.5	0.11	0.10
3.0	0.14	0.12
5.0	0.23	0.20
10.0	0.45	0.41

Supplementary Table S1. Estimated cannabinoid exposure during administration of the THC-rich cannabis extract. THC doses represent the administered Δ⁹-tetrahydrocannabinol (THC) dose (mg/kg). The corresponding cannabidiol (CBD) and cannabinol (CBN) doses were estimated according to their relative concentrations in the THC-rich cannabis extract (22.1 mg/mL THC, 1.0 mg/mL CBD, and 0.9 mg/mL CBN). Values are expressed as mg/kg and rounded to two decimal places.

Supplementary Statistical Tables

Exact statistical results, group sizes, and exclusion details

Table Analyzed	Figure 1B- Startle P55

Normality test

Group	Shapiro-Wilk test p value	n
Srr+/+ Ctrl	0.1776	14
Srr+/+ THC-rich	0.6976	12
SrrY269* Ctrl	0.9823	12
SrrY269* THC-rich	0.998	15

Main statistical analysis

Analysis item	Statistic	p value
Statistical test	Two-way ANOVA	
Post hoc test	Fisher's LSD	
Interaction	F(1, 49) = 0.0005455	P = 0.9815
Genotype	F(1, 49) = 8.629	P = 0.0050
Treatment	F(1, 49) = 2.756	P = 0.1033

Sample size and exclusions

Figure / Analysis	Age	Group	Overall cohort	Same animals across time points	Final n analyzed	Technical exclusion?	Reason for n variation / exclusion
Figure 1B – Startle	P55	Srr+/+ Ctrl	Yes	Partially overlapping	14	No	Subset analyzed at this age
Figure 1B – Startle	P55	Srr+/+ THC-rich	Yes	Partially overlapping	12	No	Subset analyzed at this age
Figure 1B – Startle	P55	SrrY269* Ctrl	Yes	Partially overlapping	12	No	Subset analyzed at this age
Figure 1B – Startle	P55	SrrY269* THC-rich	Yes	Partially overlapping	15	No	Subset analyzed at this age

Table Analyzed	Figure 1C - Startle P90

Normality test

Group	Shapiro-Wilk test p value	n
Srr+/+ Ctrl	0.1697	19
Srr+/+ THC-rich	0.2599	17
SrrY269* Ctrl	0.0392	12
SrrY269* THC-rich	0.024	13

Main statistical analysis

Analysis item	Statistic	p value
Statistical test	Two-way ANOVA	
Post hoc test	Fisher's LSD	
Interaction	F(1, 57) = 0.4811	P = 0.4908
Genotype	F(1, 57) = 0.4227	P = 0.5182
Treatment	F(1, 57) = 1.470	P = 0.2303

Sample size and exclusions

Figure / Analysis	Age	Group	Overall cohort	Same animals across time points	Final n analyzed	Technical exclusion?	Reason for n variation / exclusion
Figure 1C – Startle	P90	Srr+/+ Ctrl	Yes	Partially overlapping	19	No	Partially overlapping subset across ages
Figure 1C – Startle	P90	Srr+/+ THC-rich	Yes	Partially overlapping	17	No	Partially overlapping subset across ages
Figure 1C – Startle	P90	SrrY269* Ctrl	Yes	Partially overlapping	12	No	Partially overlapping subset across ages
Figure 1C – Startle	P90	SrrY269* THC-rich	Yes	Partially overlapping	13	No	Partially overlapping subset across ages

Table Analyzed	Figure 1D - Startle P120

Normality test

Group	Shapiro-Wilk test p value	n
Srr+/+ Ctrl	0.1685	19
Srr+/+ THC-rich	0.342	18
SrrY269* Ctrl	0.5787	9
SrrY269* THC-rich	0.3696	11

Main statistical analysis

Analysis item	Result	
Statistical test	Two-way ANOVA	
Post hoc test	Fisher's LSD	
Interaction	F(1, 53) = 0.0007645	P = 0.9780
Genotype	F(1, 53) = 0.3393	P = 0.5627
Treatment	F(1, 53) = 1.152	P = 0.2880

Sample size and exclusions

Figure / Analysis	Age	Group	Overall cohort	Same animals across time points	Final n analyzed	Technical exclusion?	Reason for n variation / exclusion
Figure 1D – Startle	P120	Srr+/+ Ctrl	Yes	Partially overlapping	19	No	
Figure 1D – Startle	P120	Srr+/+ THC-rich	Yes	Partially overlapping	18	No	Final analyzable sample for this endpoint
Figure 1D – Startle	P120	SrrY269* Ctrl	Yes	Partially overlapping	9	No	Final analyzable sample for this endpoint
Figure 1D – Startle	P120	SrrY269* THC-rich	Yes	Partially overlapping	11	No	Final analyzable sample for this endpoint

Table Analyzed	Figure 1E - PPI P55 85 dB

Normality test

Group	Shapiro-Wilk test p value	n
Srr+/+ Ctrl 85 dB/60ms	0.7955	12
Srr+/+ THC-rich 85 dB/60ms	0.5006	12
Srr+/+ Ctrl 85 dB/120ms	0.1563	13
Srr+/+ THC-rich 85 dB/120ms	0.2064	14
SrrY269* Ctrl 85 dB/60ms	0.3454	11
SrrY269* THC-rich 85 dB/60ms	0.0122	14
SrrY269* Ctrl 85 dB/120ms	0.6521	10
SrrY269* THC-rich 85 dB/120ms	0.226	15

Main statistical analysis

Analysis item	Result	
Statistical test	Two-way ANOVA	
Post hoc test	Fisher's LSD	
Interstimulus interval × group	F(3, 93) = 0.2720	P = 0.8454
Prepulse-to-pulse onset interval	F(1, 93) = 0.03661	P = 0.8487
Group (genotype and treatment combined)	F(3, 93) = 5.647	P = 0.0013

Multiple Comparisons

Fisher's LSD	Mean Diff.	95.00% CI of diff.	Significant?	Summary	Individual P Value
Row 1					
Srr+/+ Ctrl vs. Srr+/+ THC-rich	19.71	2.305 to 37.12	Yes	*	0.0269
Srr+/+ Ctrl vs. SrrY269* Ctrl	29.75	11.95 to 47.54	Yes	**	0.0013
Srr+/+ Ctrl vs. SrrY269* THC-rich	18.04	1.268 to 34.81	Yes	*	0.0353
Srr+/+ THC-rich vs. SrrY269* Ctrl	10.04	-7.761 to 27.83	No	ns	0.2657
Srr+/+ THC-rich vs. SrrY269* THC-rich	-1.67	-18.44 to 15.10	No	ns	0.8437
SrrY269* Ctrl vs. SrrY269* THC-rich	-11.71	-28.88 to 5.472	No	ns	0.1793
Row 2					
Srr+/+ Ctrl vs. Srr+/+ THC-rich	9.163	-7.259 to 25.58	No	ns	0.2707
Srr+/+ Ctrl vs. SrrY269* Ctrl	21.9	3.969 to 39.84	Yes	*	0.0172
Srr+/+ Ctrl vs. SrrY269* THC-rich	11.75	-4.404 to 27.91	No	ns	0.152
Srr+/+ THC-rich vs. SrrY269* Ctrl	12.74	-4.913 to 30.39	No	ns	0.1552
Srr+/+ THC-rich vs. SrrY269* THC-rich	2.589	-13.25 to 18.43	No	ns	0.7463
SrrY269* Ctrl vs. SrrY269* THC-rich	-10.15	-27.56 to 7.255	No	ns	0.2498

Sample size and exclusions

Figure / Analysis	Age	Group	Overall cohort	Same animals across time points	Technical exclusion?	Reason for n variation / exclusion
Figure 1E – PPI P55 85 dB	P55	Srr+/+ Ctrl	Yes	Partially overlapping	baseline startle response < 1	Subset analyzed at this age
Figure 1E – PPI P55 85 dB	P55	Srr+/+ THC-rich	Yes	Partially overlapping	baseline startle response < 1	Subset analyzed at this age

Figure 1E – PPI P55 85 dB	P55	SrrY269* Ctrl	Yes	Partially overlapping	baseline startle response < 1	Subset analyzed at this age
Figure 1E – PPI P55 85 dB	P55	SrrY269* THC-rich	Yes	Partially overlapping	baseline startle response < 1	Subset analyzed at this age

Table Analyzed	Figure 1F - PPI P90 85 dB

Normality test

**Group**	**Shapiro-Wilk test p value**	**n**
Srr+/+ Ctrl 85 dB/60ms	0.4789	12
Srr+/+ THC-rich 85 dB/60ms	0.3146	16
Srr+/+ Ctrl 85 dB/120ms	0.2866	11
Srr+/+ THC-rich 85 dB/120ms	0.3116	17
SrrY269* Ctrl 85 dB/60ms	0.0303	12
SrrY269* THC-rich 85 dB/60ms	0.4013	12
SrrY269* Ctrl 85 dB/120ms	0.0944	11
SrrY269* THC-rich 85 dB/120ms	0.9929	12

Main statistical analysis

**Analysis item**	**Result**	
Statistical test	Two-way ANOVA	
Post hoc test	Fisher's LSD	
Interstimulus interval × group	F(3, 95) = 0.5245	P = 0.6665
Prepulse-to-pulse onset interval	F(1, 95) = 0.2303	P = 0.6324
Group (genotype and treatment combin	F(3, 95) = 5.702	P = 0.0012

Multiple Comparisons

**Fisher's LSD**	**Mean Diff.**	**95.00% CI of diff.**	**Significant?**	**Summary**	**Individual P Value**
Row 1					
Srr+/+ Ctrl vs. Srr+/+ THC-rich	15.59	2.444 to 28.73	Yes	*	0.0206
Srr+/+ Ctrl vs. SrrY269* Ctrl	19.42	5.373 to 33.47	Yes	**	0.0072
Srr+/+ Ctrl vs. SrrY269* THC-rich	5.32	-8.729 to 19.37	No	ns	0.454
Srr+/+ THC-rich vs. SrrY269* Ctrl	3.836	-9.305 to 16.98	No	ns	0.5636
Srr+/+ THC-rich vs. SrrY269* THC-rich	-10.27	-23.41 to 2.876	No	ns	0.1243
SrrY269* Ctrl vs. SrrY269* THC-rich	-14.1	-28.15 to -0.05312	Yes	*	0.0492
Row 2					
Srr+/+ Ctrl vs. Srr+/+ THC-rich	19.66	6.342 to 32.97	Yes	**	0.0042
Srr+/+ Ctrl vs. SrrY269* Ctrl	12.81	-1.859 to 27.49	No	ns	0.0862
Srr+/+ Ctrl vs. SrrY269* THC-rich	9.434	-4.930 to 23.80	No	ns	0.1954
Srr+/+ THC-rich vs. SrrY269* Ctrl	-6.844	-20.16 to 6.472	No	ns	0.3102
Srr+/+ THC-rich vs. SrrY269* THC-rich	-10.22	-23.20 to 2.751	No	ns	0.1211
SrrY269* Ctrl vs. SrrY269* THC-rich	-3.38	-17.74 to 10.98	No	ns	0.6415

Sample size and exclusions

Figure / Analysis	Age	Group	Overall cohort	Same animals across time points	Technical exclusion?	Reason for n variation / exclusion
Figure 1F – PPI P90 85 dB	P90	Srr+/+ Ctrl	Yes	Partially overlapping	baseline startle response < 1	Same overall cohort with incomplete repeated observations
Figure 1F – PPI P90 85 dB	P90	Srr+/+ THC-rich	Yes	Partially overlapping	baseline startle response < 1	Same overall cohort with incomplete repeated observations
Figure 1F – PPI P90 85 dB	P90	SrrY269* Ctrl	Yes	Partially overlapping	baseline startle response < 1	Same overall cohort with incomplete repeated observations
Figure 1F – PPI P90 85 dB	P90	SrrY269* THC-rich	Yes	Partially overlapping	baseline startle response < 1	Same overall cohort with incomplete repeated observations

Table Analyzed	Figure 1G - PPI P120 85 dB

Normality test

Group	Shapiro-Wilk test p value	n
Srr+/+ Ctrl 85 dB/60ms	0.0049	13
Srr+/+ THC-rich 85 dB/60ms	0.5886	16
Srr+/+ Ctrl 85 dB/120ms	0.9162	13
Srr+/+ THC-rich 85 dB/120ms	0.0492	16
SrrY269* Ctrl 85 dB/60ms	0.3479	9
SrrY269* THC-rich 85 dB/60ms	0.1302	11
SrrY269* Ctrl 85 dB/120ms	0.2637	8
SrrY269* THC-rich 85 dB/120ms	0.6243	11

Main statistical analysis

Analysis item	Result	
Statistical test	Two-way ANOVA	
Post hoc test	Fisher's LSD	
Interstimulus interval × group	F(3, 88) = 0.7831	P = 0.5065
Prepulse-to-pulse onset interval	F(1, 88) = 0.6478	P = 0.4231
Group (genotype and treatment com	F(3, 88) = 4.728	P = 0.0042

Multiple Comparisons

Fisher's LSD	Mean Diff.	95.00% CI of diff.	Significant?	Summary	Individual P Value
Row 1					
Srr+/+ Ctrl vs. Srr+/+ THC-rich	-1.559	-12.11 to 8.995	No	ns	0.7698
Srr+/+ Ctrl vs. SrrY269* Ctrl	6.391	-6.310 to 19.09	No	ns	0.3201
Srr+/+ Ctrl vs. SrrY269* THC-rich	-10.08	-21.66 to 1.502	No	ns	0.0872
Srr+/+ THC-rich vs. SrrY269* Ctrl	7.95	-4.289 to 20.19	No	ns	0.2001
Srr+/+ THC-rich vs. SrrY269* THC-ri	-8.519	-19.59 to 2.552	No	ns	0.1298
SrrY269* Ctrl vs. SrrY269* THC-rich	-16.47	-29.60 to -3.335	Yes	*	0.0146
Row 2					
Srr+/+ Ctrl vs. Srr+/+ THC-rich	9.789	-0.7649 to 20.34	No	ns	0.0687
Srr+/+ Ctrl vs. SrrY269* Ctrl	14.39	1.684 to 27.09	Yes	*	0.0269
Srr+/+ Ctrl vs. SrrY269* THC-rich	-2.476	-14.06 to 9.104	No	ns	0.672
Srr+/+ THC-rich vs. SrrY269* Ctrl	4.596	-7.643 to 16.84	No	ns	0.4575
Srr+/+ THC-rich vs. SrrY269* THC-ri	-12.27	-23.34 to -1.194	Yes	*	0.0303
SrrY269* Ctrl vs. SrrY269* THC-rich	-16.86	-30.00 to -3.728	Yes	*	0.0125

Sample size and exclusions

Figure / Analysis	Age	Group	Overall cohort	Same animals across time points	Technical exclusion?	Reason for n variation / exclusion
Figure 1G – PPI P120 85 dB	P120	Srr+/+ Ctrl	Yes	Partially overlapping	baseline startle response < 1	Same overall cohort with incomplete repeated observations
Figure 1G – PPI P120 85 dB	P120	Srr+/+ THC-rich	Yes	Partially overlapping	baseline startle response < 1	Same overall cohort with incomplete repeated observations
Figure 1G – PPI P120 85 dB	P120	SrrY269* Ctrl	Yes	Partially overlapping	baseline startle response < 1	Same overall cohort with incomplete repeated observations
Figure 1G – PPI P120 85 dB	P120	SrrY269* THC-rich	Yes	Partially overlapping	baseline startle response < 1	Same overall cohort with incomplete repeated observations

Table Analyzed	Figure 2B - Spatial object recognition task

Normality test

Group	Shapiro-Wilk test p value	n
Srr+/+ Ctrl	0.3576	15
Srr+/+ THC-rich	0.9195	17
SrrY269* Ctrl	0.0096	10
SrrY269* THC-rich	0.3486	11

Main statistical analysis

Analysis item	Result	P value
Statistical test	Two-way ANOVA	
Post hoc test	Tukey's multiple comparisons test	
Interaction	F(1, 49) = 14.91	P = 0.0003
Genotype	F(1, 49) = 1.095	P = 0.3004
Treatment	F(1, 49) = 3.201	P = 0.0798

Multiple Comparisons

Tukey's multiple comparisons test	Predicted (LS) mean diff.	95.00% CI of diff.	Significant?	Summary	Adjusted P Value
Srr+/+ Ctrl vs. Srr+/+ THC-rich	0.05841	-0.03603 to 0.1529	No	ns	0.3637
Srr+/+ Ctrl vs. SrrY269* Ctrl	0.1384	0.02952 to 0.2472	Yes	**	0.0075
Srr+/+ Ctrl vs. SrrY269* THC-rich	-0.02093	-0.1268 to 0.08490	No	ns	0.9524
Srr+/+ THC-rich vs. SrrY269* Ctrl	0.07995	-0.02630 to 0.1862	No	ns	0.2016
Srr+/+ THC-rich vs. SrrY269* THC-rich	-0.07935	-0.1825 to 0.02382	No	ns	0.1857
SrrY269 Ctrl vs. SrrY269* THC-rich*	-0.1593	-0.2758 to -0.04281	Yes	**	0.0036

Sample size and exclusions

Figure / Analysis	Age	Group	Overall cohort	Same animals across time points	Final n analyzed	Technical exclusion?	Reason for n variation / exclusion
Figure 2B – Spatial object recognition task	P120	Srr+/+ Ctrl	Yes	Yes (same cohort across related tasks)	15	No	Valid dataset for spatial object recognition analysis
Figure 2B – Spatial object recognition task	P120	Srr+/+ THC-rich	Yes	Yes (same cohort across related tasks)	17	No	Valid dataset for spatial object recognition analysis
Figure 2B – Spatial object recognition task	P120	SrrY269* Ctrl	Yes	Yes (same cohort across related tasks)	10	No	Valid dataset for spatial object recognition analysis
Figure 2B – Spatial object recognition task	P120	SrrY269* THC-rich	Yes	Yes (same cohort across related tasks)	11	No	Valid dataset for spatial object recognition analysis

Table Analyzed	Figure 2C - Non-Spatial object recognition task

Normality test

Group	Shapiro-Wilk test p value	n
Srr+/+ Ctrl	0.0459	18
Srr+/+ THC-rich	0.0768	18
SrrY269* Ctrl	0.0533	12
SrrY269* THC-rich	0.7332	13

Main statistical analysis

Analysis item	Result	P value
Statistical test	Two-way ANOVA	
Post hoc test	Tukey’s multiple comparisons test	
Interaction	F(1, 57) = 0.03385	P = 0.8547
Genotype	F(1, 57) = 1.204	P = 0.2771
Treatment	F(1, 57) = 0.1713	P = 0.6805

Multiple Comparisons

Tukey's multiple comparisons test	Predicted (LS) mean diff.	95.00% CI of diff.	Significant?	Summary	Adjusted P Value
Srr+/+ Ctrl vs. Srr+/+ THC-rich	0.007095	-0.09744 to 0.1116	No	ns	0.9979
Srr+/+ Ctrl vs. SrrY269* Ctrl	0.02819	-0.08869 to 0.1451	No	ns	0.9192
Srr+/+ Ctrl vs. SrrY269* THC-rich	0.04664	-0.06751 to 0.1608	No	ns	0.7022
Srr+/+ THC-rich vs. SrrY269* Ctrl	0.0211	-0.09578 to 0.1380	No	ns	0.9637
Srr+/+ THC-rich vs. SrrY269* THC-rich	0.03955	-0.07460 to 0.1537	No	ns	0.7959
SrrY269 Ctrl vs. SrrY269* THC-rich*	0.01845	-0.1071 to 0.1440	No	ns	0.9798

Sample size and exclusions

Figure / Analysis	Age	Group	Overall cohort	Same animals across time points	Final n analyzed	Technical exclusion?	Reason for n variation / exclusion
Figure 2C – Non-Spatial object recognition task	P120	Srr+/+ Ctrl	Yes	Yes (same cohort across related tasks)	18	No	Valid dataset for non-spatial object recognition analysis
Figure 2C – Non-Spatial object recognition task	P120	Srr+/+ THC-rich	Yes	Yes (same cohort across related tasks)	18	No	Valid dataset for non-spatial object recognition analysis
Figure 2C – Non-Spatial object recognition task	P120	SrrY269* Ctrl	Yes	Yes (same cohort across related tasks)	12	No	Valid dataset for non-spatial object recognition analysis
Figure 2C – Non-Spatial object recognition task	P120	SrrY269* THC-rich	Yes	Yes (same cohort across related tasks)	13	No	Valid dataset for non-spatial object recognition analysis
							Valid dataset for non-spatial object recognition analysis

Table Analyzed	Figure 2D - Morris water maze

Figure / Analysis	Phase	Age	Statistical test	Effect / Comparison	Statistic	p value
Fig 2D – Morris Water Maze	Visible platform (Day 1)	P120	Two-way ANOVA	Interaction	F(1, 57) = 0.2741	P = 0.6026
Fig 2D – Morris Water Maze	Visible platform (Day 1)	P120	Two-way ANOVA	Genotype	F(1, 57) = 0.2718	P = 0.6042
Fig 2D – Morris Water Maze	Visible platform (Day 1)	P120	Two-way ANOVA	Treatment	F(1, 57) = 0.002527	P = 0.9601
Fig 2D – Morris Water Maze	Hidden platform acquisition	P120	Two-way ANOVA	Interaction	F(12, 220) = 0.6152	P = 0.8283
Fig 2D – Morris Water Maze	Hidden platform acquisition	P120	Two-way ANOVA	Day	F(4, 220) = 17.41	P < 0.0001
Fig 2D – Morris Water Maze	Hidden platform acquisition	P120	Two-way ANOVA	Treatment	F(3, 55) = 1.576	P = 0.2055
Fig 2D – Morris Water Maze	Hidden platform acquisition	P120	Two-way ANOVA	Subject (individual variability)	F(55, 220) = 7.605	P < 0.0001
Fig 2D – Morris Water Maze	Probe trial	P120	Two-way ANOVA	Interaction	F(1, 52) = 1.510	P = 0.2246
Fig 2D – Morris Water Maze	Probe trial	P120	Two-way ANOVA	Genotype	F(1, 52) = 0.5905	P = 0.4457
Fig 2D – Morris Water Maze	Probe trial	P120	Two-way ANOVA	Treatment	F(1, 52) = 4.402	P = 0.0408

Normality test

Group	Shapiro-Wilk test - Visible platform (Day 1)	n
Srr+/+ Ctrl	0.6418	18
Srr+/+ THC-rich	0.3024	18
SrrY269* Ctrl	0.0424	12
SrrY269* THC-rich	0.5678	13

Normality test

Group	Shapiro-Wilk test - Probe trial	n
Srr+/+ Ctrl	0.2353	16
Srr+/+ THC-rich	0.0761	17
SrrY269* Ctrl	0.7098	10
SrrY269* THC-rich	0.5256	13

Figure / Analysis	Age	Group	Overall cohort	Same animals across time points	Final n analyzed	Technical exclusion?	Reason for n variation / exclusion
Figure 2D – Morris Water Maze (Visible platform)	P120	Srr+/+ Ctrl	Yes	Partially overlapping	18	No	Valid dataset for visible platform analysis
Figure 2D – Morris Water Maze (Visible platform)	P120	Srr+/+ THC-rich	Yes	Partially overlapping	18	No	Valid dataset for visible platform analysis
Figure 2D – Morris Water Maze (Visible platform)	P120	SrrY269* Ctrl	Yes	Partially overlapping	12	No	Valid dataset for visible platform analysis
Figure 2D – Morris Water Maze (Visible platform)	P120	SrrY269* THC-rich	Yes	Partially overlapping	13	No	Valid dataset for visible platform analysis

Figure / Analysis	Age	Group	Overall cohort	Same animals across time points	Final n analyzed	Technical exclusion?	Reason for n variation / exclusion
Figure 2D – Morris Water Maze (Probe trial)	P120	Srr+/+ Ctrl	Yes	Partially overlapping	16	No	Valid dataset for probe trial analysis
Figure 2D – Morris Water Maze (Probe trial)	P120	Srr+/+ THC-rich	Yes	Partially overlapping	17	No	Valid dataset for probe trial analysis
Figure 2D – Morris Water Maze (Probe trial)	P120	SrrY269* Ctrl	Yes	Partially overlapping	10	No	Valid dataset for probe trial analysis
Figure 2D – Morris Water Maze (Probe trial)	P120	SrrY269* THC-rich	Yes	Partially overlapping	13	No	Valid dataset for probe trial analysis

Figure / Analysis	Age	Group	Overall cohort	Same animals across time points	Final n analyzed	Technical exclusion?	Reason for n variation / exclusion
Figure 2D – Morris Water Maze (Hidden platform acquisition)	P120	Srr+/+ Ctrl	Yes	Partially overlapping	17	No	Endpoint-specific subset with complete acquisition data across training days
Figure 2D – Morris Water Maze (Hidden platform acquisition)	P120	Srr+/+ THC-rich	Yes	Partially overlapping	18	No	Endpoint-specific subset with complete acquisition data across training days
Figure 2D – Morris Water Maze (Hidden platform acquisition)	P120	SrrY269* Ctrl	Yes	Partially overlapping	11	No	Endpoint-specific subset with complete acquisition data across training days
Figure 2D – Morris Water Maze (Hidden platform acquisition)	P120	SrrY269* THC-rich	Yes	Partially overlapping	13	No	Endpoint-specific subset with complete acquisition data across training days

Probe Trial

Tukey's multiple comparisons test	Mean Diff.	95.00% CI of diff.	Significant?	Summary	Adjusted P Value
Srr+/+ Ctrl vs. Srr+/+ THC-rich	-8.242	-17.95 to 1.464	No	ns	0.1225
Srr+/+ Ctrl vs. SrrY269* Ctrl	-1.141	-9.527 to 7.246	No	ns	0.9837
Srr+/+ Ctrl vs. SrrY269* THC-rich	-3.294	-12.28 to 5.697	No	ns	0.7657
Srr+/+ THC-rich vs. SrrY269* Ctrl	7.101	-2.495 to 16.70	No	ns	0.2148
Srr+/+ THC-rich vs. SrrY269* THC-rich	4.948	-5.180 to 15.08	No	ns	0.5692
SrrY269* Ctrl vs. SrrY269* THC-rich	-2.153	-11.02 to 6.718	No	ns	0.9171

Visible platform (Day 1)

Tukey's multiple comparisons test	Mean Diff.	95.00% CI of diff.	Significant?	Summary	Adjusted P Value
Srr+/+ Ctrl vs. Srr+/+ THC-rich	2.708	-16.67 to 22.09	No	ns	0.9826
Srr+/+ Ctrl vs. SrrY269* Ctrl	5.979	-15.69 to 27.65	No	ns	0.8846
Srr+/+ Ctrl vs. SrrY269* THC-rich	2.696	-18.47 to 23.86	No	ns	0.9867
Srr+/+ THC-rich vs. SrrY269* Ctrl	3.271	-18.40 to 24.94	No	ns	0.9782
Srr+/+ THC-rich vs. SrrY269* THC-rich	-0.01282	-21.18 to 21.15	No	ns	>0.9999
SrrY269* Ctrl vs. SrrY269* THC-rich	-3.284	-26.56 to 19.99	No	ns	0.9821

Hidden platform acquisition

Dunnett's multiple comparisons test	Predicted (LS) mean diff.	95.00% CI of diff.	Significant?	Summary	Adjusted P Value
Srr+/+ Ctrl					
2 vs. 3	10.19	-1.305 to 21.69	No	ns	0.0982
2 vs. 4	20.46	8.960 to 31.95	Yes	****	<0.0001
2 vs. 5	13.57	2.077 to 25.07	Yes	*	0.0147
2 vs. 6	17.49	5.989 to 28.98	Yes	***	0.0009
Srr+/+ THC-rich					
2 vs. 3	13.18	2.008 to 24.35	Yes	*	0.0148
2 vs. 4	17.33	6.161 to 28.51	Yes	***	0.0007
2 vs. 5	23.06	11.88 to 34.23	Yes	****	<0.0001
2 vs. 6	18.53	7.356 to 29.70	Yes	***	0.0003
SrrY269* Ctrl					
2 vs. 3	6.273	-8.019 to 20.56	No	ns	0.6548
2 vs. 4	15.64	1.345 to 29.93	Yes	*	0.027
2 vs. 5	15.32	1.027 to 29.61	Yes	*	0.0314
2 vs. 6	21.52	7.231 to 35.81	Yes	**	0.0011
SrrY269* THC-rich					
2 vs. 3	7.788	-5.358 to 20.93	No	ns	0.396
2 vs. 4	12.6	-0.5502 to 25.74	No	ns	0.0647
2 vs. 5	15.06	1.911 to 28.20	Yes	*	0.0189
2 vs. 6	13.87	0.7191 to 27.01	Yes	*	0.0352

Table Analyzed	Figure 3B - Time spent in the center of the open field

Normality test

Group	Shapiro-Wilk test p value	n
Srr+/+ Ctrl	0.5043	18
Srr+/+ THC-rich	0.0973	15
SrrY269* Ctrl	0.3809	10
SrrY269* THC-rich	0.2167	13

Main statistical analysis

Analysis item	Result	P value
Statistical test	Two-way ANOVA	
Post hoc test	Tukey's multiple comparisons test	
Interaction	F(1, 52) = 0.3616	P = 0.5502
Genotype	F(1, 52) = 2.435	P = 0.1247
Treatment	F(1, 52) = 18.58	P < 0.0001

Multiple Comparisons

Tukey's multiple comparisons test	Predicted (LS) mean diff.	95.00% CI of diff.	Significant?	Summary	Adjusted P Value
Srr+/+ Ctrl vs. Srr+/+ THC-rich	-19.94	-38.19 to -1.701	Yes	*	0.027
Srr+/+ Ctrl vs. SrrY269* Ctrl	11.62	-8.956 to 32.20	No	ns	0.4454
Srr+/+ Ctrl vs. SrrY269* THC-rich	-14.79	-33.78 to 4.206	No	ns	0.1777
Srr+/+ THC-rich vs. SrrY269* Ctrl	31.57	10.27 to 52.87	Yes	**	0.0014
Srr+/+ THC-rich vs. SrrY269* THC-rich	5.157	-14.62 to 24.93	No	ns	0.8996
SrrY269 Ctrl vs. SrrY269* THC-rich*	-26.41	-48.36 to -4.462	Yes	*	0.0124

Sample size and exclusions

Figure / Analysis	Age	Group	Overall cohort	Same animals across time points	Final n analyzed	Technical exclus	Reason for n variation / exclusion
Figure 3B – Time spent in the center	P120	Srr+/+ Ctrl	Yes	Partially overlapping	18	No	Valid dataset for open field center-time analysis
Figure 3B – Time spent in the center	P120	Srr+/+ THC-rich	Yes	Partially overlapping	15	No	Valid dataset for open field center-time analysis
Figure 3B – Time spent in the center	P120	SrrY269* Ctrl	Yes	Partially overlapping	10	No	Valid dataset for open field center-time analysis
Figure 3B – Time spent in the center	P120	SrrY269* THC-rich	Yes	Partially overlapping	13	No	Valid dataset for open field center-time analysis

Table Analyzed	Figure 3C - Enters into center of the open field

Normality test

Group	Shapiro-Wilk test p value	n
Srr+/+ Ctrl	0.2543	17
Srr+/+ THC-rich	0.0513	18
SrrY269* Ctrl	0.313	12
SrrY269* THC-rich	0.6701	14

Main statistical analysis

Analysis item	Result	P value
Statistical test	Two-way ANOVA	
Post hoc test	Tukey’s multiple comparisons test	
Interaction	F(1, 57) = 0.8621	P = 0.3571
Genotype	F(1, 57) = 0.8154	P = 0.3703
Treatment	F(1, 57) = 3.895	P = 0.0533

Multiple Comparisons

Tukey's multiple comparisons test	Predicted (LS) mean diff.	95.00% CI of diff.	Significant?	Summary	Adjusted P Value
Srr+/+ Ctrl vs. Srr+/+ THC-rich	-5.422	-11.87 to 1.025	No	ns	0.1286
Srr+/+ Ctrl vs. SrrY269* Ctrl	-3.422	-10.61 to 3.765	No	ns	0.5918
Srr+/+ Ctrl vs. SrrY269* THC-rich	-5.374	-12.25 to 1.505	No	ns	0.1762
Srr+/+ THC-rich vs. SrrY269* Ctrl	2	-5.103 to 9.103	No	ns	0.8784
Srr+/+ THC-rich vs. SrrY269* THC-rich	0.04762	-6.744 to 6.840	No	ns	>0.9999
SrrY269 Ctrl vs. SrrY269* THC-rich*	-1.952	-9.451 to 5.546	No	ns	0.9009

Sample size and exclusions

Figure / Analysis	Age	Group	Overall cohort	Same animals across time points	Final n analyzed	Technical exclusion?	Reason for n variation / exclusion
Figure 3C – Enters into center	P120	Srr+/+ Ctrl	Yes	Partially overlapping	17	No	Valid dataset for open field center-enters into center analysis
Figure 3C – Enters into center	P120	Srr+/+ THC-rich	Yes	Partially overlapping	18	No	Valid dataset for open field center-enters into center analysis
Figure 3C – Enters into center	P120	SrrY269* Ctrl	Yes	Partially overlapping	12	No	Valid dataset for open field center-enters into center analysis
Figure 3C – Enters into center	P120	SrrY269* THC-rich	Yes	Partially overlapping	14	No	Valid dataset for open field center-enters into center analysis

Table Analyzed	Figure 3D - Crosses into center of the open field

Normality test

Group	Shapiro-Wilk test p value	n
Srr+/+ Ctrl	0.43	17
Srr+/+ THC-rich	0.7266	17
SrrY269* Ctrl	0.6926	12
SrrY269* THC-rich	0.2661	14

Main statistical analysis

Analysis item	Result	P value
Statistical test	Two-way ANOVA	
Post hoc test	Tukey’s multiple comparisons test	
Interaction	F(1, 56) = 0.2879	P = 0.5937
Genotype	F(1, 56) = 2.755	P = 0.1025
Treatment	F(1, 56) = 0.1848	P = 0.6689

Multiple Comparisons

Tukey's multiple comparisons test	Predicted (LS) mean diff.	95.00% CI of diff.	Significant?	Summary	Adjusted P Value
Srr+/+ Ctrl vs. Srr+/+ THC-rich	-1.294	-5.955 to 3.366	No	ns	0.8825
Srr+/+ Ctrl vs. SrrY269* Ctrl	-2.941	-8.064 to 2.182	No	ns	0.4325
Srr+/+ Ctrl vs. SrrY269* THC-rich	-2.798	-7.702 to 2.105	No	ns	0.4379
Srr+/+ THC-rich vs. SrrY269* Ctrl	-1.647	-6.770 to 3.476	No	ns	0.8296
Srr+/+ THC-rich vs. SrrY269* THC-rich	-1.504	-6.408 to 3.400	No	ns	0.8484
SrrY269 Ctrl vs. SrrY269* THC-rich*	0.1429	-5.202 to 5.488	No	ns	0.9999

Sample size and exclusions

Figure / Analysis	Age	Group	Overall cohort	Same animals across time points	Final n analyzed	Technical	Reason for n variation / exclusion
Figure 3D – Crosses into center	P120	Srr+/+ Ctrl	Yes	Partially overlapping	17	No	Valid dataset for open field center-crosses into center analysis
Figure 3D – Crosses into center	P120	Srr+/+ THC-rich	Yes	Partially overlapping	17	No	Valid dataset for open field center-crosses into center analysis
Figure 3D – Crosses into center	P120	SrrY269* Ctrl	Yes	Partially overlapping	12	No	Valid dataset for open field center-crosses into center analysis
Figure 3D – Crosses into center	P120	SrrY269* THC-rich	Yes	Partially overlapping	14	No	Valid dataset for open field center-crosses into center analysis

Table Analyzed	Figure 3E - Locomotion of the open field

Normality test

Group	Shapiro-Wilk test p value	n
Srr+/+ Ctrl	0.5169	14
Srr+/+ THC-rich	0.0109	13
SrrY269* Ctrl	0.9798	9
SrrY269* THC-rich	0.5399	8

Main statistical analysis

Analysis item	Result	P value
Statistical test	Two-way ANOVA	
Post hoc test	Tukey's multiple comparisons test	
Interaction	F(1, 40) = 1.961	P = 0.1691
Genotype	F(1, 40) = 0.001110	P = 0.9736
Treatment	F(1, 40) = 0.4784	P = 0.4932

Multiple Comparisons

Tukey's multiple comparisons test	Predicted (LS) mean diff.	95.00% CI of diff.	Significant?	Summary	Adjusted P Value
Srr+/+ Ctrl vs. Srr+/+ THC-rich	-355.1	-920.4 to 210.2	No	ns	0.3454
Srr+/+ Ctrl vs. SrrY269* Ctrl	-232.1	-859.1 to 395.0	No	ns	0.7548
Srr+/+ Ctrl vs. SrrY269* THC-rich	-111.8	-762.2 to 538.7	No	ns	0.9672
Srr+/+ THC-rich vs. SrrY269* Ctrl	123.1	-513.3 to 759.5	No	ns	0.9542
Srr+/+ THC-rich vs. SrrY269* THC-rich	243.4	-416.1 to 902.8	No	ns	0.7564
SrrY269 Ctrl vs. SrrY269* THC-rich*	120.3	-592.8 to 833.4	No	ns	0.9688

Sample size and exclusions

Figure / Analysis	Age	Group	Overall cohort	Same animals across time points	Final n analyzed	Technical exclusion?	Reason for n variation / exclusion
Figure 3E – Locomotion	P120	Srr+/+ Ctrl	Yes	Partially overlapping	14	No	Valid dataset for locomotor activity analysis
Figure 3E – Locomotion	P120	Srr+/+ THC-rich	Yes	Partially overlapping	13	No	Valid dataset for locomotor activity analysis
Figure 3E – Locomotion	P120	SrrY269* Ctrl	Yes	Partially overlapping	9	No	Valid dataset for locomotor activity analysis
Figure 3E – Locomotion	P120	SrrY269* THC-rich	Yes	Partially overlapping	8	No	Valid dataset for locomotor activity analysis

Table Analyzed	Figure 4A - D-serine Hippocampus

Normality test

Group	Shapiro-Wilk test p value	n
Srr+/+ Ctrl	0.6626	6
Srr+/+ THC-rich	0.2544	5
SrrY269* Ctrl	0.0996	7
SrrY269* THC-rich	0.776	4

Main statistical analysis

Analysis item	Result	P value
Statistical test	Two-way ANOVA	
Post hoc test	Tukey's multiple comparisons test	
Interaction	F(1, 18) = 0.3317	P = 0.5718
Genotype	F(1, 18) = 40.50	P < 0.0001
Treatment	F(1, 18) = 0.3567	P = 0.5578

Multiple Comparisons

Tukey's multiple comparisons test	Predicted (LS) mean diff.	95.00% CI of diff.	Significant?	Summary	Adjusted P Value
Srr+/+ Ctrl vs. Srr+/+ THC-rich	-0.6815	-2.963 to 1.600	No	ns	0.8327
Srr+/+ Ctrl vs. SrrY269* Ctrl	3.363	1.266 to 5.459	Yes	**	0.0013
Srr+/+ Ctrl vs. SrrY269* THC-rich	3.35	0.9181 to 5.782	Yes	**	0.0054
Srr+/+ THC-rich vs. SrrY269* Ctrl	4.044	1.838 to 6.250	Yes	***	0.0003
Srr+/+ THC-rich vs. SrrY269* THC-rich	4.032	1.504 to 6.559	Yes	**	0.0014
SrrY269 Ctrl vs. SrrY269* THC-rich*	-0.01241	-2.374 to 2.349	No	ns	>0.9999

Sample size and exclusions

Figure / Analysis	Age	Group	Overall cohort	Same animals across time p	Final n analyzed	Technical exclusion?	Reason for n variation / exclusion
Figure 4A – D-serine Hippocampus	P120	Srr+/+ Ctrl	Yes	Partially overlapping	6	No	Valid dataset for D-serine quantification
Figure 4A – D-serine Hippocampus	P120	Srr+/+ THC-rich	Yes	Partially overlapping	5	No	Valid dataset for D-serine quantification
Figure 4A – D-serine Hippocampus	P120	SrrY269* Ctrl	Yes	Partially overlapping	7	No	Valid dataset for D-serine quantification
Figure 4A – D-serine Hippocampus	P120	SrrY269* THC-rich	Yes	Partially overlapping	4	No	Valid dataset for D-serine quantification

Table Analyzed	Figure 4B - L-serine Hippocampus

Normality test

Group	Shapiro-Wilk test p value	n
Srr+/+ Ctrl	0.7122	6
Srr+/+ THC-rich	0.853	5
SrrY269* Ctrl	0.3667	7
SrrY269* THC-rich	0.4231	4

Main statistical analysis

Analysis item	Result	P value
Statistical test	Two-way ANOVA	
Post hoc test	Tukey’s multiple comparisons test	
Interaction	F(1, 18) = 2.595	P = 0.1246
Genotype	F(1, 18) = 28.44	P < 0.0001
Treatment	F(1, 18) = 1.536	P = 0.2312

Multiple Comparisons

Tukey's multiple comparisons test	Predicted (LS) mean diff.	95.00% CI of diff.	Significant?	Summary	Adjusted P Value
Srr+/+ Ctrl vs. Srr+/+ THC-rich	4.957	-1.874 to 11.79	No	ns	0.2068
Srr+/+ Ctrl vs. SrrY269* Ctrl	12.08	5.801 to 18.35	Yes	***	0.0002
Srr+/+ Ctrl vs. SrrY269* THC-rich	11.43	4.149 to 18.71	Yes	**	0.0017
Srr+/+ THC-rich vs. SrrY269* Ctrl	7.12	0.5150 to 13.72	Yes	*	0.0321
Srr+/+ THC-rich vs. SrrY269* THC-rich	6.473	-1.093 to 14.04	No	ns	0.1093
SrrY269 Ctrl vs. SrrY269* THC-rich*	-0.6463	-7.716 to 6.424	No	ns	0.9937

Sample size and exclusions

Figure / Analysis	Age	Group	Overall cohort	Same animals across time points	Final n analyzed	Technical exclusion?	Reason for n variation / exclusion
Figure 4B – L-serine Hippocampus	P120	Srr+/+ Ctrl	Yes	Partially overlapping	6	No	Valid dataset for L-serine quantification
Figure 4B – L-serine Hippocampus	P120	Srr+/+ THC-rich	Yes	Partially overlapping	5	No	Valid dataset for L-serine quantification
Figure 4B – L-serine Hippocampus	P120	SrrY269* Ctrl	Yes	Partially overlapping	7	No	Valid dataset for L-serine quantification
Figure 4B – L-serine Hippocampus	P120	SrrY269* THC-rich	Yes	Partially overlapping	4	No	Valid dataset for L-serine quantification

Table Analyzed	Figure 4C - Glutamate Hippocampus

Normality test

Group	Shapiro-Wilk test p value	n
Srr+/+ Ctrl	0.1055	6
Srr+/+ THC-rich	0.4684	5
SrrY269* Ctrl	0.5063	7
SrrY269* THC-rich	0.7298	4

Main statistical analysis

Analysis item	Result	P value
Statistical test	Two-way ANOVA	
Post hoc test	Tukey's multiple comparisons test	
Interaction	$F(1, 18) = 0.01917$	$P = 0.8914$
Genotype	$F(1, 18) = 17.09$	$P = 0.0006$
Treatment	$F(1, 18) = 0.1332$	$P = 0.7194$

Multiple Comparisons

Tukey's multiple comparisons test	Predicted (LS) mean diff.	95.00% CI of diff.	Significant?	Summary	Adjusted P Value
Srr+/+ Ctrl vs. Srr+/+ THC-rich	-11.14	-98.04 to 75.76	No	ns	0.9832
Srr+/+ Ctrl vs. SrrY269* Ctrl	88.41	8.565 to 168.3	Yes	*	0.027
Srr+/+ Ctrl vs. SrrY269* THC-rich	83.4	-9.241 to 176.0	No	ns	0.0864
Srr+/+ THC-rich vs. SrrY269* Ctrl	99.55	15.51 to 183.6	Yes	*	0.0171
Srr+/+ THC-rich vs. SrrY269* THC-rich	94.54	-1.736 to 190.8	No	ns	0.0553
SrrY269 Ctrl vs. SrrY269* THC-rich*	-5.012	-94.96 to 84.94	No	ns	0.9986

Sample size and exclusions

Figure / Analysis	Age	Group	Overall cohort	Same animals across time points	Final n analyzed	Technical exclusion?	Reason for n variation / exclusion
Figure 4C – Glutamate Hippocampus	P120	Srr+/+ Ctrl	Yes	Partially overlapping	6	No	Valid dataset for glutamate quantification
Figure 4C – Glutamate Hippocampus	P120	Srr+/+ THC-rich	Yes	Partially overlapping	5	No	Valid dataset for glutamate quantification
Figure 4C – Glutamate Hippocampus	P120	SrrY269* Ctrl	Yes	Partially overlapping	7	No	Valid dataset for glutamate quantification
Figure 4C – Glutamate Hippocampus	P120	SrrY269* THC-rich	Yes	Partially overlapping	4	No	Valid dataset for glutamate quantification

Table Analyzed	Figure 4D - Glycine Hippocampus

Normality test

Group	Shapiro-Wilk test p value	n
Srr+/+ Ctrl	0.5857	7
Srr+/+ THC-rich	0.3299	5
SrrY269* Ctrl	0.9998	7
SrrY269* THC-rich	0.4386	5

Main statistical analysis

Analysis item	Result	P value
Statistical test	Two-way ANOVA	
Post hoc test	Tukey's multiple comparisons test	
Interaction	F(1, 20) = 0.003822	P = 0.9513
Genotype	F(1, 20) = 7.602	P = 0.0122
Treatment	F(1, 20) = 1.023	P = 0.3239

Sample size and exclusions

Figure / Analysis	Age	Group	Overall cohort	Same animals across time points	Final n analyzed	Technical exclusion?	Reason for n variation / exclusion
Figure 4D – Glycine Hippocampus	P120	Srr+/+ Ctrl	Yes	Partially overlapping	7	No	Valid dataset for glycine quantification
Figure 4D – Glycine Hippocampus	P120	Srr+/+ THC-rich	Yes	Partially overlapping	5	No	Valid dataset for glycine quantification
Figure 4D – Glycine Hippocampus	P120	SrrY269* Ctrl	Yes	Partially overlapping	7	No	Valid dataset for glycine quantification
Figure 4D – Glycine Hippocampus	P120	SrrY269* THC-rich	Yes	Partially overlapping	5	No	Valid dataset for glycine quantification

Table Analyzed	Figure 5B - Branches Hippocampus

Normality test

Group	Shapiro-Wilk test p value	n
Srr+/+ Ctrl	0.3008	4
Srr+/+ THC-rich	0.1945	4
SrrY269* Ctrl	0.1945	4
SrrY269* THC-rich	0.2124	4

Main statistical analysis

Analysis item	Result	P value
Statistical test	Two-way ANOVA	
Post hoc test	Tukey’s multiple comparisons test	
Interaction	F(1, 12) = 10.77	P = 0.0066
Genotype	F(1, 12) = 18.26	P = 0.0011
Treatment	F(1, 12) = 10.95	P = 0.0062

Multiple Comparisons

Tukey's multiple comparisons test	Predicted (LS) mean diff.	95.00% CI of diff.	Significant?	Summary	Adjusted P Value
Srr+/+ Ctrl vs. Srr+/+ THC-rich	-0.25	-39.43 to 38.93	No	ns	>0.9999
Srr+/+ Ctrl vs. SrrY269* Ctrl	70.5	31.32 to 109.7	Yes	***	0.0009
Srr+/+ Ctrl vs. SrrY269* THC-rich	9	-30.18 to 48.18	No	ns	0.9019
Srr+/+ THC-rich vs. SrrY269* Ctrl	70.75	31.57 to 109.9	Yes	***	0.0008
Srr+/+ THC-rich vs. SrrY269* THC-rich	9.25	-29.93 to 48.43	No	ns	0.8947
SrrY269 Ctrl vs. SrrY269* THC-rich*	-61.5	-100.7 to -22.32	Yes	**	0.0027

Sample size and exclusions

Figure / Analysis	Age	Group	Animals represented	Cells analyzed per animal	Statistical unit	Technical exclusion?
Figure 5B – Branches Hippocampus	P120	Srr+/+ Ctrl	4	4	animal mean	No
Figure 5B – Branches Hippocampus	P120	Srr+/+ THC-rich	4	4	animal mean	No
Figure 5B – Branches Hippocampus	P120	SrrY269* Ctrl	4	4	animal mean	No
Figure 5B – Branches Hippocampus	P120	SrrY269* THC-rich	4	4	animal mean	No

Table Analyzed	Figure 5C - Total branch length Hippocampus

Normality test

Group	Shapiro-Wilk test p value	n
Srr+/+ Ctrl	0.3675	4
Srr+/+ THC-rich	0.435	4
SrrY269* Ctrl	0.5862	4
SrrY269* THC-rich	0.9907	4

Main statistical analysis

Analysis item	Result	P value
Statistical test	Two-way ANOVA	
Post hoc test	Tukey’s multiple comparisons test	
Interaction	F(1, 12) = 79.52	P < 0.0001
Genotype	F(1, 12) = 12.77	P = 0.0038
Treatment	F(1, 12) = 37.86	P < 0.0001

Multiple Comparisons

Tukey's multiple comparisons test	Predicted (LS) mean diff.	95.00% CI of diff.	Significant?	Summary	Adjusted P Value
Srr+/+ Ctrl vs. Srr+/+ THC-rich	33.02	-17.13 to 83.18	No	ns	0.2575
Srr+/+ Ctrl vs. SrrY269* Ctrl	63.85	13.69 to 114.0	Yes	*	0.0121
Srr+/+ Ctrl vs. SrrY269* THC-rich	-116.2	-166.3 to -66.03	Yes	****	<0.0001
Srr+/+ THC-rich vs. SrrY269* Ctrl	30.82	-19.33 to 80.98	No	ns	0.3093
Srr+/+ THC-rich vs. SrrY269* THC-rich	-149.2	-199.4 to -99.05	Yes	****	<0.0001
SrrY269 Ctrl vs. SrrY269* THC-rich*	-180	-230.2 to -129.9	Yes	****	<0.0001

Sample size and exclusions

Figure / Analysis	Age	Group	Animals represented	Cells analyzed per animal	Statistical unit	Technical exclusion?
Figure 5C- Total branch length Hippocampus	P120	Srr+/+ Ctrl	4	4	animal mean	No
Figure 5C- Total branch length Hippocampus	P120	Srr+/+ THC-rich	4	4	animal mean	No
Figure 5C- Total branch length Hippocampus	P120	SrrY269* Ctrl	4	4	animal mean	No
Figure 5C- Total branch length Hippocampus	P120	SrrY269* THC-rich	4	4	animal mean	No

Table Analyzed	Figure 5D - Junctions Hippocampus

Normality test

Group	Shapiro-Wilk test p value	n
Srr+/+ Ctrl	0.1366	4
Srr+/+ THC-rich	0.7326	4
SrrY269* Ctrl	0.4931	4
SrrY269* THC-rich	0.8916	4

Main statistical analysis

Analysis item	Result	P value
Statistical test	Two-way ANOVA	
Post hoc test	Tukey's multiple comparisons test	
Interaction	F(1, 12) = 5.821	P = 0.0327
Genotype	F(1, 12) = 5.544	P = 0.0364
Treatment	F(1, 12) = 3.314	P = 0.0937

Multiple Comparisons

Tukey's multiple comparisons test	Predicted (LS) mean diff.	95.00% CI of diff.	Significant?	Summary	Adjusted P Value
Srr+/+ Ctrl vs. Srr+/+ THC-rich	3.813	-23.22 to 30.84	No	ns	0.9742
Srr+/+ Ctrl vs. SrrY269* Ctrl	30.69	3.660 to 57.72	Yes	*	0.0248
Srr+/+ Ctrl vs. SrrY269* THC-rich	3.438	-23.59 to 30.47	No	ns	0.9808
Srr+/+ THC-rich vs. SrrY269* Ctrl	26.88	-0.1527 to 53.90	No	ns	0.0515
Srr+/+ THC-rich vs. SrrY269* THC-rich	-0.375	-27.40 to 26.65	No	ns	>0.9999
SrrY269 Ctrl vs. SrrY269* THC-rich*	-27.25	-54.28 to -0.2223	Yes	*	0.0479

Sample size and exclusions

Figure / Analysis	Age	Group	Animals represented	Cells analyzed per animal	Statistical unit	Technical exclusion?
Figure 5D- Junctions Hippocampus	P120	Srr+/+ Ctrl	4	4	animal mean	No
Figure 5D- Junctions Hippocampus	P120	Srr+/+ THC-rich	4	4	animal mean	No
Figure 5D- Junctions Hippocampus	P120	SrrY269* Ctrl	4	4	animal mean	No
Figure 5D- Junctions Hippocampus	P120	SrrY269* THC-rich	4	4	animal mean	No

Table Analyzed	Figure 5F - Maximum intersections Hippocampus

Normality test

Group	Shapiro-Wilk test p value	n
Srr+/+ Ctrl	0.1669	4
Srr+/+ THC-rich	0.1796	4
SrrY269* Ctrl	0.1856	4
SrrY269* THC-rich	0.5092	4

Main statistical analysis

Analysis item	Result	P value
Statistical test	Two-way ANOVA	
Post hoc test	Tukey’s multiple comparisons test	
Interaction	F(1, 12) = 0.5290	P = 0.4810
Genotype	F(1, 12) = 9.549	P = 0.0094
Treatment	F(1, 12) = 0.1080	P = 0.7481

Multiple Comparisons

Tukey's multiple comparisons test	Predicted (LS) mean diff.	95.00% CI of diff.	Significant?	Summary	Adjusted P Value
Srr+/+ Ctrl vs. Srr+/+ THC-rich	2.25	-21.44 to 25.94	No	ns	0.9918
Srr+/+ Ctrl vs. SrrY269* Ctrl	-13.33	-37.03 to 10.36	No	ns	0.3793
Srr+/+ Ctrl vs. SrrY269* THC-rich	-19.29	-42.98 to 4.401	No	ns	0.1264
Srr+/+ THC-rich vs. SrrY269* Ctrl	-15.58	-39.28 to 8.110	No	ns	0.2583
Srr+/+ THC-rich vs. SrrY269* THC-rich	-21.54	-45.23 to 2.151	No	ns	0.0792
SrrY269 Ctrl vs. SrrY269* THC-rich*	-5.958	-29.65 to 17.73	No	ns	0.8764

Sample size and exclusions

Figure / Analysis	Age	Group	Animals represented	Cells analyzed per animal	Statistical unit	Technical exclusion?
Figure 5F- Maximum Intersections Hippocampus	P120	Srr+/+ Ctrl	4	3	animal mean	No
Figure 5F- Maximum Intersections Hippocampus	P120	Srr+/+ THC-rich	4	3	animal mean	No
Figure 5F- Maximum Intersections Hippocampus	P120	SrrY269* Ctrl	4	3	animal mean	No
Figure 5F- Maximum Intersections Hippocampus	P120	SrrY269* THC-rich	4	3	animal mean	No

Table Analyzed	Figure 5G - Radius of Maximum Intersections Hippocampus

Normality test

Group	Shapiro-Wilk test p value	n
Srr+/+ Ctrl	0.4807	4
Srr+/+ THC-rich	0.513	4
SrrY269* Ctrl	0.0844	4
SrrY269* THC-rich	0.7143	4

Main statistical analysis

Analysis item	Result	P value
Statistical test	Two-way ANOVA	
Post hoc test	Tukey's multiple comparisons test	
Interaction	F(1, 12) = 4.376	P = 0.0584
Genotype	F(1, 12) = 0.6792	P = 0.4260
Treatment	F(1, 12) = 22.01	P = 0.0005

Multiple Comparisons

Tukey's multiple comparisons test	Predicted (LS) mean diff.	95.00% CI of diff.	Significant?	Summary	Adjusted P Value
Srr+/+ Ctrl vs. Srr+/+ THC-rich	-3.417	-8.936 to 2.103	No	ns	0.3037
Srr+/+ Ctrl vs. SrrY269* Ctrl	1.667	-3.853 to 7.186	No	ns	0.8069
Srr+/+ Ctrl vs. SrrY269* THC-rich	-7.25	-12.77 to -1.731	Yes	**	0.0098
Srr+/+ THC-rich vs. SrrY269* Ctrl	5.083	-0.4360 to 10.60	No	ns	0.0747
Srr+/+ THC-rich vs. SrrY269* THC-rich	-3.833	-9.353 to 1.686	No	ns	0.22
SrrY269 Ctrl vs. SrrY269* THC-rich*	-8.917	-14.44 to -3.397	Yes	**	0.0021

Sample size and exclusions

Figure / Analysis	Age	Group	Animals represented	Cells analyzed per animal	Statistical unit	Technical exclusion?
Figure 5F- Radius of Maximum Hippocampus	P120	Srr+/+ Ctrl	4	3	animal mean	No
Figure 5F- Radius of Maximum Hippocampus	P120	Srr+/+ THC-rich	4	3	animal mean	No
Figure 5F- Radius of Maximum Hippocampus	P120	SrrY269* Ctrl	4	3	animal mean	No
Figure 5F- Radius of Maximum Hippocampus	P120	SrrY269* THC-rich	4	3	animal mean	No

Table Analyzed	Figure 6E - GFAP intensity/DAPI

Normality test

Group	Shapiro-Wilk test p value	n
Srr+/+ Ctrl	0.6947	6
Srr+/+ THC-rich	0.2189	7
SrrY269* Ctrl	0.0037	7
SrrY269* THC-rich	0.8057	7

Main statistical analysis

Analysis item	Result	P value
Statistical test	Two-way ANOVA	
Post hoc test	Tukey’s multiple comparisons test	
Interaction	$F(1, 23) = 60.31$	$P < 0.0001$
Genotype	$F(1, 23) = 4.842$	$P = 0.0381$
Treatment	$F(1, 23) = 0.2395$	$P = 0.6292$

Multiple Comparisons

Tukey's multiple comparisons test	Predicted (LS) mean diff.	95.00% CI of diff.	Significant?	Summary	Adjusted P Value
Srr+/+ Ctrl vs. Srr+/+ THC-rich	-2.108	-3.264 to -0.9517	Yes	***	0.0002
Srr+/+ Ctrl vs. SrrY269* Ctrl	-2.887	-4.043 to -1.731	Yes	****	<0.0001
Srr+/+ Ctrl vs. SrrY269* THC-rich	-0.4956	-1.652 to 0.6605	No	ns	0.6413
Srr+/+ THC-rich vs. SrrY269* Ctrl	-0.7791	-1.890 to 0.3316	No	ns	0.2392
Srr+/+ THC-rich vs. SrrY269* THC-rich	1.612	0.5014 to 2.723	Yes	**	0.0028
SrrY269 Ctrl vs. SrrY269* THC-rich*	2.391	1.281 to 3.502	Yes	****	<0.0001

Sample size and exclusions

Figure / Analysis	Age	Group	Final n analyzed	Technical exclusion?	Reason for n variation / exclusion
Figure 6E- GFAP intensity	P120	Srr+/+ Ctrl	6	No	Valid dataset for GFAP intensity quantification
Figure 6E- GFAP intensity	P120	Srr+/+ THC-rich	7	No	Valid dataset for GFAP intensity quantification
Figure 6E- GFAP intensity	P120	SrrY269* Ctrl	7	No	Valid dataset for GFAP intensity quantification
Figure 6E- GFAP intensity	P120	SrrY269* THC-rich	7	No	Valid dataset for GFAP intensity quantification

Table Analyzed	Figure 6F - GFAP +/ area

Normality test

Group	Shapiro-Wilk test p value	n
Srr+/+ Ctrl	0.5842	6
Srr+/+ THC-rich	0.5127	7
SrrY269* Ctrl	0.3796	7
SrrY269* THC-rich	0.7512	7

Main statistical analysis

Analysis item	Result	P value
Statistical test	Two-way ANOVA	
Post hoc test	Tukey's multiple comparisons test	
Interaction	$F(1, 23) = 0.1116$	$P = 0.7414$
Genotype	$F(1, 23) = 0.02504$	$P = 0.8757$
Treatment	$F(1, 23) = 0.7422$	$P = 0.3979$

Multiple Comparisons

Tukey's multiple comparisons test	Predicted (LS) mean diff.	95.00% CI of diff.	Significant?	Summary	Adjusted P Value
Srr+/+ Ctrl vs. Srr+/+ THC-rich	-1.071	-9.179 to 7.036	No	ns	0.9829
Srr+/+ Ctrl vs. SrrY269* Ctrl	0.3571	-7.750 to 8.464	No	ns	0.9993
Srr+/+ Ctrl vs. SrrY269* THC-rich	-2.071	-10.18 to 6.036	No	ns	0.8933
Srr+/+ THC-rich vs. SrrY269* Ctrl	1.429	-6.361 to 9.218	No	ns	0.9565
Srr+/+ THC-rich vs. SrrY269* THC-rich	-1	-8.789 to 6.789	No	ns	0.9842
SrrY269 Ctrl vs. SrrY269* THC-rich*	-2.429	-10.22 to 5.361	No	ns	0.8237

Sample size and exclusions

Figure / Analysis	Age	Group	Final n analyzed	Technical exclusion?	Reason for n variation / exclusion
Figure 6F- GFAP +/ area	P120	Srr+/+ Ctrl	6	No	Valid dataset for GFAP +/ area quantification
Figure 6F- GFAP +/ area	P120	Srr+/+ THC-rich	7	No	Valid dataset for GFAP +/ area quantification

Figure 6F- GFAP +/ area	P120	SrrY269* Ctrl	7	No	Valid dataset for GFAP +/ area quantification
Figure 6F- GFAP +/ area	P120	SrrY269* THC-rich	7	No	Valid dataset for GFAP +/ area quantification

Table Analyzed	Figure 6K - Cell body area

Normality test

Group	Shapiro-Wilk test p value	n
Srr+/+ Ctrl	0.0771	6
Srr+/+ THC-rich	0.0392	7
SrrY269* Ctrl	0.0554	7
SrrY269* THC-rich	0.0661	7

Main statistical analysis

Analysis item	Result	P value
Statistical test	Two-way ANOVA	
Post hoc test	Tukey’s multiple comparisons test	
Interaction	F(1, 23) = 34.10	P < 0.0001
Genotype	F(1, 23) = 0.5285	P = 0.4746
Treatment	F(1, 23) = 2.011	P = 0.1696

Multiple Comparisons

Tukey's multiple comparisons test	Predicted (LS) mean diff.	95.00% CI of diff.	Significant?	Summary	Adjusted P Value
Srr+/+ Ctrl vs. Srr+/+ THC-rich	-94.69	-148.8 to -40.55	Yes	***	0.0003
Srr+/+ Ctrl vs. SrrY269* Ctrl	-66.7	-120.8 to -12.57	Yes	*	0.0103
Srr+/+ Ctrl vs. SrrY269* THC-rich	-9.016	-63.15 to 45.12	No	ns	0.9977
Srr+/+ THC-rich vs. SrrY269* Ctrl	27.98	-24.03 to 80.00	No	ns	0.5819
Srr+/+ THC-rich vs. SrrY269* THC-rich	85.67	33.66 to 137.7	Yes	***	0.0005
SrrY269 Ctrl vs. SrrY269* THC-rich*	57.69	5.675 to 109.7	Yes	*	0.0241

Sample size and exclusions

Figure / Analysis	Age	Group	Animals represented	Cells analyzed per animal	Statistical unit	Technical exclusion?
Figure 6K- Cell body area	P120	Srr+/+ Ctrl	6	5	animal mean	No
Figure 6K- Cell body area	P120	Srr+/+ THC-rich	7	5	animal mean	No
Figure 6K- Cell body area	P120	SrrY269* Ctrl	7	5	animal mean	No
Figure 6K- Cell body area	P120	SrrY269* THC-rich	7	5	animal mean	No

Table Analyzed	Figure 6L - Cell body perimeter

Normality test

Group	Shapiro-Wilk test p value	n
Srr+/+ Ctrl	0.1071	6
Srr+/+ THC-rich	0.5766	7
SrrY269* Ctrl	0.6571	7
SrrY269* THC-rich	0.5126	7

Main statistical analysis

Analysis item	Result	P value
Statistical test	Two-way ANOVA	
Post hoc test	Tukey’s multiple comparisons test	
Interaction	F(1, 23) = 21.73	P = 0.0001
Genotype	F(1, 23) = 0.004299	P = 0.9483
Treatment	F(1, 23) = 0.01738	P = 0.8963

Multiple Comparisons

Tukey's multiple comparisons test	Predicted (LS) mean diff.	95.00% CI of diff.	Significant?	Summary	Adjusted P Value
Srr+/+ Ctrl vs. Srr+/+ THC-rich	-99.69	-182.7 to -16.68	Yes	*	0.0146
Srr+/+ Ctrl vs. SrrY269* Ctrl	-95.59	-178.6 to -12.57	Yes	*	0.02
Srr+/+ Ctrl vs. SrrY269* THC-rich	-1.378	-84.40 to 81.64	No	ns	>0.9999
Srr+/+ THC-rich vs. SrrY269* Ctrl	4.106	-75.65 to 83.87	No	ns	0.9989
Srr+/+ THC-rich vs. SrrY269* THC-rich	98.32	18.56 to 178.1	Yes	*	0.0119
SrrY269 Ctrl vs. SrrY269* THC-rich*	94.21	14.45 to 174.0	Yes	*	0.0165

Sample size and exclusions

Figure / Analysis	Age	Group	Animals represente	Cells analyzed per animal	Statistical unit	Technical exclusion?
Figure 6L- Cell body perimeter	P120	Srr+/+ Ctrl	6	5	animal mean	No
Figure 6L- Cell body perimeter	P120	Srr+/+ THC-rich	7	5	animal mean	No
Figure 6L- Cell body perimeter	P120	SrrY269* Ctrl	7	5	animal mean	No
Figure 6L- Cell body perimeter	P120	SrrY269* THC-rich	7	5	animal mean	No

Table Analyzed	Figure 6M - Primary processes number

Normality test

Group	Shapiro-Wilk test p value	n
Srr+/+ Ctrl	0.1899	6
Srr+/+ THC-rich	0.2798	7
SrrY269* Ctrl	0.8215	7
SrrY269* THC-rich	0.5086	7

Main statistical analysis

Analysis item	Result	P value
Statistical test	Two-way ANOVA	
Post hoc test	Tukey’s multiple comparisons test	
Interaction	F (1, 23) = 0.1051	P = 0.7487
Genotype	F (1, 23) = 11.22	P = 0.0028
Treatment	F (1, 23) = 0.03783	P = 0.8475

Multiple Comparisons

Tukey's multiple comparisons test	Predicted (LS) mean diff.	95.00% CI of diff.	Significant?	Summary	Adjusted P Value
Srr+/+ Ctrl vs. Srr+/+ THC-rich	0.04286	-1,329 to 1,414	No	ns	>0,9999
Srr+/+ Ctrl vs. SrrY269* Ctrl	1.214	-0,1571 to 2,586	No	ns	0.1032
Srr+/+ Ctrl vs. SrrY269* THC-rich	1.043	-0,3285 to 2,414	No	ns	0.2128
Srr+/+ THC-rich vs. SrrY269* Ctrl	1.171	-0,1462 to 2,489	No	ns	0.101
Srr+/+ THC-rich vs. SrrY269* THC-rich	1	-0,3176 to 2,318	No	ns	0.2145
SrrY269 Ctrl vs. SrrY269* THC-rich*	-0.1714	-1,489 to 1,146	No	ns	0.9994

Sample size and exclusions

Figure / Analysis	Age	Group	Animals represented	Cells analyzed per animal	Statistical unit	Technical exclusion?
Figure 6M- Primary processes number	P120	Srr+/+ Ctrl	6	5	animal mean	No
Figure 6M- Primary processes number	P120	Srr+/+ THC-rich	7	5	animal mean	No
Figure 6M- Primary processes number	P120	SrrY269* Ctrl	7	5	animal mean	No
Figure 6M- Primary processes number	P120	SrrY269* THC-rich	7	5	animal mean	No

Table Analyzed	Figure 6N - Primary processes length

Normality test

Group	Shapiro-Wilk test p value	n
Srr+/+ Ctrl	0.4029	6
Srr+/+ THC-rich	0.7462	7
SrrY269* Ctrl	0.6959	7
SrrY269* THC-rich	0.3607	7

Main statistical analysis

Analysis item	Result	P value
Statistical test	Two-way ANOVA	
Post hoc test	Tukey’s multiple comparisons test	
Interaction	F(1, 23) = 14.83	P = 0.0008
Genotype	F(1, 23) = 7.695	P = 0.0108
Treatment	F(1, 23) = 3.861	P = 0.0616

Multiple Comparisons

Tukey's multiple comparisons test	Predicted (LS) mean diff.	95.00% CI of diff.	Significant?	Summary	Adjusted P Value
Srr+/+ Ctrl vs. Srr+/+ THC-rich	1.759	-1.965 to 5.483	No	ns	0.5677
Srr+/+ Ctrl vs. SrrY269* Ctrl	6.181	2.457 to 9.905	Yes	***	0.0007
Srr+/+ Ctrl vs. SrrY269* THC-rich	0.755	-2.969 to 4.479	No	ns	0.9426
Srr+/+ THC-rich vs. SrrY269* Ctrl	4.422	0.8439 to 8.000	Yes	*	0.0117
Srr+/+ THC-rich vs. SrrY269* THC-rich	-1.004	-4.582 to 2.574	No	ns	0.8641
SrrY269 Ctrl vs. SrrY269* THC-rich*	-5.426	-9.004 to -1.848	Yes	**	0.0018

Sample size and exclusions

Figure / Analysis	Age	Group	Animals represente	Cells analyzed per animal	Statistical unit	Technical exclusion?
Figure 6N- Primary processes length	P120	Srr+/+ Ctrl	6	5	animal mean	No
Figure 6N- Primary processes length	P120	Srr+/+ THC-rich	7	5	animal mean	No
Figure 6N- Primary processes length	P120	SrrY269* Ctrl	7	5	animal mean	No
Figure 6N- Primary processes length	P120	SrrY269* THC-rich	7	5	animal mean	No

Table Analyzed	Figure 6O - Secondary processes number

Normality test

Group	Shapiro-Wilk test p value	n
Srr+/+ Ctrl	0.1951	6
Srr+/+ THC-rich	0.0389	7
SrrY269* Ctrl	0.1366	7
SrrY269* THC-rich	0.048	7

Main statistical analysis

Analysis item	Result	P value
Statistical test	Two-way ANOVA	
Post hoc test	Tukey's multiple comparisons test	
Interaction	F(1, 23) = 0.4297	P = 0.5189
Genotype	F(1, 23) = 1.202	P = 0.2848
Treatment	F(1, 23) = 1.964	P = 0.1750

Multiple Comparisons

Tukey's multiple comparisons test	Predicted (LS) mean diff.	95.00% CI of diff.	Significant?	Summary	Adjusted P Value
Srr+/+ Ctrl vs. Srr+/+ THC-rich	0.8667	-0.8504 to 2.584	No	ns	0.5116
Srr+/+ Ctrl vs. SrrY269* Ctrl	0.7381	-0.9165 to 2.393	No	ns	0.6098
Srr+/+ Ctrl vs. SrrY269* THC-rich	1.052	-0.6022 to 2.707	No	ns	0.3156
Srr+/+ THC-rich vs. SrrY269* Ctrl	-0.1286	-1.783 to 1.526	No	ns	0.9963
Srr+/+ THC-rich vs. SrrY269* THC-rich	0.1857	-1.469 to 1.840	No	ns	0.9892
SrrY269 Ctrl vs. SrrY269* THC-rich*	0.3143	-1.275 to 1.904	No	ns	0.9458

Sample size and exclusions

Figure / Analysis	Age	Group	Animals represented	Cells analyzed per animal	Statistical unit	Technical exclusion?	Reason for n variation / exclusion
Figure 6O - Secondary processes number	P120	Srr+/+ Ctrl	6	5	animal mean	No	Valid dataset for astrocyte -Secondary processes number analysis
Figure 6O - Secondary processes number	P120	Srr+/+ THC-rich	7	5	animal mean	No	Valid dataset for astrocyte -Secondary processes number analysis
Figure 6O - Secondary processes number	P120	SrrY269* Ctrl	7	5	animal mean	No	Valid dataset for astrocyte -Secondary processes number analysis
Figure 6O - Secondary processes number	P120	SrrY269* THC-rich	7	5	animal mean	No	Valid dataset for astrocyte -Secondary processes number analysis

Table Analyzed	Figure 6P - Primary processes width

Normality test

Group	Shapiro-Wilk test p value	n
Srr+/+ Ctrl	0.5663	6
Srr+/+ THC-rich	0.558	7
SrrY269* Ctrl	0.0921	7
SrrY269* THC-rich	0.4423	7

Main statistical analysis

Analysis item	Result	P value
Statistical test	Two-way ANOVA	
Post hoc test	Tukey’s multiple comparisons test	
Interaction	F(1, 23) = 334.5	P < 0.0001
Genotype	F(1, 23) = 0.3440	P = 0.5632
Treatment	F(1, 23) = 1.305	P = 0.2650

Multiple Comparisons

Tukey's multiple comparisons test	Predicted (LS) mean diff.	95.00% CI of diff.	Significant?	Summary	Adjusted P Value
Srr+/+ Ctrl vs. Srr+/+ THC-rich	-0.9833	-1.212 to -0.7544	Yes	****	<0.0001
Srr+/+ Ctrl vs. SrrY269* Ctrl	-1.082	-1.311 to -0.8536	Yes	****	<0.0001
Srr+/+ Ctrl vs. SrrY269* THC-rich	0.03188	-0.1970 to 0.2607	No	ns	0.98
Srr+/+ THC-rich vs. SrrY269* Ctrl	-0.09914	-0.3190 to 0.1207	No	ns	0.6039
Srr+/+ THC-rich vs. SrrY269* THC-rich	1.015	0.7953 to 1.235	Yes	****	<0.0001
SrrY269 Ctrl vs. SrrY269* THC-rich*	1.114	0.8944 to 1.334	Yes	****	<0.0001

Sample size and exclusions

Figure / Analysis	Age	Group	Animals represented	Cells analyzed per animal	Statistical unit	Technical exclusion?
Figure 6P - Primary processes width	P120	Srr+/+ Ctrl	6	5	animal mean	No
Figure 6P - Primary processes width	P120	Srr+/+ THC-rich	7	5	animal mean	No
Figure 6P - Primary processes width	P120	SrrY269* Ctrl	7	5	animal mean	No
Figure 6P - Primary processes width	P120	SrrY269* THC-rich	7	5	animal mean	No

Table Analyzed	Supplementary Figure 1A - PPI P55 75 dB

Normality test

Group	Shapiro-Wilk test p value	n
Srr+/+ Ctrl 75 dB/60ms	0.762	13
Srr+/+ THC-rich 75 dB/60ms	0.7071	12
Srr+/+ Ctrl 75 dB/120ms	0.0605	12
Srr+/+ THC-rich 75 dB/120ms	0.7727	14
SrrY269* Ctrl 75 dB/60ms	0.0417	13
SrrY269* THC-rich 75 dB/60ms	0.274	15
SrrY269* Ctrl 75 dB/120ms	0.2918	11
SrrY269* THC-rich 75 dB/120ms	0.2277	14

Main statistical analysis

Analysis item	Result	P value
Statistical test	Two-way ANOVA	
Post hoc test	Fisher's LSD	
Interstimulus interval × group	F(3, 96) = 0.6056	P = 0.6129
Interstimulus interval	F(1, 96) = 1.077	P = 0.3020
Group (genotype and treatment combined)	F(3, 96) = 2.230	P = 0.0896

Multiple Comparisons

Fisher's LSD	Mean Diff.	95.00% CI of diff.	Significant?	Summary	Individual P Value
Row 1					
Row 1					
Srr+/+ Ctrl vs. Srr+/+ THC-rich	9.817	-4.096 to 23.73	No	ns	0.259
Srr+/+ Ctrl vs. SrrY269* Ctrl	6.678	-6.954 to 20.31	No	ns	0.5773
Srr+/+ Ctrl vs. SrrY269* THC-rich	12.68	-0.4927 to 25.85	No	ns	0.0636
Srr+/+ THC-rich vs. SrrY269* Ctrl	-3.139	-17.05 to 10.77	No	ns	0.9349
Srr+/+ THC-rich vs. SrrY269* THC-rich	2.86	-10.60 to 16.32	No	ns	0.9448
SrrY269 Ctrl vs. SrrY269* THC-rich*	5.999	-7.171 to 19.17	No	ns	0.634
Row 2					
Srr+/+ Ctrl vs. Srr+/+ THC-rich	7.015	-6.657 to 20.69	No	ns	0.539
Srr+/+ Ctrl vs. SrrY269* Ctrl	5.202	-9.306 to 19.71	No	ns	0.7848
Srr+/+ Ctrl vs. SrrY269* THC-rich	3.702	-9.971 to 17.37	No	ns	0.8938
Srr+/+ THC-rich vs. SrrY269* Ctrl	-1.814	-15.82 to 12.19	No	ns	0.9866
Srr+/+ THC-rich vs. SrrY269* THC-rich	-3.314	-16.45 to 9.823	No	ns	0.9119
SrrY269 Ctrl vs. SrrY269* THC-rich*	-1.5	-15.50 to 12.50	No	ns	0.9923

Sample size and exclusions

Figure / Analysis	Age	Group	Overall cohort	Same animals across time points	Technical exclusion?	Reason for n variation / exclusion
Supplementary Figure 1A- PPI P55 75 dB	P55	Srr+/+ Ctrl	Yes	Partially overlapping	baseline startle response < 1	Subset analyzed at this age
Supplementary Figure 1A- PPI P55 75 dB	P55	Srr+/+ THC-rich	Yes	Partially overlapping	baseline startle response < 1	Subset analyzed at this age
Supplementary Figure 1A- PPI P55 75 dB	P55	SrrY269* Ctrl	Yes	Partially overlapping	baseline startle response < 1	Subset analyzed at this age
Supplementary Figure 1A- PPI P55 75 dB	P55	SrrY269* THC-rich	Yes	Partially overlapping	baseline startle response < 1	Subset analyzed at this age

Table Analyzed	Supplementary Figure 1B - PPI P90 75 dB

Normality test

Group	Shapiro-Wilk test p value	n
Srr+/+ Ctrl 75 dB/60ms	0.2831	15
Srr+/+ THC-rich 75 dB/60ms	0.588	17
Srr+/+ Ctrl 75 dB/120ms	0.2791	14
Srr+/+ THC-rich 75 dB/120ms	0.0343	17
SrrY269* Ctrl 75 dB/60ms	0.4821	12
SrrY269* THC-rich 75 dB/60ms	0.0673	13
SrrY269* Ctrl 75 dB/120ms	0.6874	12
SrrY269* THC-rich 75 dB/120ms	0.7287	13

Main statistical analysis

Analysis item	Result	P value
Statistical test	Two-way ANOVA	
Post hoc test	Fisher's LSD	
Interstimulus interval × group	F(3, 105) = 0.9534	P = 0.4177
Interstimulus interval	F(1, 105) = 1.456e-005	P = 0.9970
Group (genotype and treatment combined)	F(3, 105) = 1.249	P = 0.2959

Multiple Comparisons

Fisher's LSD	Mean Diff.	95.00% CI of diff.	Significant?	Summary	Individual P Value
Row 1					
Srr+/+ Ctrl vs. Srr+/+ THC-rich	0.6768	-9.318 to 10.67	No	ns	0.998
Srr+/+ Ctrl vs. SrrY269* Ctrl	2.35	-8.577 to 13.28	No	ns	0.9432
Srr+/+ Ctrl vs. SrrY269* THC-rich	1.639	-9.052 to 12.33	No	ns	0.9782
Srr+/+ THC-rich vs. SrrY269* Ctrl	1.673	-8.964 to 12.31	No	ns	0.9765
Srr+/+ THC-rich vs. SrrY269* THC-rich	0.9627	-9.432 to 11.36	No	ns	0.995
SrrY269 Ctrl vs. SrrY269* THC-rich*	-0.7106	-12.01 to 10.58	No	ns	0.9984
Row 2					
Srr+/+ Ctrl vs. Srr+/+ THC-rich	9.427	-0.7558 to 19.61	No	ns	0.0801
Srr+/+ Ctrl vs. SrrY269* Ctrl	4.466	-6.633 to 15.57	No	ns	0.7202
Srr+/+ Ctrl vs. SrrY269* THC-rich	6.655	-4.212 to 17.52	No	ns	0.3837
Srr+/+ THC-rich vs. SrrY269* Ctrl	-4.961	-15.60 to 5.677	No	ns	0.6172
Srr+/+ THC-rich vs. SrrY269* THC-rich	-2.771	-13.17 to 7.624	No	ns	0.8984
SrrY269 Ctrl vs. SrrY269* THC-rich*	2.189	-9.105 to 13.48	No	ns	0.9575

Sample size and exclusions

Figure / Analysis	Age	Group	Overall cohort	Same animals across time points	Technical exclusion?	Reason for n variation / exclusion
Supplementary Figure 1B - PPI P90 75 dB	P55	Srr+/+ Ctrl	Yes	Partially overlapping	baseline startle response < 1	Subset analyzed at this age
Supplementary Figure 1B - PPI P90 75 dB	P55	Srr+/+ THC-rich	Yes	Partially overlapping	baseline startle response < 1	Subset analyzed at this age
Supplementary Figure 1B - PPI P90 75 dB	P55	SrrY269* Ctrl	Yes	Partially overlapping	baseline startle response < 1	Subset analyzed at this age
Supplementary Figure 1B - PPI P90 75 dB	P55	SrrY269* THC-rich	Yes	Partially overlapping	baseline startle response < 1	Subset analyzed at this age

Table Analyzed	Supplementary Figure 1C - PPI P120 75 dB	

Normality test

Group	Shapiro-Wilk test p value	n
Srr+/+ Ctrl 75 dB/60ms	0.4923	15
Srr+/+ THC-rich 75 dB/60ms	0.0562	17
Srr+/+ Ctrl 75 dB/120ms	0.6639	16
Srr+/+ THC-rich 75 dB/120ms	0.0241	18
SrrY269* Ctrl 75 dB/60ms	0.8724	8
SrrY269* THC-rich 75 dB/60ms	0.7196	10
SrrY269* Ctrl 75 dB/120ms	0.2928	9
SrrY269* THC-rich 75 dB/120ms	0.2525	11

Main statistical analysis

Analysis item	Result	P value
Statistical test	Two-way ANOVA	
Post hoc test	Fisher's LSD	
Interstimulus interval × group	F(3, 96) = 0.2251	P = 0.8787
Interstimulus interval	F(1, 96) = 1.876	P = 0.1740
Group (genotype and treatment combined)	F(3, 96) = 1.252	P = 0.2953

Multiple Comparisons

Fisher's LSD	Mean Diff.	95.00% CI of diff.	Significant?	Summary	Individual P Value
Row 1					
Srr+/+ Ctrl vs. Srr+/+ THC-rich	0.6696	-10.82 to 12.16	No	ns	0.9987
Srr+/+ Ctrl vs. SrrY269* Ctrl	6.952	-7.243 to 21.15	No	ns	0.5775
Srr+/+ Ctrl vs. SrrY269* THC-rich	0.2812	-12.96 to 13.52	No	ns	>0.9999
Srr+/+ THC-rich vs. SrrY269* Ctrl	6.283	-7.620 to 20.18	No	ns	0.6399
Srr+/+ THC-rich vs. SrrY269* THC-rich	-0.3884	-13.31 to 12.53	No	ns	0.9998
SrrY269 Ctrl vs. SrrY269* THC-rich*	-6.671	-22.05 to 8.710	No	ns	0.6695
Row 2					
Srr+/+ Ctrl vs. Srr+/+ THC-rich	3.498	-7.643 to 14.64	No	ns	0.8444
Srr+/+ Ctrl vs. SrrY269* Ctrl	5.523	-7.988 to 19.03	No	ns	0.7093
Srr+/+ Ctrl vs. SrrY269* THC-rich	-1.99	-14.69 to 10.71	No	ns	0.9767
Srr+/+ THC-rich vs. SrrY269* Ctrl	2.025	-11.21 to 15.26	No	ns	0.9782
Srr+/+ THC-rich vs. SrrY269* THC-rich	-5.488	-17.90 to 6.921	No	ns	0.6557
SrrY269 Ctrl vs. SrrY269* THC-rich*	-7.513	-22.09 to 7.061	No	ns	0.5351

Sample size and exclusions

Figure / Analysis	Age	Group	Overall cohort	Same animals across time points	Technical exclusion?	Reason for n variation / exclusion
Supplementary Figure 1C- PPI P120 75 dB	P55	Srr+/+ Ctrl	Yes	Partially overlapping	baseline startle response < 1	Subset analyzed at this age
Supplementary Figure 1C- PPI P120 75 dB	P55	Srr+/+ THC-rich	Yes	Partially overlapping	baseline startle response < 1	Subset analyzed at this age
Supplementary Figure 1C- PPI P120 75 dB	P55	SrrY269* Ctrl	Yes	Partially overlapping	baseline startle response < 1	Subset analyzed at this age
Supplementary Figure 1C- PPI P120 75 dB	P55	SrrY269* THC-rich	Yes	Partially overlapping	baseline startle response < 1	Subset analyzed at this age

Table Analyzed	Supplementary Figure 2A - D-serine PFC

Normality test

Group	Shapiro-Wilk test p value	n
Srr+/+ Ctrl	0.8632	7
Srr+/+ THC-rich	0.9274	5
SrrY269* Ctrl	0.2388	7
SrrY269* THC-rich	0.1216	5

Main statistical analysis

Analysis item	Result	P value
Statistical test	Two-way ANOVA	
Post hoc test	Tukey’s multiple comparisons test	
Interaction	F(1, 20) = 0.1790	P = 0.6767
Genotype	F(1, 20) = 153.0	P < 0.0001
Treatment	F(1, 20) = 0.6798	P = 0.4194

Multiple Comparisons

Tukey's multiple comparisons test	Predicted (LS) mean diff.	95.00% CI of diff.	Significant?	Summary	Adjusted P Value
Srr+/+ Ctrl vs. Srr+/+ THC-rich	-0.2454	-1.024 to 0.5332	No	ns	0.814
Srr+/+ Ctrl vs. SrrY269* Ctrl	2.35	1.639 to 3.061	Yes	****	<0.0001
Srr+/+ Ctrl vs. SrrY269* THC-rich	2.271	1.492 to 3.050	Yes	****	<0.0001
Srr+/+ THC-rich vs. SrrY269* Ctrl	2.595	1.817 to 3.374	Yes	****	<0.0001
Srr+/+ THC-rich vs. SrrY269* THC-rich	2.516	1.675 to 3.357	Yes	****	<0.0001
SrrY269 Ctrl vs. SrrY269* THC-rich*	-0.07894	-0.8575 to 0.6996	No	ns	0.9918

Sample size and exclusions

Figure / Analysis	Age	Group	Overall cohort	Same animals across time points	Final n analyzed	Technical exclusion?	Reason for n variation / exclusion
Supplementary Figure 2A - D-serine PFC	P120	Srr+/+ Ctrl	Yes	Partially overlapping	7	No	Valid dataset for D-serine quantification
Supplementary Figure 2A - D-serine PFC	P120	Srr+/+ THC-rich	Yes	Partially overlapping	5	No	Valid dataset for D-serine quantification
Supplementary Figure 2A - D-serine PFC	P120	SrrY269* Ctrl	Yes	Partially overlapping	7	No	Valid dataset for D-serine quantification
Supplementary Figure 2A - D-serine PFC	P120	SrrY269* THC-rich	Yes	Partially overlapping	5	No	Valid dataset for D-serine quantification

Table Analyzed	Supplementary Figure 2B - L-serine PFC

Normality test

Group	Shapiro-Wilk test p value	n
Srr+/+ Ctrl	0.2835	6
Srr+/+ THC-rich	0.1783	5
SrrY269* Ctrl	0.0338	7
SrrY269* THC-rich	0.7995	4

Main statistical analysis

Analysis item	Result	P value
Statistical test	Two-way ANOVA	
Post hoc test	Tukey's multiple comparisons test	
Interaction	$F(1, 18) = 0.002892$	$P = 0.9577$
Genotype	$F(1, 18) = 1.334$	$P = 0.2632$
Treatment	$F(1, 18) = 0.3132$	$P = 0.5826$

Multiple Comparisons

Tukey's multiple comparisons test	Predicted (LS) mean diff.	95.00% CI of diff.	Significant?	Summary	Adjusted P Value
Srr+/+ Ctrl vs. Srr+/+ THC-rich	1.007	-5.439 to 7.452	No	ns	0.9704
Srr+/+ Ctrl vs. SrrY269* Ctrl	1.984	-3.938 to 7.906	No	ns	0.7804
Srr+/+ Ctrl vs. SrrY269* THC-rich	2.814	-4.057 to 9.685	No	ns	0.66
Srr+/+ THC-rich vs. SrrY269* Ctrl	0.9771	-5.256 to 7.210	No	ns	0.9701
Srr+/+ THC-rich vs. SrrY269* THC-rich	1.807	-5.333 to 8.948	No	ns	0.8897
SrrY269 Ctrl vs. SrrY269* THC-rich*	0.8303	-5.842 to 7.502	No	ns	0.9846

Sample size and exclusions

Figure / Analysis	Age	Group	Overall cohort	Same animals across time points	Final n analyzed	Technical exclusion?	Reason for n variation / exclusion
Supplementary Figure 2B - L-serine PFC	P120	Srr+/+ Ctrl	Yes	Partially overlapping	6	No	Valid dataset for L-serine quantification
Supplementary Figure 2B - L-serine PFC	P120	Srr+/+ THC-rich	Yes	Partially overlapping	5	No	Valid dataset for L-serine quantification
Supplementary Figure 2B - L-serine PFC	P120	SrrY269* Ctrl	Yes	Partially overlapping	7	No	Valid dataset for L-serine quantification
Supplementary Figure 2B - L-serine PFC	P120	SrrY269* THC-rich	Yes	Partially overlapping	4	No	Valid dataset for L-serine quantification

Table Analyzed	Supplementary Figure 2C - Glutamate PFC

Normality test

Group	Shapiro-Wilk test p value	n
Srr+/+ Ctrl	0.6386	7
Srr+/+ THC-rich	0.9651	5
SrrY269* Ctrl	0.8898	7
SrrY269* THC-rich	0.1904	5

Main statistical analysis

Analysis item	Result	P value
Statistical test	Two-way ANOVA	
Post hoc test	Tukey's multiple comparisons test	
Interaction	F(1, 20) = 0.9634	P = 0.3380
Genotype	F(1, 20) = 2.071	P = 0.1656
Treatment	F(1, 20) = 0.5199	P = 0.4792

Multiple Comparisons

Tukey's multiple comparisons test	Predicted (LS) mean diff.	95.00% CI of diff.	Significant?	Summary	Adjusted P Value
Srr+/+ Ctrl vs. Srr+/+ THC-rich	2.567	-36.44 to 41.57	No	ns	0.9977
Srr+/+ Ctrl vs. SrrY269* Ctrl	-4.508	-40.11 to 31.10	No	ns	0.9843
Srr+/+ Ctrl vs. SrrY269* THC-rich	-21.28	-60.29 to 17.72	No	ns	0.4406
Srr+/+ THC-rich vs. SrrY269* Ctrl	-7.075	-46.08 to 31.93	No	ns	0.9563
Srr+/+ THC-rich vs. SrrY269* THC-rich	-23.85	-65.98 to 18.28	No	ns	0.4093
SrrY269 Ctrl vs. SrrY269* THC-rich*	-16.78	-55.78 to 22.23	No	ns	0.6315

Sample size and exclusions

Figure / Analysis	Age	Group	Overall cohort	Same animals across time points	Final n analyzed	Technical exclusion?	Reason for n variation / exclusion
Supplementary Figure 2C- Glutamate PFC	P120	Srr+/+ Ctrl	Yes	Partially overlapping	7	No	Valid dataset for glutamate quantification
Supplementary Figure 2C- Glutamate PFC	P120	Srr+/+ THC-rich	Yes	Partially overlapping	5	No	Valid dataset for glutamate quantification
Supplementary Figure 2C- Glutamate PFC	P120	SrrY269* Ctrl	Yes	Partially overlapping	7	No	Valid dataset for glutamate quantification
Supplementary Figure 2C- Glutamate PFC	P120	SrrY269* THC-rich	Yes	Partially overlapping	5	No	Valid dataset for glutamate quantification

Table Analyzed	Supplementary Figure 2D - Glycine PFC

Normality test

Group	Shapiro-Wilk test p value	n
Srr+/+ Ctrl	0.3531	7
Srr+/+ THC-rich	0.637	5
SrrY269* Ctrl	0.9837	7
SrrY269* THC-rich	0.6439	4

Main statistical analysis

Analysis item	Result	P value
Statistical test	Two-way ANOVA	
Post hoc test	Tukey’s multiple comparisons test	
Interaction	F(1, 19) = 0.8495	P = 0.3683
Genotype	F(1, 19) = 0.2888	P = 0.5972
Treatment	F(1, 19) = 0.2579	P = 0.6174

Multiple Comparisons

Tukey's multiple comparisons test	Predicted (LS) mean diff.	95.00% CI of diff.	Significant?	Summary	Adjusted P Value
Srr+/+ Ctrl vs. Srr+/+ THC-rich	2.556	-4.308 to 9.419	No	ns	0.7246
Srr+/+ Ctrl vs. SrrY269* Ctrl	2.609	-3.657 to 8.874	No	ns	0.6519
Srr+/+ Ctrl vs. SrrY269* THC-rich	1.869	-5.478 to 9.215	No	ns	0.8898
Srr+/+ THC-rich vs. SrrY269* Ctrl	0.05288	-6.811 to 6.916	No	ns	>0.9999
Srr+/+ THC-rich vs. SrrY269* THC-rich	-0.687	-8.550 to 7.176	No	ns	0.9946
SrrY269 Ctrl vs. SrrY269* THC-rich*	-0.7399	-8.087 to 6.607	No	ns	0.9918

Sample size and exclusions

Figure / Analysis	Age	Group	Overall cohort	Same animals across time points	Final n analyzed	Technical exclusion?	Reason for n variation / exclusion
Supplementary Figure 2D - Glycine PFC	P120	Srr+/+ Ctrl	Yes	Partially overlapping	7	No	Valid dataset for glycine quantification
Supplementary Figure 2D - Glycine PFC	P120	Srr+/+ THC-rich	Yes	Partially overlapping	5	No	Valid dataset for glycine quantification
Supplementary Figure 2D - Glycine PFC	P120	SrrY269* Ctrl	Yes	Partially overlapping	7	No	Valid dataset for glycine quantification
Supplementary Figure 2D - Glycine PFC	P120	SrrY269* THC-rich	Yes	Partially overlapping	4	No	Valid dataset for glycine quantification

Table Analyzed	Supplementary Figure 3B - Branches PFC

Normality test

Group	Shapiro-Wilk test p value	n
Srr+/+ Ctrl	0.3173	4
Srr+/+ THC-rich	0.1995	4
SrrY269* Ctrl	0.3846	4
SrrY269* THC-rich	0.8658	4

Main statistical analysis

Analysis item	Result	P value
Statistical test	Two-way ANOVA	
Post hoc test	Tukey's multiple comparisons test	
Interaction	$F(1, 12) = 16.52$	$P = 0.0016$
Genotype	$F(1, 12) = 3.087$	$P = 0.1044$
Treatment	$F(1, 12) = 0.1361$	$P = 0.7186$

Multiple Comparisons

Tukey's multiple comparisons test	Predicted (LS) mean diff.	95.00% CI of diff.	Significant?	Summary	Adjusted P Value
Srr+/+ Ctrl vs. Srr+/+ THC-rich	-56.25	-120.2 to 7.652	No	ns	0.0915
Srr+/+ Ctrl vs. SrrY269* Ctrl	-35.13	-99.03 to 28.78	No	ns	0.3984
Srr+/+ Ctrl vs. SrrY269* THC-rich	32.35	-31.55 to 96.26	No	ns	0.4654
Srr+/+ THC-rich vs. SrrY269* Ctrl	21.13	-42.78 to 85.03	No	ns	0.7624
Srr+/+ THC-rich vs. SrrY269* THC-rich	88.6	24.70 to 152.5	Yes	**	0.0068
SrrY269 Ctrl vs. SrrY269* THC-rich*	67.48	3.577 to 131.4	Yes	*	0.0375

Sample size and exclusions

Figure / Analysis	Age	Group	Animals represented	Cells analyzed per animal	Statistical unit	Technical exclusion?
Supplementary Figure 3B - Branches PFC	P120	Srr+/+ Ctrl	4	4	animal mean	No
Supplementary Figure 3B - Branches PFC	P120	Srr+/+ THC-rich	4	4	animal mean	No
Supplementary Figure 3B - Branches PFC	P120	SrrY269* Ctrl	4	4	animal mean	No
Supplementary Figure 3B - Branches PFC	P120	SrrY269* THC-rich	4	3	animal mean	No

Table Analyzed	Supplementary 3C - Total branch length PFC

Normality test

Group	Shapiro-Wilk test p value	n
Srr+/+ Ctrl	0.421	4
Srr+/+ THC-rich	0.7693	4
SrrY269* Ctrl	0.5899	4
SrrY269* THC-rich	0.5003	4

Main statistical analysis

Analysis item	Result	P value
Statistical test	Two-way ANOVA	
Post hoc test	Tukey's multiple comparisons test	
Interaction	F(1, 12) = 14.74	P = 0.0024
Genotype	F(1, 12) = 6.032	P = 0.0303
Treatment	F(1, 12) = 3.658	P = 0.0800

Multiple Comparisons

Tukey's multiple comparisons test	Mean Diff.	95.00% CI of diff.	Significant?	Summary	Adjusted P Value
Srr+/+ Ctrl vs. Srr+/+ THC-rich	-45.73	-145.4 to 53.96	No	ns	0.5443
Srr+/+ Ctrl vs. SrrY269* Ctrl	-32.83	-132.5 to 66.86	No	ns	0.7645
Srr+/+ Ctrl vs. SrrY269* THC-rich	103.7	4.035 to 203.4	Yes	*	0.0406
Srr+/+ THC-rich vs. SrrY269* Ctrl	12.91	-86.78 to 112.6	No	ns	0.9798
Srr+/+ THC-rich vs. SrrY269* THC-rich	149.5	49.77 to 249.2	Yes	**	0.0038
SrrY269 Ctrl vs. SrrY269* THC-rich*	136.6	36.86 to 236.2	Yes	**	0.0074

Sample size and exclusions

Figure / Analysis	Age	Group	Animals represented	Cells analyzed per animal	Statistical unit	Technical exclusion?
Supplementary 3C- Total branch length PFC	P120	Srr+/+ Ctrl	4	3	animal mean	No
Supplementary 3C- Total branch length PFC	P120	Srr+/+ THC-rich	4	4	animal mean	No
Supplementary 3C- Total branch length PFC	P120	SrrY269* Ctrl	4	4	animal mean	No
Supplementary 3C- Total branch length PFC	P120	SrrY269* THC-rich	4	3	animal mean	No

Table Analyzed	Supplementary Figure 3D - Junctions PFC

Normality test

Group	Shapiro-Wilk test p value	n
Srr+/+ Ctrl	0.1983	4
Srr+/+ THC-rich	0.1509	4
SrrY269* Ctrl	0.5283	4
SrrY269* THC-rich	0.6358	4

Main statistical analysis

Analysis item	Result	P value
Statistical test	Two-way ANOVA	
Post hoc test	Tukey’s multiple comparisons test	
Interaction	$F(1, 12) = 10.33$	$P = 0.0074$
Genotype	$F(1, 12) = 0.8953$	$P = 0.3627$
Treatment	$F(1, 12) = 6.938$	$P = 0.0218$

Multiple Comparisons

Tukey's multiple comparisons test	Mean Diff.	95.00% CI of diff.	Significant?	Summary	Adjusted P Value
Srr+/+ Ctrl vs. Srr+/+ THC-rich	-4.25	-35.03 to 26.53	No	ns	0.9757
Srr+/+ Ctrl vs. SrrY269* Ctrl	-16.63	-47.41 to 14.16	No	ns	0.4128
Srr+/+ Ctrl vs. SrrY269* THC-rich	26.25	-4.534 to 57.03	No	ns	0.1048
Srr+/+ THC-rich vs. SrrY269* Ctrl	-12.38	-43.16 to 18.41	No	ns	0.6421
Srr+/+ THC-rich vs. SrrY269* THC-rich	30.5	-0.2839 to 61.28	No	ns	0.0524
SrrY269 Ctrl vs. SrrY269* THC-rich*	42.88	12.09 to 73.66	Yes	**	0.0065

Sample size and exclusions

Figure / Analysis	Age	Group	Animals represented	Cells analyzed per animal	Statistical unit	Technical exclusion?
Supplementary Figure 3D - Junctions PFC	P120	Srr+/+ Ctrl	4	4	animal mean	No
Supplementary Figure 3D - Junctions PFC	P120	Srr+/+ THC-rich	4	4	animal mean	No
Supplementary Figure 3D - Junctions PFC	P120	SrrY269* Ctrl	4	4	animal mean	No
Supplementary Figure 3D - Junctions PFC	P120	SrrY269* THC-rich	4	3	animal mean	No

Table Analyzed	Supplementary Figure 3F - Maximum number of intersections PFC

Normality test

Group	Shapiro-Wilk test p value	n
Srr+/+ Ctrl	0.7912	4
Srr+/+ THC-rich	0.0012	4
SrrY269* Ctrl	0.1945	4
SrrY269* THC-rich	0.3869	4

Main statistical analysis

Analysis item	Result	P value
Statistical test	Two-way ANOVA	
Post hoc test	Tukey’s multiple comparisons test	
Interaction	F(1, 12) = 1.629	P = 0.2259
Genotype	F(1, 12) = 16.42	P = 0.0016
Treatment	F(1, 12) = 41.86	P < 0.0001

Multiple Comparisons

Tukey's multiple comparisons test	Mean Diff.	95.00% CI of diff.	Significant?	Summary	Adjusted P Value
Srr+/+ Ctrl vs. Srr+/+ THC-rich	33.25	15.23 to 51.27	Yes	***	0.0007
Srr+/+ Ctrl vs. SrrY269* Ctrl	-11.92	-29.94 to 6.106	No	ns	0.2544
Srr+/+ Ctrl vs. SrrY269* THC-rich	10.38	-7.647 to 28.40	No	ns	0.361
Srr+/+ THC-rich vs. SrrY269* Ctrl	-45.17	-63.19 to -27.14	Yes	****	<0.0001
Srr+/+ THC-rich vs. SrrY269* THC-rich	-22.88	-40.90 to -4.853	Yes	*	0.0124
SrrY269 Ctrl vs. SrrY269* THC-rich*	22.29	4.269 to 40.31	Yes	*	0.0146

Sample size and exclusions

Figure / Analysis	Age	Group	Animals represented	Cells analyzed per animal	Statistical unit	Technical exclusion?
Figure 3F- Maximum intersections PFC	P120	Srr+/+ Ctrl	4	3	animal mean	No
Figure 3F- Maximum intersections PFC	P120	Srr+/+ THC-rich	4	3	animal mean	No
Figure 3F- Maximum intersections PFC	P120	SrrY269* Ctrl	4	3	animal mean	No
Figure 3F- Maximum intersections PFC	P120	SrrY269* THC-rich	4	3	animal mean	No

Table Analyzed	Supplementary Figure 3G - Radius of Maximum Intersections PFC

Normality test

Group	Shapiro-Wilk test p value	n
Srr+/+ Ctrl	0.6441	4
Srr+/+ THC-rich	0.8114	4
SrrY269* Ctrl	0.3379	4
SrrY269* THC-rich	0.6617	4

Main statistical analysis

Analysis item	Result	P value
Statistical test	Two-way ANOVA	
Post hoc test	Tukey’s multiple comparisons test	
Interaction	F(1, 12) = 1.246	P = 0.2862
Genotype	F(1, 12) = 3.019	P = 0.1079
Treatment	F(1, 12) = 13.72	P = 0.0030

Multiple Comparisons

Tukey's multiple comparisons test	Mean Diff.	95.00% CI of diff.	Significant?	Summary	Adjusted P Value
Srr+/+ Ctrl vs. Srr+/+ THC-rich	-4.25	-11.15 to 2.647	No	ns	0.3072
Srr+/+ Ctrl vs. SrrY269* Ctrl	4.688	-2.209 to 11.58	No	ns	0.2349
Srr+/+ Ctrl vs. SrrY269* THC-rich	-3.229	-10.13 to 3.668	No	ns	0.5282
Srr+/+ THC-rich vs. SrrY269* Ctrl	8.938	2.041 to 15.83	Yes	*	0.0108
Srr+/+ THC-rich vs. SrrY269* THC-rich	1.021	-5.876 to 7.918	No	ns	0.9704
SrrY269 Ctrl vs. SrrY269* THC-rich*	-7.917	-14.81 to -1.020	Yes	*	0.0233

Sample size and exclusions

Figure / Analysis	Age	Group	Animals represented	Cells analyzed per animal	Statistical unit	Technical exclusion?
Supplementary Figure 3G - Radius of Maximum Intersections PFC	P120	Srr+/+ Ctrl	4	4	animal mean	No
Supplementary Figure 3G - Radius of Maximum Intersections PFC	P120	Srr+/+ THC-rich	4	4	animal mean	No
Supplementary Figure 3G - Radius of Maximum Intersections PFC	P120	SrrY269* Ctrl	4	4	animal mean	No
Supplementary Figure 3G - Radius of Maximum Intersections PFC	P120	SrrY269* THC-rich	4	3	animal mean	No
